# A self-reliance framework for identifying strategic advanced materials

**DOI:** 10.1038/s41467-026-73294-8

**Published:** 2026-05-21

**Authors:** Cristina Teixeira, Cian Gabbett, Kevin Synnatschke, Jonathan N. Coleman, Zdenek Sofer, Manuel J. Mendes, Elvira Fortunato, Rodrigo Martins, Luis Pereira, Adam G. Kelly

**Affiliations:** 1 CENIMAT/i3N, Department of Material Science, NOVA School of Science and Technology, Campus de Caparica, Caparica, Portugal; 2 School of Physics, CRANN & AMBER Research Centres, Trinity College Dublin, Dublin 2, Ireland; 3 Center for Advancing Electronics Dresden (CfAED) and Faculty of Chemistry and Food Chemistry, Technische Universität Dresden, Dresden, Germany; 4 Department of Inorganic Chemistry, University of Chemistry and Technology Prague, Prague, Czech Republic

**Keywords:** Electronic devices, Batteries, Solar cells, Funding, Electronic properties and materials

## Abstract

Global supply chain disruptions make securing raw materials for next-generation technologies an urgent priority. Raw materials are currently deemed ‘critical’ based on their supply risk and ‘strategic’ if vital for green or digital transitions. However, these frameworks do not yet cover advanced materials, the complex, engineered substances like nanomaterials that are the drivers of modern innovation. Here we introduce a self-reliance index that quantifies European autonomy for elements, compounds and devices, using import dependence, recycling rates and supplier concentration. Linking this index to the state-of-the-art performance of a broad range of advanced materials, across conductors, semiconductors, dielectrics, battery electrodes and photovoltaic layers, we find that high-performance materials based on heavily imported elements almost always have European-sourced substitutes with comparable performance. We propose defining ‘strategic advanced materials’ as those offering high performance through locally available inputs. This framework provides a selection tool for researchers and policymakers to strengthen supply chain resilience and drive European technological sovereignty.

## Abstract (web summary)

Global uncertainty demands technological sovereignty. The authors define ‘strategic advanced materials’ as those that achieve high-performance using local inputs, showing Europe can replace risky imports with resilient, home-grown alternatives.

## Introduction

Following World War II, the fabric of globalisation was woven from international trade liberalisation, ultra-low shipping costs, the outsourcing of manufacturing to regions with low labour costs, and a relative absence of major conflict among world powers. Institutions such as the European Coal and Steel Community (ECSC), an antecedent of the European Union, were founded on the principle that economic interdependence would render war “not merely unthinkable, but materially impossible,” as articulated in the 1950 Schuman Declaration^1^. Yet today, the eruption of trade wars following the United States’ pivot to nominally reindustrialise has led to a marked deterioration in trade relations that are unlikely to be repaired in the short term, resulting in various institutions now predicting a global recession^2^. In competitive sectors where margins are thin, businesses are unlikely to withstand arbitrarily applied tariffs, high inflation, or a sustained loss of consumer purchasing power. Moreover, this disruption only adds to a number of significant supply chain crises over the past five years, caused variously by a pandemic^3^, war^4^, human error^5^, aggressive cost cutting^6^, and piracy^7^.

In an uncertain landscape, securing access to the raw materials at the heart of advanced technologies is emerging as a pressing challenge. Technological innovation is often driven by new materials whose selection has traditionally prioritised cost metrics such as abundance or performance metrics such as electronic mobility or theoretical charge storage capacity. However, between trade wars and the persistence of conflict in regions critical to global supply lines, research strategies based on maximal self-reliance through the use of locally sourced materials will be critical to ensuring the scale up of new technologies. For example, despite magnesium ranking as the seventh most abundant element in Earth’s crust, its global supply remains heavily concentrated: China produces 66% of the world’s magnesite ore and refines 90% of all magnesium^8^. Such concentration dependencies are also well-known in the context of batteries, with half of the world’s cobalt located in Zambia and Congo, and 88% of the world’s lithium produced by China and Chile. Perhaps surprisingly, despite molybdenum disulfide (MoS_2_) being among the most studied two-dimensional materials, there is currently no mine producing molybdenite in Europe. While the Malmbjerg Molybdenum Project is being developed to meet 30% of Europe’s molybdenum demand^9^, it is located in Greenland, a Danish territory in which the United States is actively running influence campaigns^10^ with the stated aim of acquiring it ‘by any means.‘^11^ This further compounds supply pressures that began earlier with China’s revocation of export licences for a range of materials that includes molybdenum^12^.

The relationship between a raw material’s importance and its availability forms the basis of criticality assessments, in which raw materials are deemed ‘critical’ if they combine high economic value with a high risk of supply disruption. Two core dimensions of these assessments are the supply risk, encompassing geological, technical, environmental, social, and geopolitical factors, and economic importance, which estimates the impact of a potential disruption^13^. Derived from this categorisation are ‘strategic’ raw materials, whose importance is based on the relevance of a raw material for the green and digital transition, as well as defence and space applications. Criticality assessments are regularly produced by the European Commission^8^ and directly inform policy and legislation like the Critical Raw Materials Act of 2024^14^, which has the explicit goal of building resilient raw material supply chains.

Critical and strategic raw materials are essential for modern industries ranging from health to agriculture and defence, but technological breakthroughs and advances in state-of-the-art are often driven by advanced materials that are typically compounds or composites of elements ranging across the periodic table. Advanced materials are those that have been specifically engineered to exhibit performance beyond their raw form and encompass such classes as biomaterials, nanomaterials, electronic materials, smart and functional materials, and advanced composites. Assessing the criticality of these materials usually focuses on test cases, such as individual materials for a battery electrode^15^ or rare-earth elements^16,17^, meaning a method of propagating security of supply to the growing family of advanced materials is still missing. Addressing this knowledge gap has become increasingly urgent as funding programmes such as Horizon Europe now require applicants to demonstrate how future projects will reduce reliance on non-European sources. For such programmes to effectively nudge research into local materials, a practical selection tool is required to allow both research and industry to make secure material choices without compromising technological progress.

Here, we demonstrate how the mosaic of advanced materials can be incorporated into criticality assessments through the development of a self-reliance index based on geographic availability. This framework covers the elements from hydrogen to bismuth and employs a straightforward relationship between import reliance, recycling rates, and supplier concentration to compute the index. Importantly, an elemental index allows self-reliance to propagate from elements to compounds and devices, meaning this methodology can encompass the vast range of advanced materials, both extant and yet-to-be-synthesised. Taking nanomaterials as an example subset of advanced materials, we link the self-reliance scores of a broad range of elements and compounds to their state-of-the-art values across key performance metrics for electronics, batteries, and photovoltaics. We find that high-performers that rely on heavily imported elements almost always have European-sourced substitutes with comparable performance.

Since the technological state of the art in advanced materials is not dominated by imported materials, we propose defining a ‘strategic advanced material’ as one characterised by high technological performance achievable through locally available inputs. Such a definition moves the strategic focus of next-generation technologies away from global supply bottlenecks and toward the deliberate engineering of high-performance materials from locally accessible resources, thus strengthening the resilience of future supply chains. The aim of this framework is to support both short-term decision-making, such as the identification of contingency materials under supply disruptions, and long-term strategic planning, including the prioritisation of advanced materials for exploration and the design of targeted funding and research programmes.

## Results

### Elemental abundance and European reliance on imports

Over the past several decades, Europe’s dependence on trade to acquire elements that are not domestically available has been relatively free of risk. The percentage of an element’s supply that must be imported to meet demand is characterised by the import reliance, *IR*, which is the ratio of net imports to apparent consumption, such that

1 $${IR}=\,\frac{{Imports}-{Exports}}{{Domestic}\,{production}+({Imports}-{Exports})}$$

with negative values defaulting to 0. The import reliance is a key indicator in criticality assessments for raw materials^18,19^ and data are available from the Raw Materials Information System^20^, the Study on the Critical Raw Materials (CRM) for the European Union (latest edition 2023)^8^, and factsheets produced by the SCRREEN3 network (Solutions for Critical Raw Materials-European Expert Network)^21^.

Supplementary Table 1 lists the elements from hydrogen to bismuth with their respective primary mineralisation type and primary mineral. Many elements form native deposits, such as sulphur, gold, and silver, while others are extracted as by-products from processing primary ores. For example, the nuclear industry in France requires highly purified zirconium but the primary mineral, zircon (ZrSiO_4_), contains about 1 part in 50 of hafnium, which is separated out in such quantities that Europe has become a net exporter. Similarly, many elements are only produced as by-products of lead, zinc, and copper processing, such as gallium, germanium, selenium, indium, tellurium, and bismuth, meaning that these elements can only be obtained by having access to the primary ore and the capability to process and separate out its components. In the case of many elements, no deposits exist within Europe but once the primary ore is imported, it can be processed and purified domestically, with an example being borates imported from Türkiye and refined into boron in Germany.

**Fig. 1:**
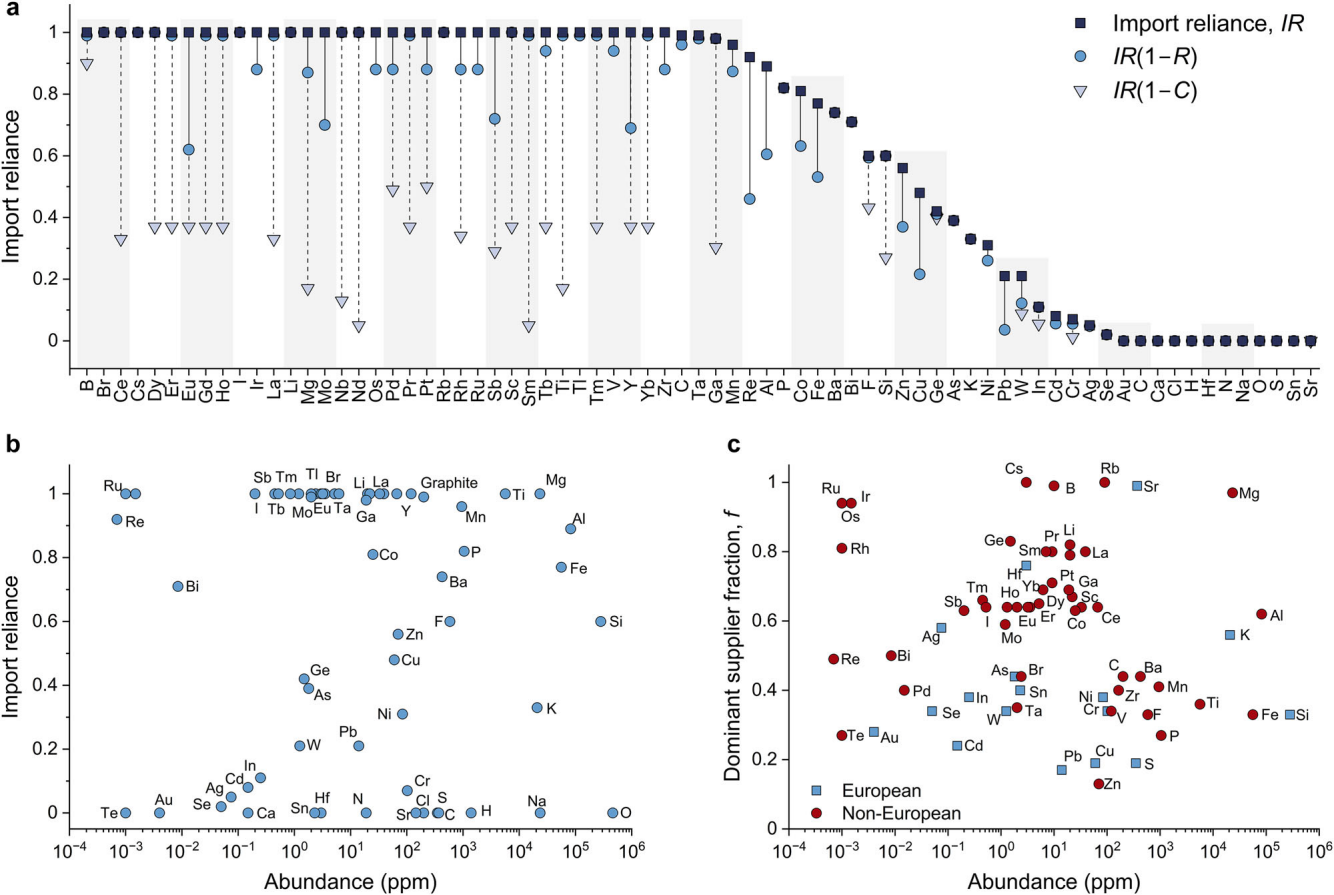
Import reliance and abundance. **a** The import reliance for each element (dark blue). Light blue circles represent the critical import fraction calculated using the end-of-life recycling input rate for the EU, *R*. The triangles represent the critical import fraction calculated using the EU collection rate, *C*. **b** The import reliance for each element as a function of crustal abundance. **c** The dominant supplier fraction for each element versus crustal abundance. All data are available in Supplementary Table 2.

Figure 1a shows the import reliance for each element to meet demand, ranked from high to low (square data points, see Supplementary Table 2). Once an element enters the European material ecosystem, recapture and recycling can reduce the amount that must be sourced externally. We therefore define the critical import reliance, *IR*_C_, as the proportion that must be imported and cannot be met at current recycling rates, such that *IR*_C_ = *IR*(1-*R*), where *R* is the European Union (EU) end-of-life recycling input rate (EoL RIR). While some materials have high recycling input rates, such as rhenium at 50%, the average across all values is ~ 10%. The resulting *IR*_C_ for each element (where data exist) is shown by the circular data points in Fig. 1a. This low reinput rate exists despite high collection efficiency as the average collection rate, *C*, across elements is ~62%. This highlights a major systemic gap in that material is efficiently gathered but not functionally returned to production. In a hypothetical, fully efficient circular scenario where all collected material is reinput (*R* = *C*), the import reliance for many fully imported elements could be drastically reduced. The potential *IR*_C_ in this idealised scenario, calculated as *IR*(1-*C*), is shown by the triangles in Fig. 1a.

While the EU demonstrates high efficiency in collecting end-of-life products containing a broad mix of materials, this often results in non-functional recycling where materials are recovered but are processed into forms unsuitable for their original applications. This is due to the immense difficulty in selectively isolating specific elements from complex waste streams, as exemplified by neodymium (Nd) and samarium (Sm) in Fig. 1a. These rare-earth magnets have collection rates of 95% but the current reinput rate into new production is virtually 0%. The disconnect between collection and reinput comes from a confluence of challenges, such as the technical complexity and cost of separating of target elements, the lack of EU-based refining capacity, and the economic non-viability of competing with primary virgin materials. Consequently, many collected elements are often consolidated into lower-value alloys rather than re-entering the production cycle. This issue has been addressed for strategic raw materials in the Critical Raw Material Act, which targets at least 25% of the EU’s annual consumption for recycling by 2030.

Figure 1b plots the import reliance for each element against its crustal abundance. This comparison demonstrates the need for a metric beyond abundance, as we find that Europe is heavily reliant on imports for many of the most abundant elements. Iron (77%), magnesium (99%), silicon metal (as high-purity elemental silicon is referred to) (60%), titanium metal (100%), and aluminium metal (89%) are all heavily imported. This reflects the economic reality that purifying raw ores to the levels required for a broad range of sectors can be costly, as an aluminium purity of 99.5% may be sufficient in the construction sector, but 99.999% (5 N) can be required in the semiconductor industry. To meet the current needs of the continent, even elements such as copper and zinc, which are widely mined across Europe, are also imported. One might also assume that a greater crustal abundance leads to a more diverse supplier base. However, Fig. 1c shows the largest fraction of EU imports from a single country (or dominant supplier fraction), *f*, plotted against abundance, again finding no relationship. Surprisingly, however, we find that the supply of some highly abundant elements, such as magnesium and aluminium is monopolised by a single supplier.

### Elemental self-reliance

**Fig. 2:**
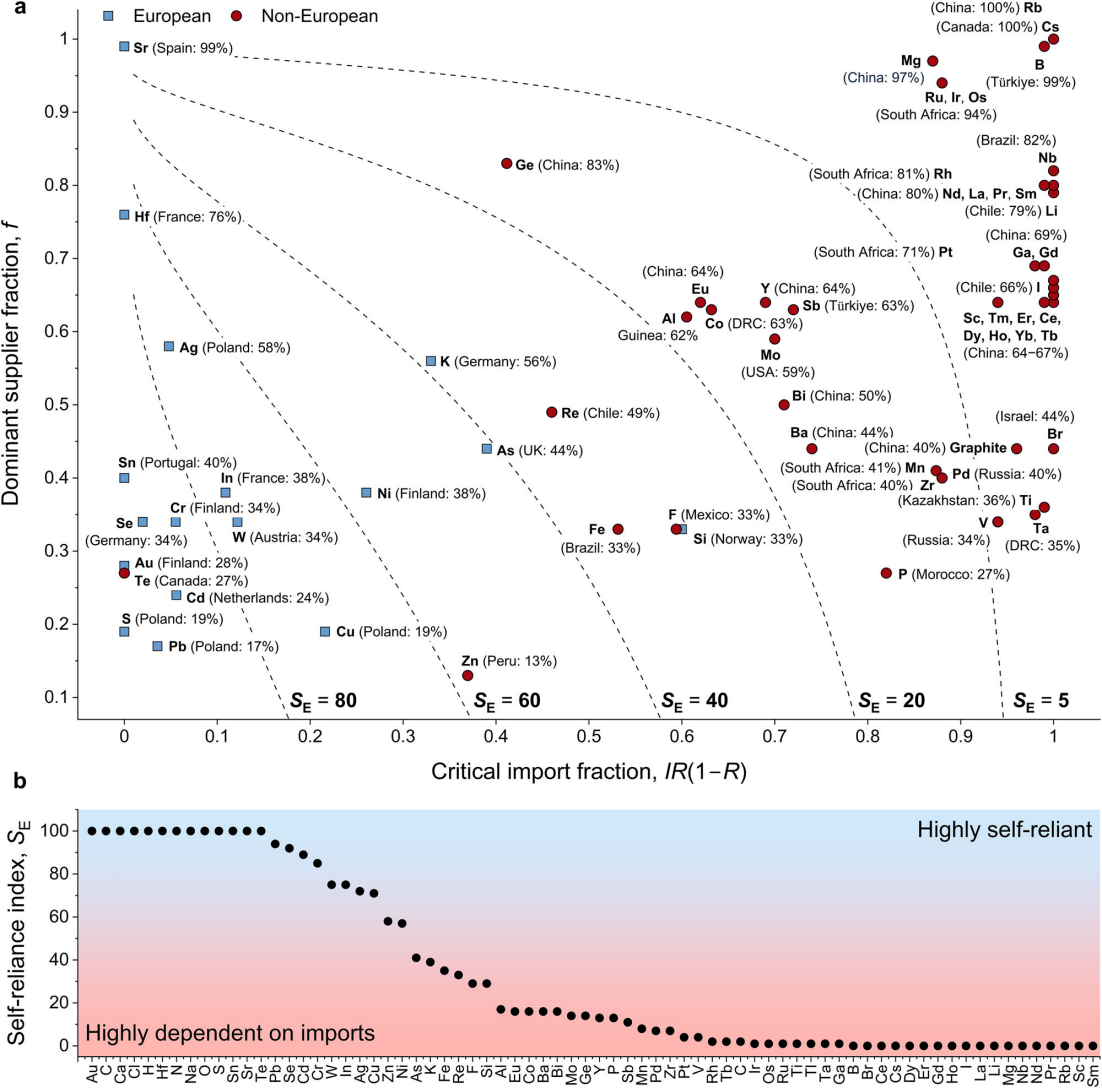
Supplier concentration and the self-reliance index. **a** The dominant supplier fraction, *f*, vs the critical import fraction. The country and supplier fraction are indicated in brackets. The dashed lines are curves of constant self-reliance index, *S*_*E*_, plotted using Eq. (2). **b** The self-reliance index computed for each element using Eq. (2), with values available in Supplementary Table 2 and Fig. 3.

To navigate the uncertainty of current and future trade relationships, a strategy of maximal self-reliance in the selection of materials for next-generation technologies may be the safest way to secure technological innovation. While the critical import fraction, *IR*_C_, identifies materials for which Europe is least dependent on external sources, the concentration of supply introduces an additional layer of vulnerability. Figure 2a shows the dominant supplier fraction, *f*, versus critical import fraction, where we see that those elements Europe imports in significant quantities also tend to have a single dominant supplier. Notably, more than 80% of European demand for rubidium, boron, caesium, magnesium, niobium, lithium, platinum group metals and several lanthanides is met by a single country. Under European jurisprudence^22^, such shares constitute strong evidence of market dominance, which applies to 38 of the 72 elements surveyed here and represents a substantial strategic dependency on non-European suppliers.

Based on these data, we propose an index for quantifying how self-reliant Europe is in a given element, considering the import reliance, *IR*, the end-of-life recycling input rate for the EU, *R*, and the dominant supplier fraction, *f*. We define the self-reliance index for an element, *S*_E_, as

2 $${S}_{{{{\rm{E}}}}}=100\left[1-{\left({IR}\left(1-R\right)\right)}^{1-f}\right]$$

where values are rounded to the nearest integer. With Eq. (2), the more of an element is recycled, the less is imported, and the larger the number of suppliers, the more self-reliant we consider Europe, with the metric varying between 0 (fully reliant on imports) and 100 (fully self-reliant). We plot lines of constant *S*_E_ in Fig. 2a to show the effect of using the dominant supplier fraction as an exponent rather than a linear term. Taking the example of germanium (Ge) and arsenic (As), both have a similar critical import fraction. However, as 83% of Europe’s Ge is supplied by China versus 44% of its As supplied by the United Kingdom, Ge falls in the region of 0 < *S*_E_ < 20 while As falls in the region of 40 < *S*_E_ < 60. The non-linearity conferred by the (1-*f*) exponent reflects the fact that when a market is dominated by a single supplier, even small disruptions can cause disproportionate shocks, and therefore markedly reduce an element’s self-reliance index when the critical import fraction is non-zero.

When critical raw materials must be sourced externally, suppliers are preferentially selected from stable partner countries, with stability typically assessed through metrics like the World Governance Indicators that are incorporated into supply risk calculations. While this approach is necessary when imports are unavoidable, such indicators primarily reflect a country’s internal institutional quality. They are less effective at capturing external geopolitical black-swan events that can disrupt trade, irrespective of a supplier’s domestic stability. For example, even a strategic partner like Japan, which scores highly on governance metrics, could see its trade with Europe severely restricted by a regional crisis, such as a conflict in the Taiwan Strait. A self-reliance index, by contrast, does not require such external indicators. Its purpose is instead to highlight elements that Europe can produce domestically and for which it can develop sovereign, or near-sovereign, supply chains.

**Fig. 3:**
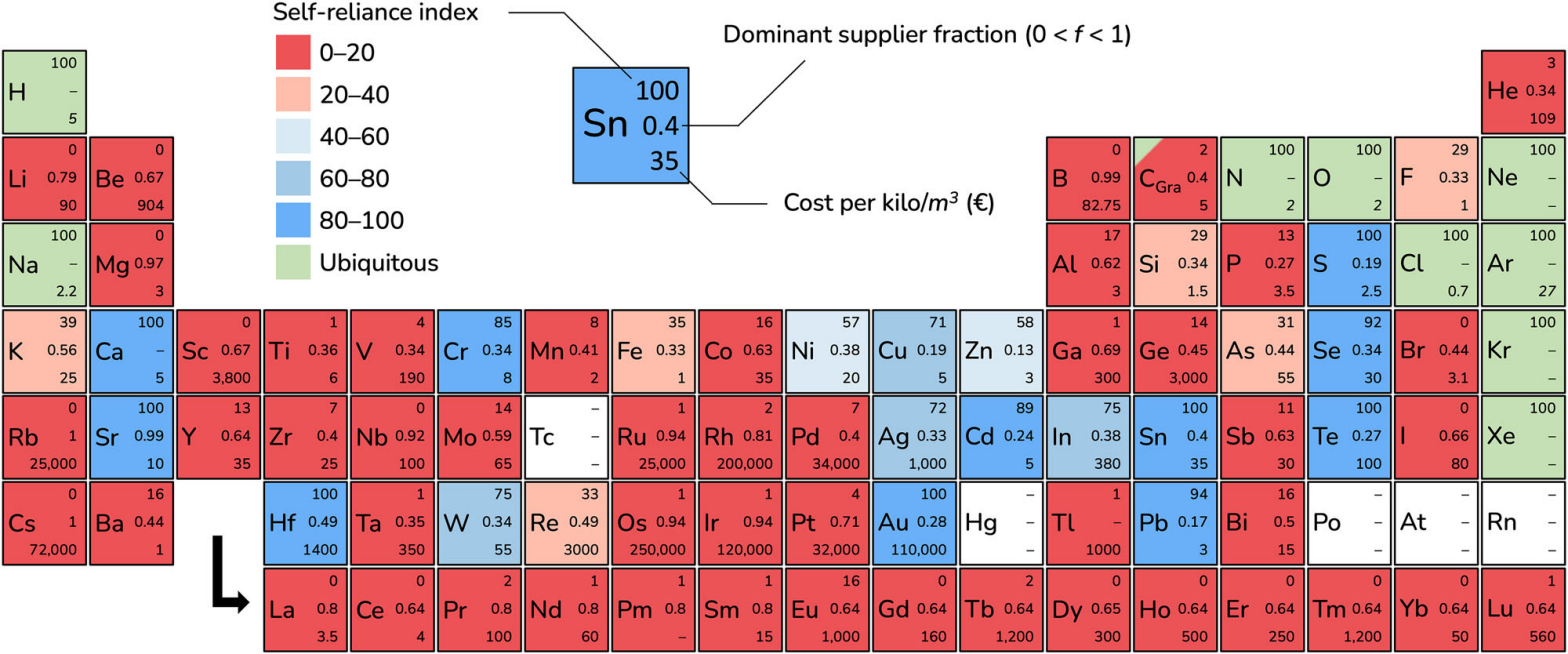
The periodic table. We visualise the self-reliance index, dominant supplier fraction, and approximate cost of each element, with all data available in Supplementary Tables 1 and 2. A version of this table with abundance in place of cost is available in Supplementary Fig. 1. Carbon is calculated using import reliance and recycling data for natural graphite (noting that elemental carbon is considered ubiquitous), fluorine using fluorspar, and potassium using potash.

We plot the *S*_E_ for each element in descending order in Fig. 2b and also summarise these data in the form of the periodic table in Fig. 3 to allow quick referencing of the self-reliance index, dominant supplier fraction, cost, and chemical properties to aid decision-making (Supplementary Fig. 1 contains the same table with abundance in place of cost). Europe is fully self-reliant in the chalcogens and also sodium, calcium, and strontium. We note that while Europe is self-reliant in hafnium, it is a by-product of zircon, as mentioned above, which is fully imported. Unsurprisingly, Europe is fully reliant on imports for rare-earth elements, platinum group metals, lithium, rubidium, and niobium, but also, surprisingly, for titanium, boron, iodine, and bromine. In the case of materials that are fully imported, Supplementary Table 2 also contains those European nations where deposits are known to exist, but are currently considered economically unviable.

Beyond building supply chain resilience through self-reliance, the environmental and ethical dimensions of material sourcing are critical. The environmental performance index (EPI)^23^ ranks nations on climate change, environmental health, and ecosystem vitality, with high scores indicating a greater likelihood of ethical and environmentally sound practices. As such, the index is often used as a country-level proxy for assessing extraction practices^24^. In Supplementary Fig. 2, we find a strong positive correlation between the elemental self-reliance index and the EPI score, meaning European-sourced materials are generally linked to greater environmental oversight. This provides additional motivation for a strategic shift toward European suppliers, as not only will such a shift strengthen the supply chain resilience, but it will also promote operators who are less likely to engage in environmentally damaging extraction or unethical exploitation practices.

The strength of an index based on individual elements lies in its ability be combined into the myriad combinations that form advanced material compounds. Methods to aggregate elemental supply risk into compounds typically use additive measures, such as the arithmetic mean, mass-share fraction, or maximum approach as applied in Refs. ^15^ and ^25^. Here, we argue the geometric mean is superior in the context of an advanced-material self-reliance index as it possesses a critical property: if any element in the compound is fully imported (*S*_E_ = 0), the compound’s overall score becomes zero. This reflects the fact that a shortage of one essential element renders the entire material unproducible, something that is not captured by arithmetic aggregation. Furthermore, the compound’s stoichiometry can be incorporated as exponents allowing the geometric mean to naturally weight each element’s contribution according to its molar share in the compound. For further discussion, see Supplementary Note 1.

Taking the example of a three-element compound with a chemical formula *A*_a_*B*_b_*C*_c_, its self-reliance index would be given by

3 $${S}_{C}={\left({\left({S}_{E,A}\right)}^{a}{\left({S}_{E,B}\right)}^{b}{\left({S}_{E,C}\right)}^{c}\right)}^{1/(a+b+c)}$$

where *S*_E_ is the self-reliance index of a given element in the compound formula and the power refers to its stoichiometry, and with resulting *S*_C_ values rounded to the nearest integer. For example, Bi_2_O_2_Se has *S*_E,A_ = 16, *S*_E,B_ = 100, *S*_E,C_ = 92, *a* = 2, *b* = 2, and *c* = 1, giving a self-reliance index for this compound of *S*_C_ = 47.

The example of Bi_2_O_2_Se raises the question of how ubiquitous elements such as oxygen should be treated as the value of *S*_C_ = 47 may be overinflated owing to oxygen constituting 2/5th of the compound. Existing criticality assessments focus on elements whose supply is constrained, and the concept of ubiquity has not required a strict definition. Advanced and nanostructured materials, however, differ fundamentally in that they intrinsically incorporate elements that are widely available and difficult to monopolise. In Supplementary Note 1, we define a ubiquitous element as one that is derived from planetary-scale reservoirs such as the air or oceans, is geographically and politically dispersed, is extractable and purifiable using simple and non-proprietary techniques, and is not susceptible to monopolisation. This definition identifies H, C, N, O, Na, Cl and the noble gases Ne, Ar, K, and Xe as ubiquitous elements, as highlighted in Fig. 3. When calculating a compound self-reliance index via the geometric mean, these elements are therefore treated neutrally and do not contribute the overall *S*_C_. In the case of Bi_2_O_2_Se above, this new consideration results in *S*_C_ = 29.

With the ability to apply the self-reliance index to both elements and compounds, we take the subclass of advanced materials called nanomaterials, those with at least one external dimension below 100 nm and with size-dependent properties, as a test case and create a link between European-sourced nanomaterials and their performance across various electronics.

### The self-reliance of electronic components

**Fig. 4:**
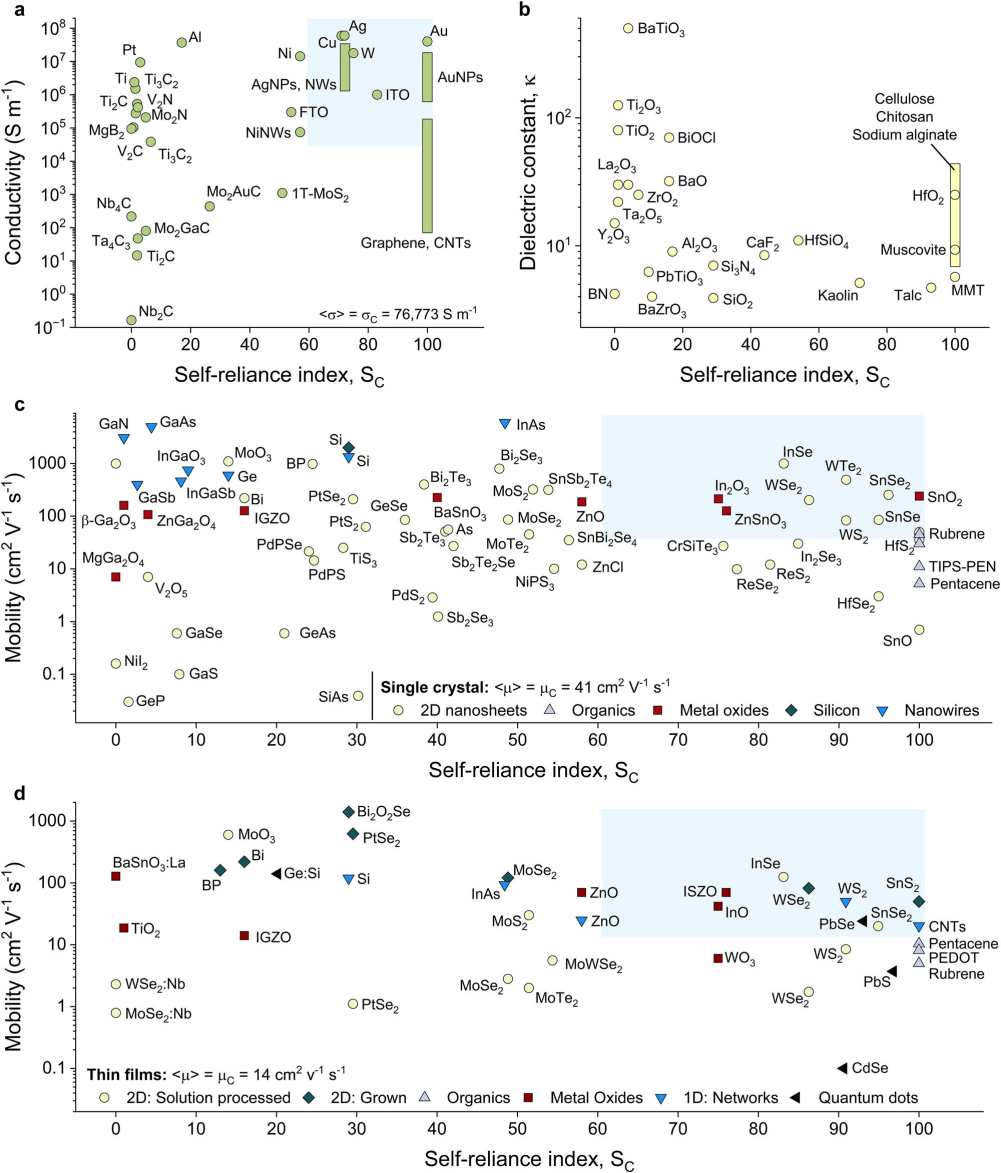
Electronic components. **a** The European self-reliance of conductive nanomaterials, with the geometric mean of all values, <*σ*>, set as the critical conductivity, *σ*_C_ = 76,773 S m^−1^. Values and references are available in Supplementary Table 3. **b** The dielectric constant of a range of insulators against their *S*_C_, with values and references available in Supplementary Table 4. **c** | The single-crystal mobilities of a range of semiconducting nanomaterials, with the geometric mean of all values, <*μ*>, set as the critical mobility, *μ*_C_ = 41 cm^2 ^V^−1^ s^−1^. Values and references are available in Supplementary Table 5. **d** The mobilities for thin films of a range of semiconducting nanomaterials, with *μ*_C_ = 14 cm^2 ^V^−1^ s^−1^. Values and references are available in Supplementary Table 6. Nanomaterials that fall within the blue regions bounded by the critical performance and *S*_C_ = 60, we designate as “strategic”.

We divide a selection of nanomaterials used in electronic components into conductors, dielectrics, and semiconductors. Figure 4a shows the conductivity of thin films of various conductive nanomaterials versus their self-reliance index (see Supplementary Table 3). The field of printed electronics relies on inks composed of silver or gold nanoparticles/nanowires, graphene, and carbon nanotubes to create electrodes in devices such as sensors, photodetectors and diodes, and each of these nanomaterials combine high conductivity with high self-reliance. Similarly, indium–tin oxide (ITO) and fluorine–tin oxide (FTO), widely used as transparent conductors in photovoltaic solar cells, also have similar conductivities and self-reliance. While the growing family of conductive 2D materials known as MXenes can show conductivities in the range of silver nanoparticles, they typically contain either titanium, niobium or vanadium, meaning their synthesis is heavily reliant on imported materials. We note that Cr-based MXenes and Hf-based MXenes have yet to be synthesised^26^ but, if created, would vastly improve Europe’s self-reliance for MXenes.

Insulating materials are key components in transistors, capacitors, and memory devices, with their dielectric constant, κ, affecting properties such as leakage currents, power consumption, and energy storage capacity. Figure 4b plots dielectric constant against *S*_C_ for a range of metal oxides, along with layered and polymeric materials (see Supplementary Table 4). SiO_2_ is the traditional gate dielectric for transistors, with a middling *S*_C_ owing to the import of high-purity silicon. Of the other oxides that have been widely explored as high-κ dielectrics, including Al_2_O_3_, Ti_2_O_3_, ZrO_2_, ZrO_2_ and HfO_2_, Europe is only self-reliant in HfO_2_. Boron nitride (BN) is perhaps the most studied 2D insulator; however, Europe is completely dependent on Türkiye for all of its boron. Sheet silicates, such as montmorillonite (MMT), mica, talc, and kaolin, are both abundant and widely available with dielectric constants in a similar range, while fluorspar (CaF_2_), being pursed for its ultra-wide bandgap of 12.1 eV, scores similarly to the oxides. Polymeric dielectrics, such as various types of cellulose, chitosan, and sodium alginate, are also important for printed electronics and tend to have hydration-dependent dielectric constants, with Europe being fully self-reliant owing to their sustainable origins.

Semiconducting materials are central to all of our electronics and are typically characterised by the charge-carrier mobility, a primary variable that affects transistor switching speed and power consumption. However, the mobility of a single-crystal semiconductor (e.g., a mechanically exfoliated 2D material) is almost always greater than that of a thin film (e.g., a spin-coated 2D material) owing to the formation of structural artefacts when scaling the material up to large areas^27^. Therefore, in Fig. 4c we relate the self-reliance index to the charge-carrier mobility of single crystals (to represent the maximum achievable values), while in Fig. 4d we relate the self-reliance index to the mobility of thin films (to reflect mobilities attained via scalable techniques like printing or chemical vapour deposition), with datasets available in Supplementary Tables 5 and 6.

In general, we find the semiconductors have values that are evenly distributed across Fig. 4c, 4d in both mobility and self-reliance index, which demonstrates that Europe can remain technologically competitive using materials found within its borders while also identifying specific materials to target for development. Interestingly, despite their high mobility and the significant amount of literature, Europe is highly dependent on imports for compounds comprising elements like molybdenum (Mo), platinum (Pt), germanium (Ge), or gallium (Ga). However, comparable mobilities can be achieved by substituting these elements for European-sourced tungsten (W), tin (Sn), indium (In), or hafnium (Hf). Single-crystal organic materials tend to have maximum mobilities of ~ 50 cm^2 ^V^−1^ s^−1^ owing to intrinsic material limitations, but, as organic materials, can be fully synthesised within Europe. Of the metal oxides, the mobilities are similar across all compositions, implying that material performance need not be sacrificed by focusing on Sn- or In-based compounds.

The highest mobilities are found in nanowires of the III–V semiconductors; however, their dependence on As, Ga, or antimony (Sb) means they will always be susceptible to supply chain crises. To ensure the graph remains readable, we highlight separately that carbon nanotubes have intrinsic mobilities >10^5^ (ref. ^28^) and, with a wide range of precursors available, have an *S*_E_ of 100. Similar to Fig. 4c, the most resilient thin films in Fig. 4d are those containing tin, tungsten, and organics, with reduced self-reliance for those 2D materials that are doped with heavily imported elements, such as niobium or lanthanum. Importantly, there is a cohort spanning all material classes that combines high self-reliance with high mobility, meaning Europe will not be limited by material class should it pursue locally sourced semiconductors.

In addition to the experimentally measured mobilities, we also sought 2D materials for which mobilities are only predicted. In Supplementary Note 2, we compute the self-reliance index for 119 n-type and 90 p-type semiconductors for which we found theoretical mobilities to provide guidance for future research (discussed further below). The data in Fig. 4 are quite promising for the future of European-based electronic nanomaterials, with high performance across conductors, dielectrics, and semiconductors not limited to a handful of exotic and expensive materials.

### The self-reliance of battery electrodes

**Fig. 5:**
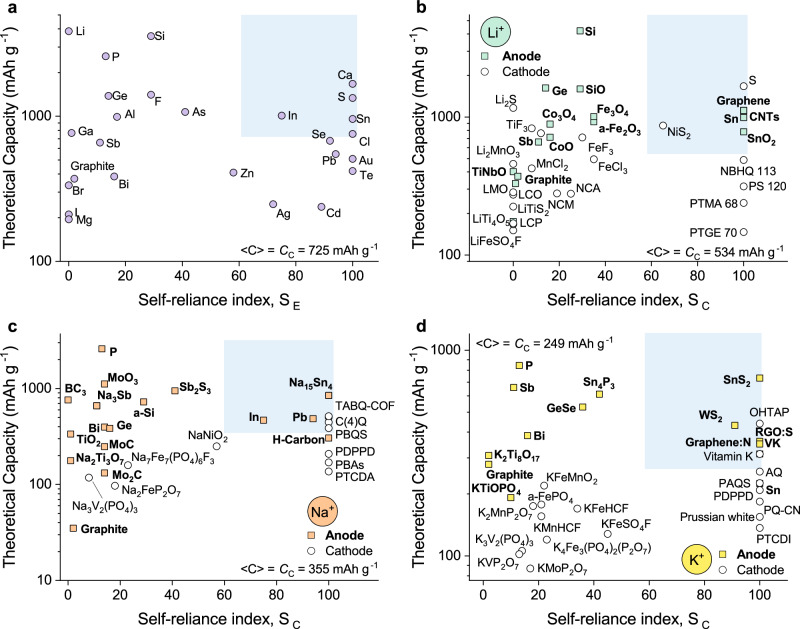
Battery electrodes. **a** The theoretical capacity for each of the elements calculated for Li (references in Supplementary Table 8), with the critical theoretical capacity, *C*_C_ = 725 mAh g^−1^. **b** The theoretical capacity for anodes and cathodes in lithium-ion batteries, including S form Li–S batteries (full list with references in Supplementary Table 8), with *C*_C_ = 534 mAh g^−1^. **c** Theoretical capacity of anodes and cathodes in sodium-ion batteries, where organic electrodes show the highest self-reliance (full list with references in Supplementary Table 9), with *C*_C_ = 355 mAh g^−1^. **d** The theoretical capacity of anodes and cathodes in potassium-ion batteries (full list with references in Supplementary Table 10) and with *C*_C_ = 249 mAh g^−1^. Nanomaterials that fall within the blue regions bounded by the critical performance shown inset (calculated using the geometric mean in all cases) and *S*_E_ = 60 we designate as “strategic”.

Next, we consider self-reliance in the context of electrode materials for lithium-ion batteries (LiIBs) and related energy storage systems (see Supplementary Table 8). By considering the reactions between lithium ions and active materials within battery electrodes, it is possible to quantify the theoretical lithium storage capacity for each element^29^. This theoretical capacity is plotted versus elemental self-reliance in Fig. 5a, illustrating a number of interesting points. Firstly, Europe is self-reliant in a number of reasonably high-capacity elements, notably sulphur, which is likely to become important owing to the significant energy densities offered by lithium–sulphur batteries^30,31^. However, the highest capacity elements, silicon (Si) and phosphorous (P), have *S*_E_ values below 50. In addition, silicon’s low *S*_E_ is problematic given its status as the most promising future anode material for LiIBs, with most of the global supply of purified silicon coming from China (76%). Moreover, Europe is even more dependent on imports for the current most common anode material in LiIBs, graphite, again owing to the global dominance of Chinese exports of spherical graphite (67%). This graph also highlights the fact that Europe is fully dependent on imports for lithium itself, meaning the continent can never sustain itself in LiIB production unless new domestic reserves are found.

Despite these high values, most electrode materials used in LiIBs are compounds rather than elemental materials. As shown in Fig. 5b, the theoretical capacity for a range of active material compounds commonly used in LiIBs is plotted versus the compound self-reliance, *S*_C_. Here we find that, broadly speaking, Europe is self-reliant in the higher capacity compounds than those with lower capacity, noting that graphene has a supply resilience of 100 owing to its diverse synthesis options compared to the current monopoly on naturally occurring spherical graphite. In addition, there are a number of materials suitable for use in both cathodes and anodes which have an *S*_C_ above 60, which is relatively positive for battery manufacturing in the EU. Of particular note here are the organic electrodes, which range from carbonyl or organo-sulphur compounds to conductive polymers and organic radicals^32^. While most are produced from petrochemicals, some can be synthesised from biomass or bulk industrial chemicals, making Europe fully self-reliant, albeit with generally lower theoretical capacities than inorganic materials.

Owing to lithium’s cost, concentrated production, and environmentally-damaging extraction processes, it is likely that LiIBs will eventually be replaced by sodium-ion batteries (NaIBs) and potassium-ion batteries (KIBs), especially in non-vehicle applications where gravimetric capacity is not critical. Indeed, we find that the *S*_E_ values for sodium (Na) and potassium (K) (100 and 67, respectively) are considerably higher than those of Li, while such materials are also much less reliant on Co than LiIBs, an additional advantage. Figure 5c, 5d plots a range of anodes and cathodes for each ion type, with a broad range of indices for both (see Supplementary Tables 9 and 10). While Europe heavily imports the highest capacity material, P, for both NaIBs and KIBs, there is a broad cohort of inorganic cathode and anode materials that combine high capacity and high values of *S*_C_. However, as with the LiIBs, both NaIBs and KIBs have a broad range of organic materials available as cathodes, with well-known materials such as Prussian blue (and its analogues) and Vitamin K used in KIBs. Such materials again add significant self-reliance to their respective batteries, implying that beyond-lithium battery manufacturing in Europe should be quite resilient to supply shocks.

### The self-reliance of photovoltaic layers

**Fig. 6:**
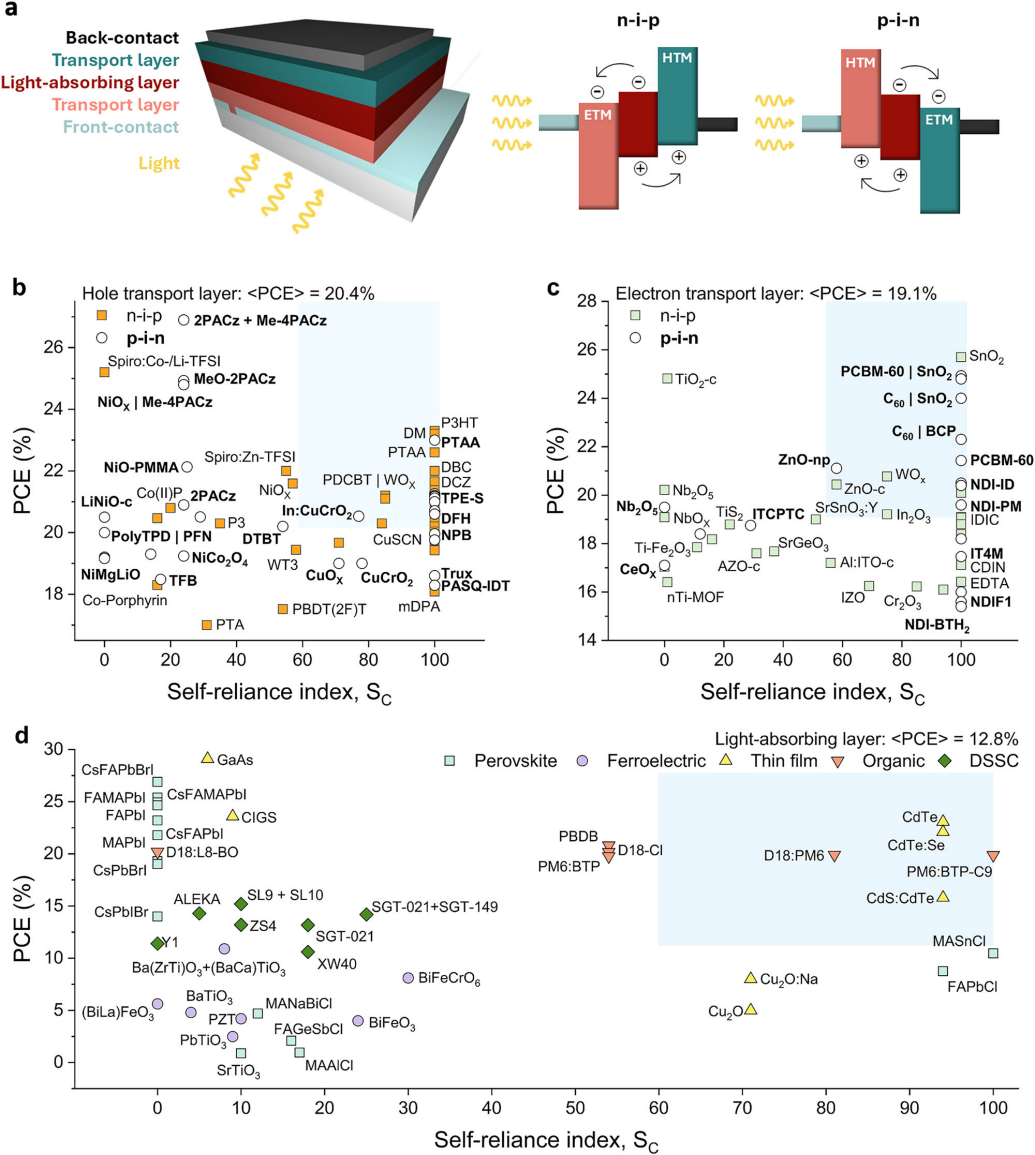
Photovoltaic layers. **a** Schematics of the types of photovoltaic cells considered in the main text. **b** The power conversion efficiency (PCE) of perovskite solar cells with the indicated hole transport layers for n-i-p (plain text) and p-i-n (in bold) configurations, with a critical PCE of 20.4 %. A selection of data points is labelled, with the full list available in Supplementary Tables 11 and 12. **c** The power conversion efficiency of perovskite cells containing the indicated electron transport layers, with n-i-p configurations indicated in plaintext and p-i-n configurations indicated in bold. The critical PCE here is 19.1 %. An indicative selection of data points is labelled, with the full list available in Supplementary Tables 13 and 14. **d** The self-reliance index of a range of a range of light-absorbing layers across several cell types, with a critical PCE of 12.8%. All references are available in Supplementary Table 15. Nanomaterials that fall within the blue regions bounded by the critical performance shown in the inset (calculated using the arithmetic mean in all cases) and *S*_E_ = 60, we designate as “strategic”. DSSC = Dye-sensitised solar cell.

Along with batteries, photovoltaics (PVs) are a key pillar of the global clean energy transition, but their supply chain is highly vulnerable. By the end of 2025, China was projected to control nearly 95% of global production of polysilicon, silicon ingots, and wafers for PV modules^33^. PV technologies are commonly categorised into three generations: the first is dominated by mature silicon-based cells; the second includes the thin-film technologies such as cadmium telluride (CdTe), copper–indium–gallium–selenide (CIGS), and amorphous silicon; and the third generation encompasses emerging PV technologies designed to surpass the efficiency limits of traditional cells. These include perovskites, along with dye-sensitised, organic, and ferroelectric solar cells, and also advanced materials like gallium arsenide (GaAs). In this section, we focus on the second and third generations, examining both the light-absorbing materials and the selective extraction materials used in n-i-p and p-i-n configurations shown in Fig. 6a, which are core components of these cells.

Given the intense current research and investment in perovskite technology, Fig. 6b and 6c show the maximum reported power conversion efficiencies (PCE) for a range of hole and electron transport materials used in perovskite solar cells, respectively, across both n-i-p and p-i-n configurations (Supplementary Tables 11–14). These configurations impose restrictions on material selection: in a p-i-n solar cell, the hole transport material (HTM) must be transparent to allow light to pass, whereas in an n-i-p configuration, it can be opaque, but must be deposited from an anhydrous solvent and annealed below 100 °C to avoid degrading the underlying perovskite layer.

We find that many of the top-performing HTMs are solely organic, contributing to high self-reliance. Europe is fully self-reliant in materials such as poly(3-hexylthiophene-2,5-diyl) (P3HT), (N2,N2′, N7,N7′ -tetrakis(9,9-dimethyl-9H-fluoren-2-yl)-N2,N2′,N7,N7′ - tetrakis(4-methoxyphenyl)-9,9′-spirobi[fluorene]-2,2′,7,7′- tetraamine) (DM), and poly(triarylamine) (PTAA) in the n-i-p architecture, with the highest performing material 2,2′,7,7′-tetrakis(N,N-di-p-methoxyphenylamine)-9,9′-spirobifluorene (Spiro-OMeTAD) becoming fully dependent on imports when doped with lithium salts. In the p-i-n configuration, the leading HTM is 4-(3,6-dimethyl-9H-carbazol-9-yl)butylphosphonic acid (Me-4PACz), while PTAA is Europe’s most self-reliant high-performance material. In contrast, the electron transport materials (ETMs) used in n-i-p structures are often metal oxides, valued for their stability, performance, and cost-effectiveness. Here, we find that Europe is fully self-reliant in the highest-performing material, tin (IV) oxide (SnO_2_), while titanium dioxide (TiO_2_), the most widely used, is far more dependent on imports for its titanium metal. For p-i-n devices, the dominant ETMs are fullerenes combined with SnO_2_, in which, again, Europe is 100% self-reliant.

Figure 6d shows the self-reliance index of the absorption layers for a range of photovoltaic technologies (see Supplementary Table 15), where it is immediately evident that this layer is the main supply-chain vulnerability for perovskite PV cells. High efficiency ( > 25 %) perovskite cells tend to depend on caesium, now extracted only intermittently from a single Canadian mine, with global supply now dependent on stockpiles, and iodine, which is almost exclusively sourced from Chile and Japan^34^. Europe has no known domestic sources of either caesium or iodine, which emphasises the importance of robust trade agreements with stable countries to support the continent’s growing perovskite start-up ecosystem. While Europe is significantly more self-reliant in iodine-free perovskites, their power conversion efficiencies typically remain below 15%, which limits their current competitiveness but suggests opportunities for future funding programmes.

Europe tends to be more self-reliant in the thin-film technologies, with CdTe cells in the range of 15–23% efficiency, similar to organic-based cells. However, these materials present other challenges, such as cadmium being registered under REACH regulation due to its toxicity^35^, while organic photovoltaics face efficiency limitations associated with hopping transport mechanisms that may hinder large-scale investment. GaAs, as the record holder for single-junction solar cells, scores high on efficiency but low on self-reliance owing to 69% of Europe’s gallium being supplied by China. While dye-sensitised solar cells have begun to reach appreciable efficiencies, especially with co-sensitised cells, their reliance on TiO_2_ nanoparticles reduces their self-reliance scores. As the most nascent technology, ferroelectric solar cells have efficiencies <10% but are already competitive with iodine-free perovskites and cuprous oxide (Cu_2_O) thin films.

## Discussion

The self-reliance index developed here bridges the established field of raw material criticality into the domain of advanced materials. Just as advanced materials are synthesised from raw materials, the self-reliance index is built from components of raw material criticality assessments, like the import reliance, recycling rates, and supplier concentration^13,17,18^, but repurposes them into a forward-looking selection tool that allows performance to be linked to local availability. Beyond its primary function of identifying European-sourced materials, the index’s components offer additional strategic insights: the graphical data in Figs. 4–6 readily highlight potential material substitutes, while the critical import fraction pinpoints candidates where improved recycling input rates will yield the greatest benefit. Furthermore, the methodological framework is highly scalable. Combining a simple, comprehensive metric with the geometric mean allows the concept of self-reliance to be transmitted from elements to compounds to entire functional devices.

**Fig. 7:**
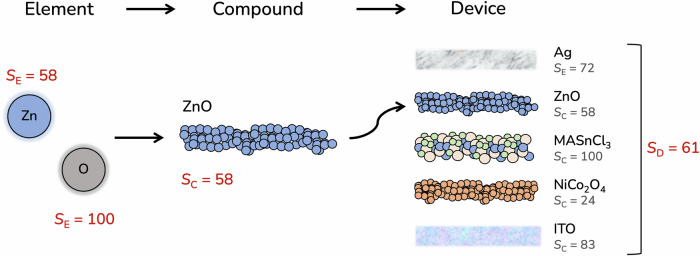
Propagation of self-reliance. An example where the elemental self-reliance, *S*_E_, of zinc and oxygen combine into a compound self-reliance, *S*_C_, as ZnO nanoparticles. A thin film of ZnO can be used as the electron transport layer in a perovskite photovoltaic solar cell, where the self-reliance indexes of the other components can also be computed into a device-level self-reliance, *S*_D_.

This scalability is demonstrated in Fig. 7, which models a perovskite solar cell. Starting from the elemental level, zinc and oxygen have elemental self-reliance indexes of 58 and 100, respectively. These are then synthesised into zinc oxide nanoparticles, with a compound self-reliance of *S*_C_ = 58 (noting that oxygen is ubiquitous and treated neutrally in compound calculations). A thin film of zinc oxide nanoparticles can then be used as the electron transport layer in the solar cell. Taking the geometric mean of each component’s self-reliance index gives a device self-reliance index, *S*_D_ = (72 × 58 × 100 × 24 × 83)^1/5^ = 61. This cascading calculation visually demonstrates how elemental self-reliance propagates through a system and pinpoints specific components, such as nickel cobaltite (NiCo_2_O_4_) as the hole extraction layer, that represent vulnerabilities and prime targets for substitution.

Looking more granularly, the route from an element to a compound can vary and may influence how self-reliance is transmitted along the chain. In physical synthesis routes, such as vapour-phase transport, the propagation of self-reliance is straightforward: purified elements are directly combined to form compounds, as with molybdenum and selenium to create molybdenum diselenide MoSe_2_. However, chemical routes often involve intermediate precursor steps, such as acetates, nitrates, or chlorides. For example, processing as-mined sphalerite (ZnS) into zinc nanoparticles can involve an intermediary step where the zinc oxide (ZnO) is converted to zinc acetate hydrate. Similarly, the synthesis of In_2_O_3_ may require indium acetylacetonate^36^, SnO_2_ can derive from tin (II) chloride (SnCl_2_)^37^, and cobalt tetroxide (Co_3_O_4_) from cobalt(II) nitrate (Co(NO_3_)_2_)^38^. However, the weakest link in these pathways typically lies in the metal precursor itself rather than in the organic or anionic intermediates, which are mostly composed of ubiquitous elements and readily available. Therefore, in this work, we treat the transmission of self-reliance as following the geometric mean of the constituent elements within each compound, independent of the synthesis route.

The goal of this work is to demonstrate how criticality concepts can be applied to advanced materials. To do so, key distinctions from the framework for raw materials must be established. For raw materials, ‘criticality’ combines high economic importance with high supply risk, with a ‘strategic’ subset denoting those essential for green, digital, defence, and space technologies^19,39^. This assessment reflects present-day economic and geopolitical realities. Advanced materials, however, differ in the basis of their importance as it derives not from the economic size of existing sectors, but from their unique properties that can drive technological breakthroughs. Innovations in wearable electronics, novel solar cells, or high-capacity batteries emerge at low technology readiness levels, but incentivised commercialisation can make them indispensable for next-generation technologies. Consequently, a potentially important advanced material can be identified by its state-of-the-art performance relative to other materials used for a given technology. If it outperforms other materials on average, it is likely to be worth targeting for development.

A second distinction concerns substitutability. For bulk raw materials, substitution is assessed at the elemental level and focuses on the feasibility of replacing one element with another as a means of mitigating supply shocks. For advanced materials, however, substitution has two distinct but related meanings. First, it can refer to the choice of component elements during synthesis, where replacing one precursor element with a chemically similar analogue yields a different, but functionally related, material; e.g., substituting tungsten (*S*_E_ = 75) for molybdenum (*S*_E_ = 14) during vapour-phase synthesis to produce crystals of WS_2_ instead of MoS_2_. Second, it can refer to device-level substitution, where one compound can replace another in a given application without significant loss of performance; for example, platinum diselenide (PtSe_2_) (*S*_C_ = 29) could be swapped for indium (III) oxide (In_2_O_3_) (*S*_C_ = 75) in a transistor while maintaining roughly the same charge-carrier mobility, assuming compatibility with device architecture and fabrication routes. Although a methodology exists to quantify substitutability for raw materials, it is currently unclear how to consolidate factors such as chemical compatibility, processing requirements, device architectures, and material stability into a single measure for advanced materials.

These distinctions affect how supply risk should be interpreted for advanced materials. As their importance lies in their future breakthrough potential, and as constituent elements can often be exchanged without significant loss of performance, advanced materials offer an opportunity not readily available for present-day technologies based on critical materials. Instead of using reactive indicators tied to contemporary supply vulnerabilities, supply security for next-generation technologies can be proactively designed into the materials themselves by favouring compositions based on locally available materials. Because advanced materials are governed by these distinct mechanisms of importance and supply risk, we propose that they merit treatment as a separate strategic subset within the broader criticality framework.

To demonstrate how designating an advanced material as ‘strategic’ would work in practice, we apply two quantitative thresholds to the nanomaterials in this work. First, we define “high technological performance” as exceeding a critical performance threshold. This threshold is set at the mean of all values within a given performance indicator; for example, the mean mobility, <*μ* > , across all materials in Fig. 4c is 41 cm^2 ^V^−1^ s^−1^, which we designate as the critical mobility, *μ*_C_. This is how the lower boundary of the blue regions in Figs. 4–6 is set.

We believe the mean is a neutral and consistent threshold for comparing diverse material systems that provides an objective, first-order indicator of relative performance. Such an approach answers a fundamental strategic question: does a material perform above the current average for its technology? If so, it merits further development as a promising candidate, after which second-order factors such as ambient stability or cost can be considered. Importantly, this critical performance threshold is not static. Each improvement in the state of the art raises the strategic bar, thereby rewarding materials that advance leading performance and penalising those that fail to keep pace with technological progress.

Second, we define a self-reliance threshold using a critical import fraction and dominant supplier fraction of 0.3, to indicate moderate risk exposure, which results in a self-reliance index of 57. For consistency with Figs. 2a and 3, we round this to 60. This threshold then forms the left-hand boundary of each blue region in Figs. 4–6. Materials that lie above both thresholds (within the blue regions) we designate as ‘strategic,’ which provides a straightforward method for prioritising local materials with the potential for use in next-generation technologies. Supplementary Note 3 lists the strategic nanomaterials from Figs. 4–6.

Applying this methodology to the theoretical mobilities in Supplementary Note 2 also demonstrates how the self-reliance index can support predictive tools. High-throughput computational screening (e.g., via the Materials Cloud 2D Database) can identify new materials, density functional theory can predict their performance, and the self-reliance index can determine which are locally available. As shown in Supplementary Fig. 4, these steps narrow 336 semiconductors down to 28 that can then be prioritised for experimental development according to second-order factors.

The designation of certain advanced materials as strategic moves the focus away from vulnerable global supply chains and toward the question of how local materials can be synthesised into high-performance compounds that match or exceed the performance of imported alternatives. A strategic classification for advanced materials provides a useful method of down-selection that could align sectors of research, industry, and policy toward greater European technological resilience. For researchers, it prioritises the exploration and development of high-performance, locally sourced materials that may have been overlooked in pursuit of state-of-the-art. For industry, it incentivises the design of next-generation devices that prioritise domestic materials, thereby mitigating exposure to volatile global supply chains. For policymakers, it offers a versatile framework that is compatible with the goals of funding programmes to reduce over-reliance on non-European sources.

While this definition creates a practical precedent, the present work has limitations. Our use of import reliance as a proxy for availability, while essential for a consistent cross-element comparison, masks a critical nuance: a 100% import reliance can signify either a complete absence of domestic extraction (e.g., zirconium) or domestic production that fails to meet total cross-sectoral demand (e.g., titanium). In the first scenario, it is clear that a nanomaterial should substitute this element with a locally available alternative. In the second, the comparatively small material volumes required for nanomaterial synthesis may remain accessible even during a broad supply crisis. However, this access is precarious as it depends on the availability of material at the 5 N purities required for synthesis while also being exposed to the price volatility of the imported raw material, as seen with the two to threefold price swings of titanium, aluminium, and iron ore in recent years^40^, the recent run on gold and silver prices, and fluctuating energy costs of high-level purification. Furthermore, a comprehensive nanomaterial evaluation must eventually integrate factors beyond performance and supply, such as ambient stability, toxicity, recyclability, and compatibility with scalable manufacturing. Similarly, quantifying how substitutable an element is will require chemistry and synthesis considerations. The incorporation of these dimensions will form the basis of our future work.

This study focuses on how nanomaterial self-reliance relates to energy electronics, but it can be easily applied to other advanced material subsets or product pathways. In the domain of healthcare, silver, sourced from Poland or Sweden, can be refined into precursors for nanoparticle synthesis. Silver nanoparticles are then incorporated into antimicrobial biomaterial coatings used in wound dressings, medical textiles, and analytical devices. Titanium is widely used in the construction and infrastructure industries, and is highly abundant but heavily imported as its purified metallic form is needed to create a range of TiO_2_ nanostructures. These can then be incorporated into photocatalytic or self-cleaning coatings for glass and cement (the ‘smart material’ subset of advanced materials). Zinc is produced all across Europe and, when processed into various ZnO nanostructures, finds applications in antimicrobial coatings for food packaging and as UV-protective or antifungal additives in crop protection products. Our future work will focus on extending this analysis to the other categories of advanced materials, such as biomaterials, ceramic materials, smart and functional materials, hybrid materials, and advanced metallic materials.

In mapping a self-reliance index across a broad range of nanomaterials, this work provides a quantitative basis for selecting materials that balance technological performance with resilience to geopolitical, trade, and price instabilities. Importantly, as the index is derived from the European Commission’s periodic criticality assessments, it can be continuously updated with each new report on critical raw materials, typically issued every three years, and recalibrated against contemporary state-of-the-art performance. Taken together, this highlights a growing reality: high performance in advanced technologies will no longer be paid for solely in cost but also in certainty of access.

## Methods

### Self-reliance index calculations

The elemental self-reliance index values, *S*_E_, were calculated using Eq. (2) and compound self-reliance index values, *S*_C_, with Eq. (3) in the main text, both using the values in Supplementary Table 2. As organic materials are non-stoichiometric, we take each element that is present in the compound and assign it a stoichiometric value of 1 and calculate *S*_C_ as normal. For example, PM6:BTP-eC9:BTP-S16:BTP-S17 is used as the absorption layer in organic photovoltaic solar cells and is composed of the elements C, H, S, N, O, F, and Cl. We therefore calculate *S*_C_ ${=\left(100\times 29\right)}^{1/2}=54$, noting that C, H, N, O, and Cl are ubiquitous elements and are treated neutrally in the calculation.

### Methodology to select strategic nanomaterials

Step 1: A strategic nanomaterial is a material with at least one dimension below 100 nm, often a compound or composite, whose nanoscale properties make it essential to the green transition, digital infrastructure, defence capabilities, and space systems. Their functionality differs fundamentally from that of bulk materials, as performance is determined not only by chemical composition but by size-dependent physical and chemical phenomena. Strategic nanomaterials can therefore be distinguished from critical/strategic raw materials by three defining characteristics: (1) they exhibit size-dependent electronic, optical, catalytic, or mechanical properties that do not occur in the corresponding bulk material and that directly determine technological performance; (2) they advance the state-of-the-art in strategic technologies, delivering performance levels such as enhanced efficiency, durability, sensitivity, or energy conversion that cannot be achieved using bulk alternatives; and (3) they often belong to broad material families in which compounds share similar crystal structures and nanoscale behaviour, allowing substitution of high-risk or heavily imported elements with more locally available analogues without sacrificing performance (for example, substitution within transition-metal dichalcogenides or among nano-catalyst compositions)

Step 2: The performance threshold for determining which materials shall be considered strategic is calculated as follows: (1) for each technology, a single, technologically relevant performance indicator is selected (e.g., theoretical capacity for battery electrodes); (2) for every nanomaterial used in that technology, the highest peer-reviewed state-of-the-art value reported for the selected performance indicator is identified; and (3) a critical performance threshold is then calculated from all identified state-of-the-art values, using the geometric mean when the ratio between the highest and lowest values exceeds one order of magnitude, and the arithmetic mean when this ratio is below a factor of ten

Step 3: The self-reliance index threshold for determining which materials shall be considered strategic is selected according to the desired risk tolerance regarding import dependence and supplier concentration, and is calculated using Eq. (2) in the main text. For the present analysis, we adopt a threshold value of 0.3, representing a conservative level of exposure to external supply risk. Under this criterion: *IR*_C_ ≤ 0.3, *f* ≤ 0.3, producing *S*_i_ ≥ 57, where *i* = *E*, *C*, or *D* denotes the self-reliance index calculated for elements, compounds, or devices, respectively.

Step 4: Nanomaterials that surpass both the performance threshold and the self-reliance index threshold for a given technology shall be considered ‘strategic’ for that technology.

### Semiconductor selection process

To create a list of semiconducting 2D materials to search for, we extracted the 781 “easily exfoliable” materials from the Supplementary Information of the Materials Cloud 2D Database^41^ and sorted them by bandgap, classifying a semiconductor as having an *E*_g_ < 2.5 eV. This gave 336 materials for which we performed a literature review seeking the state-of-the-art charge-carrier mobility for each material. We sought both experimentally measured and theoretical mobilities for all materials, with the full lists of materials for which we found values in Supplementary Tables 5–7. The mobility values in Fig. 4d, 4d represent the highest values we could find for experimental measurements, but note that those we found may not necessarily be the highest reported values and, as such, can be interpreted as a sufficiently high value for the purposes of this analysis.

### Battery electrode selection process

For each battery type, we sought recent reviews that mention theoretical capacities for a range of both inorganic and organic electrodes. For inorganic electrodes, we sought values that were sufficiently different from each other to be visible on the graphs in Fig. 5. For organic electrodes, we started with the highest and lowest theoretical capacities we could find, and then included a selection of other materials within this range. The full list of materials is available in Supplementary Tables 8–10.

### Photovoltaic material selection process

The data in Fig. 6b–d were sourced from the Perovskite Database Project^42^, which compiles performance metrics of perovskite solar cells from publications dating back to 2013. For this analysis, we selected publications from 2015 to 2025, totalling approximately 43,000 entries. For each layer, we selected the best-performing materials for analysis and presentation in Fig. 6. The data for the other thin-film PV technologies in Fig. 6d are based on the highest efficiency devices reported to date. The *S*_C_ values for the DSSCs in Fig. 6d were calculated using the composition of the dye plus the TiO_2_ nanoparticles. The full list of materials is available in Supplementary Tables 11–15.

## Supplementary information

Supplementary Information

Transparent Peer Review file

## Data Availability

All data are available in the Supplementary Information.
