# Supplementary material for: A self-reliance framework for identifying strategic advanced materials
Source: Nat Commun. 2026 May 21;17:6696. doi: 10.1038/s41467-026-73294-8 (PMC13385709; doi:10.1038/s41467-026-73294-8)
Supplement: Supplementary file 1 — Supplementary Information [file 41467_2026_73294_MOESM1_ESM.pdf]

**Supplementary Information for**

# A self-reliance framework for identifying strategic advanced materials

Cristina Teixeira,[1] Cian Gabbett,[2] Kevin Synnatchske,[3] Jonathan N. Coleman,[2] Zdenek Sofer,[4] Manuel J. Mendes,[1] Elvira Fortunato,[1] Rodrigo Martins,[1] Luis Pereira,[1] & Adam G. Kelly[1,∀]

[1]CENIMAT/i3N, Department of Material Science, NOVA School of Science and Technology, Campus de Caparica, 2829-516, Caparica, Portugal

[2]School of Physics, CRANN & AMBER Research Centres, Trinity College Dublin, Dublin 2, Ireland

[3]Center for Advancing Electronics Dresden (CfAED) and Faculty of Chemistry and Food Chemistry, Technische Universität Dresden, 01062 Dresden, Germany

[4]Department of Inorganic Chemistry, University of Chemistry and Technology Prague, Technická 5, Prague 6 166 28, Czech Republic.

[∀]Corresponding author: a.kelly@fct.unl.pt

## Table of contents


| | | |
|---|---|---|
| **Supplementary Table 1** | Elements and minerals…… | 3 |
| **Supplementary Table 2** | Abundance, suppliers, and self-reliance…… | 8 |
| **Supplementary Figure 1** | Periodic table (Abundance redux)…… | 10 |
| **Supplementary Figure 2** | Environmental performance index…… | 11 |
| **Supplementary Note 1** | Aggregating self-reliance index scores…… | 12 |
| **Supplementary Figure 3** | The effect of oxygen on compound calculations…… | 13 |
| **Supplementary Table 3** | Conductive materials…… | 15 |
| **Supplementary Table 4** | Dielectric materials…… | 16 |
| **Supplementary Table 5** | Semiconducting materials: Single crystals…… | 17 |
| **Supplementary Table 6** | Semiconducting materials: Thin films…… | 19 |
| **Supplementary Note 2** | Semiconducting materials: Theoretical mobilities…… | 20 |
| **Supplementary Figure 4** | Theoretical mobilities for single crystal 2D materials…… | 20 |
| **Supplementary Table 7** | Theoretical mobilities for single crystal 2D materials…… | 21 |
| **Supplementary Table 8** | Batteries: Lithium-ion electrodes…… | 24 |
| **Supplementary Table 9** | Batteries: Sodium-ion electrodes…… | 26 |
| **Supplementary Table 10** | Batteries: Potassium-ion electrodes…… | 27 |
| **Supplementary Table 11** | Photovoltaics: Hole transport materials (p-i-n configuration)…… | 28 |
| **Supplementary Table 12** | Photovoltaics: Hole transport materials (n-i-p configuration)…… | 29 |
| **Supplementary Table 13** | Photovoltaics: Electron transport materials (p-i-n configuration)…… | 30 |
| **Supplementary Table 14** | Photovoltaics: Electron transport materials (n-i-p configuration)…… | 31 |
| **Supplementary Table 15** | Photovoltaics: Absorption layers…… | 32 |
| **Supplementary Note 3** | List of strategic nanomaterials…… | 34 |
| **Supplementary References** | | 36 |

**Supplementary Table 1** | Elements and minerals

| Element | | Primary Mineralisation Type | Primary Mineral(s) | Cost (€/kg) | Purity |
|---|---|---|---|---|---|
| 1 | H | Fossil fuel processing<br>Water electrolysis | – | 3.5–5 | >99.99% |
| 2 | He | By-product of natural gas extraction | – | 109 | >99.999% |
| 3 | Li | Li-pegmatites<br>Brine<br>Evaporites<br>Greisen-type deposits | Spodumene, lepidolite | 75–90 | >99.9% |
| 4 | Be | Pegmatite<br>Greisen | Beryl | 904 | Unstated |
| 5 | B | Evaporites<br>Geothermal/hydrothermal deposits | Borax, ulexite, colemanite | 82.75 | Unstated |
| 6 | C | Metamorphosed sedimentary rock<br>Hydrothermal and metasomatic deposits | Graphite, diamond | 0.5–5 | >99% |
| 7 | N | Atmospheric distillation | – | 0.1–2 | >99.99% |
| 8 | O | Atmospheric distillation | – | 0.1–2 | >99.99% |
| 9 | F | Hydrothermal deposits<br>Sedimentary deposits | Fluorite | 0.4–1<br>($CaF_2$) | Unstated |
| 10 | Ne | Atmospheric distillation | – | – | 99.994% |
| 11 | Na | Evaporites<br>Brine | Halite | 1.8–2.2 | >99.5% |
| 12 | Mg | Evaporite deposits (chlorides)<br>Sedimentary rocks (magnesite, dolomite) | Magnesite, dolomite | 2–3 | >99.9% |
| 13 | Al | Lateritic deposits | Bauxite (oxo-hydroxides and hydroxides of aluminium formed by weathering of Al silicates and oxides.) | 2–3 | >99% |
| 14 | Si | Hydrothermal deposits<br>Metamorphic deposits<br>Sedimentary deposits | Quartz, feldspar | 0.1–1.5 | >99% |
| 15 | P | Sedimentary deposits (phosphorites)<br>Magmatic and skarn deposits (apatite)<br>Marine sedimentary deposits | Pyromorphite, apatite | 3–3.5<br>(Yellow P) | >99.9% |
| 16 | S | Oil processing<br>Pyrite deposits<br>Evaporites<br>Hydrothermal deposits | Native sulphur, pyrite, gypsum | 2–2.5 | >99% |
| 17 | Cl | Evaporites<br>Brines | Salt, sylvite, carnalite | 0.3–0.7 | >99% |
| 18 | Ar | Atmospheric | – | 27 | >99.999% |
| 19 | K | Potash (Potassium chlorides)<br>Evaporites<br>Brine solutions | Sylvite, carnallite | 20–25 | >98% |
| 20 | Ca | Sedimentary deposits (limestone, gypsum)<br>Brine<br>Evaporites | Calcite, gypsum, dolomite | 3–5 | >98.5% |
| 21 | Sc | Laterite (By-product of bauxite processing)<br>Hydrothermal deposits (W-Sn)<br>Various types of RE deposits | Thortveitite, kolbeckite<br>(High variability across deposits) | 3200–3800 | >99.9% |
| 22 | Ti | Sedimentary deposits<br>Magmatic deposits<br>Metamorphic and volcano-sedimentary deposits | Ilmenite, rutile<br>(High variability across deposits) | 5–6 | >99%<br>(Sponge) |
| 23 | V | Uranium-rich sandstone processing<br>Sedimentary deposits (U)<br>Magmatic deposits (Fe–Ti)<br>By-product of oil, phosphate, and bauxite processing | Vanadinite, patronite | 170–190 | >99.5% |
| 24 | Cr | Magmatic and sedimentary deposits | Chromite | 8 | 99% |

| Element | | Primary Mineralisation Type | Primary Mineral(s) | Cost (€/kg) | Purity |
|---|---|---|---|---|---|
| | | Ultramafic | | | |
| 25 | Mn | Residual, sedimentary, and volcano–sedimentary deposits<br>Skarn and hydrothermal deposits<br>Marine nodules | Pyrolusite, psilomelane, cryptomelane | 1.5–2 | >99.5% |
| 26 | Fe | Sedimentary, metamorphic, Magmatic, and hydrothermal deposits<br>Skarn<br>Carbonatite<br>Laterite | Hematite, magnetite<br>(Highly abundant but high variability across deposits) | 0.5–1 | >99.9% |
| 27 | Co | Hydrothermal deposits (Ni–Co–As, U-Ag-Bi-Co-Ni)<br>Stratiform deposits<br>Skarn deposits<br>Magmatic deposits<br>By-product of Cu and Ni mining | Cobaltite, safflorite skutterudite | 25–35 | >99% |
| 28 | Ni | Magmatic deposits (Ni–Cu)<br>Lateritic deposits<br>Hydrothermal deposits (Ni-Co-As, Ag-U-Bi-Co-Ni) | Pentlandite, garnierite, nickeline | 15–20 | >99% |
| 29 | Cu | Hydrothermal polymetallic deposits (Zn-Pb-Cu, Cu-Fe)<br>Porphyry deposits (Cu, Cu-Mo)<br>Proterozoic deposits (Cu-Co)<br>Magmatic and volcanogenic deposits | Chalcopyrite, bornite | 4–5 | >99.9% |
| 30 | Zn | Hydrothermal deposits (Pb-Zn)<br>Telethermal deposits (Pb-Zn, Pb-Zn-Cu)<br>Volcano-sedimentary deposits (Pb-Zn, Pb-Zn-Cu)<br>Skarn-type deposits | Sphalerite, wurtzite, smithsonite | 2–3 | >99.9%<br>(Ingot) |
| 31 | Ga | By-product of bauxite/Zn ores<br>Hydrothermal deposits (Pb–Zn)<br>Telethermal deposits (Pb–Zn, Pb–Zn–Cu)<br>Coal-ash processing | Gallite, tsumgallite | 250–300 | >99.99% |
| 32 | Ge | By-product of Zn ores (sphalerite)<br>Hydrothermal deposits (Pb–Zn)<br>Telethermal deposits (Pb–Zn, Pb–Zn–Cu)<br>Coal-ash processing | Germanite, argyrodite, renierite | 2500–3000 | >99.99% |
| 33 | As | By-product of polymetallic ore processing including Au and Ag production<br>Hydrothermal polymetallic deposits (Ag–Au, Ag–Sn–W–Bi, U–Ag–Bi–Co–Ni, Bi–As)<br>Subvolcanic hydrothermal As ores | Realgar, auripigmet, arsenopyrite, lollingite, orpiment | 0.9–1.5<br><br>45–55 | >99.9%<br><br>99.9999% |
| 34 | Se | By-product from Cu refining<br>Hydrothermal deposits (Pb–Zn–Cu, Au–Ag) | Berzelianite, umangite, clausthalite | 25–30 | >99.9%<br>(Ingot) |
| 35 | Br | Brines<br>Evaporites | Bromargyrite, kuzminite | 3.1 | – |
| 36 | Kr | Atmospheric distillation | – | – | – |
| 37 | Rb | By-product of Li- and Cs-rich mica<br>Pegmatites deposits<br>Evaporite and brine processing | No separate mineral species | 13–25 k | >99.9% |
| 38 | Sr | Alkaline, sienite and carbonatite deposits<br>Sedimentary sulphur deposits | Celestine, witherite | 6–10 | >99.9% |
| 39 | Y | Sedimentary deposits<br>Carbonatite deposits<br>Metasomatite and hydrothermal deposits<br>Co-product with Ta, Nb, Th, and rare earths | Xenotime, Euxenite, Samarskite<br>(High variability across deposits) | 28–35 | >99.9% |
| 40 | Zr | By-product of phosphate mining<br>Sedimentary and hard-rock deposits<br>Placer deposits | Zircon, baddeleyite | 20–25 | >99%<br>(Sponge) |
| 41 | Nb | Sedimentary, magmatic and metamorphogenic deposits<br>Pegmatite | Pyrochlore, columbite, microlite | 80–100 | >99.9% |

| Element | | Primary Mineralisation Type | Primary Mineral(s) | Cost (€/kg) | Purity |
|---|---|---|---|---|---|
| | | Carbonatite<br>Co-product with Ta, Th, and rare earths | | | |
| 42 | Mo | Porphry deposits (Mo, Cu–Mo)<br>Hydrothermal deposits (Sn–W)<br>Skarn and greisen deposits | Molybdenite, wulfenite, powellite, ferrimolybdite | 50–65 | >99.9% |
| 43 | Tc | Synthetic | – | – | – |
| 44 | Ru | By-product of Au and Cu processing<br>Magmatic (Ni–Cu, chromite)<br>Liquation sulphide deposits<br>Sedimentary deposits | Laurite, ruthenium, ruarsite | 13–25 k | >99.9% |
| 45 | Rh | By-product of Au and Cu processing<br>Magmatic (Ni–Cu, chromite)<br>Liquation sulphide deposits<br>Sedimentary deposits | Bowleite, hollingworthite | 160–200 k | >99.9% |
| 46 | Pd | By-product of Au and Cu processing<br>Magmatic (Ni–Cu, chromite)<br>Liquation sulphide deposits<br>Sedimentary deposits<br>Au hydrothermal deposits | Native Pd, merenskyite, vysotskite | 28–34 k | >99.9% |
| 47 | Ag | By-product of Zn, Pb and Cu mining<br>Polymetallic hydrothermal deposits (Pb-Zn, Pb-Zn-Cu, Ag-Au, Ag-Sn-W-Bi, U-Ag-Bi-Co-Ni) | Native silver, acanthite, argentite, proustite, polybasite | 900–1000 | >99.9% |
| 48 | Cd | By-product of sphalerite<br>Hydrothermal polymetallic deposits (Pb-Zn-Cu) | Greenockite, Otavite, Cd-wurtzite | 5 | 99.99% |
| 49 | In | By-product of Zn and Sn ores<br>Hydrothermal deposits (Pb–Zn, Sn–W)<br>Telethermal deposits (Pb–Zn, Pb–Zn–Cu) | Roquesite, ramdohrite, dzhalindite | 330–380 | >99.99% |
| 50 | Sn | Hydrothermal deposits (Sn-W, Sn-Ag-Bi-W, Sn-W-Bi)<br>Subvolcanic, Greisen, and pegmatite deposits<br>Co-product with W, Nb–Ta, and rare earths | Cassiterite, stannite, kesterite<br>(High variability across deposits) | 30–35 | >99.9%<br>Ingot |
| 51 | Sb | By-product of hydrothermal deposits (including gold production)<br>Telethermal deposits (Sb-Hg, Sb)<br>Hydrothermal deposits (Pb-Zn, Au) | Antimonite, antimony, pyrargyrite | 25–30 | >99% |
| 52 | Te | By-product of Cu refining and Au–Ag ores<br>Hydrothermal deposits (Pb-Zn-Cu, Au-Ag) | Tetradymite, tellurium, hessite | 80–100 | >99.9% |
| 53 | I | Brines associated with gas and oil deposits<br>Evaporites | No separate mineral species | 65–80 | >99.9% |
| 54 | Xe | Atmospheric distillation | – | – | – |
| 55 | Cs | Pegmatite deposits<br>Sn–W deposits<br>By-products of lithium mining | Pollucite, nanpingite, galkhaite | 68–72 k | >99.99% |
| 56 | Ba | Hydrothermal and bedded volcanic deposits (pure baryte and with polymetallic ores)<br>Co-product with fluorite and polymetallic ores | Baryte, witherite | 0.3–1<br>($BaSO_4$) | >99% |
| 57 | La | Sedimentary deposits<br>Carbonatite deposits<br>Metasomatite and hydrothermal deposits<br>Co-product with Nb, Ta, Th, and other rare earths | Monazite, bastnäsite<br>(High variability across deposits) | 2.5–3.5 | >99% |
| 58 | Ce | Sedimentary deposits<br>Carbonatite deposits<br>Metasomatite and hydrothermal deposits<br>Co-product with Nb, Ta, Th, and other rare earths | Monazite, bastnäsite, allanite<br>(High variability across deposits) | 3.5–4 | >99% |
| 59 | Pr | Sedimentary deposits<br>Carbonatite deposits<br>Metasomatite and hydrothermal deposits<br>Co-product with Nb, Ta, Th, and other rare earths | No separate mineral species<br>(High variability across deposits) | 75–100 | >99% |
| 60 | Nd | Sedimentary deposits | Monazite, bastnäsite | 50–60 | >99.9% |

| Element | | Primary Mineralisation Type | Primary Mineral(s) | Cost (€/kg) | Purity |
|---|---|---|---|---|---|
| | | Carbonatite deposits<br>Metasomatite and hydrothermal deposits<br>Co-product with Nb, Ta, Th, and other rare earths | (High variability across deposits) | | |
| 61 | Pm | Synthetic | – | – | – |
| 62 | Sm | Sedimentary deposits<br>Carbonatite deposits<br>Metasomatite and hydrothermal deposits<br>Co-product with Nb, Ta, Th, and other rare earths | Monazite, allanite<br>(High variability across deposits) | 10–15 | >99.9% |
| 63 | Eu | Sedimentary deposits<br>Carbonatite deposits<br>Metasomatite and hydrothermal deposits<br>Co-product with Nb, Ta, Th, and other rare earths | No separate mineral species<br>(High variability across deposits) | 600–1000 | >99.9% |
| 64 | Gd | Sedimentary deposits<br>Carbonatite deposits<br>Metasomatite and hydrothermal deposits<br>Co-product with Nb, Ta, Th, and other rare earths | Monazite, xenotime<br>(High variability across deposits) | 80–160 | >99.9% |
| 65 | Tb | Sedimentary deposits<br>Carbonatite deposits<br>Metasomatite and hydrothermal deposits<br>Co-product with Nb, Ta, Th, and other rare earths | No separate mineral species<br>(High variability across deposits) | 950–1200 | >99.9% |
| 66 | Dy | Sedimentary deposits<br>Carbonatite deposits<br>Metasomatite and hydrothermal deposits<br>Co-product with Nb, Ta, Th, and other rare earths | No separate mineral species<br>(High variability across deposits) | 220–300 | >99.9% |
| 67 | Ho | Sedimentary deposits<br>Carbonatite deposits<br>Metasomatite and hydrothermal deposits<br>Co-product with Nb, Ta, Th, and other rare earths | No separate mineral species<br>(High variability across deposits) | 250–500 | >99.9% |
| 68 | Er | Sedimentary deposits<br>Carbonatite deposits<br>Metasomatite and hydrothermal deposits<br>Co-product with Nb, Ta, Th, and other rare earths | No separate mineral species<br>(High variability across deposits) | 100-250 | >99.9% |
| 69 | Tm | Sedimentary deposits<br>Carbonatite deposits<br>Metasomatite and hydrothermal deposits<br>Co-product with Nb, Ta, Th, and other rare earths | No separate mineral species<br>(High variability across deposits) | 800–1200 | >99.9% |
| 70 | Yb | Sedimentary deposits<br>Carbonatite deposits<br>Metasomatite and hydrothermal deposits<br>Co-product with Nb, Ta, Th, and other rare earths | Xenotime, samarskite<br>(High variability across deposits) | 30–50 | >99.5% |
| 71 | Lu | Sedimentary deposits<br>Carbonatite deposits<br>Metasomatite and hydrothermal deposits<br>Co-product with Nb, Ta, Th, and other rare earths | No separate mineral species<br>(High variability across deposits) | 520–560 | >99.99% |
| 72 | Hf | By-product of zirconium<br>Sedimentary and hard-rock deposits<br>Placer deposits | No separate mineral species | 900–1400 | >99.9% |
| 73 | Ta | Co-product of Nb, Zr, Th, and rare earths<br>Sedimentary deposits<br>Pegmatite and carbonatite deposits<br>Various magmatic and metamorphic deposits. | Tantalite, tapiolite<br>(High variability across deposits) | 320–350 | >99.9% |
| 74 | W | Skarn deposits (W)<br>Hydrothermal deposits (Sn–W)<br>Stratiform deposits (Scheelite)<br>Greisen deposits (Sn–W) | Ferberite, wolframite, scheelite, hubnerite<br>(High variability across deposits) | 45–55 | >99.9%<br>(Bar) |
| 75 | Re | By-product from Mo and Cu ores<br>Porphyry (Mo, Cu–Mo) deposits<br>Hydrothermal deposits (Sn–W)<br>Skarn (Mo)- and greisen (Mo–W)-type deposits | Rhenite | 2400–3000 | >99.99% |
| 76 | Os | Magmatic (Ni–Cu, chromite) | Osmiridium, erlichmanite | 150–250 k | >99.9% |

| Element | | Primary Mineralisation Type | Primary Mineral(s) | Cost (€/kg) | Purity |
|---|---|---|---|---|---|
| | | Liquation sulphide deposits<br>Sedimentary deposits<br>By-product of Pd-Pt processing | | | |
| 77 | Ir | Magmatic (Ni–Cu, chromite)<br>Liquation sulphide deposits<br>Sedimentary deposits<br>By-product of Pd-Pt processing | Iridium, irarsite, rutheniridosmine | 90–120 k | >99.9% |
| 78 | Pt | Magmatic (Ni–Cu, chromite)<br>Liquation sulphide deposits<br>Sedimentary deposits<br>By-product of Au and Cu processing | Platinum, sperrylite | 28–32 k | >99.9% |
| 79 | Au | Hydrothermal polymetallic deposits<br>(Au, Au-Te, Au-Ag, Pb-Zn-Cu, Cu-Mo)<br>Sedimentary deposits<br>By-product of polymetallic ore processing | Native gold, sylvanite, petzite, calaverite | 85–110 k | >99.9% |
| 80 | Hg | Epithermal deposits<br>Volcano-sedimentary deposits | Cinnabar, corderoite, livingstonite | – | – |
| 81 | Tl | Telethermal Pb-Zn deposits, As-Sb deposits<br>By-product from base metal ores | Lorandite, crooksite | 300–1000 | >99.9% |
| 82 | Pb | Hydrothermal deposits (Pb-Zn, Pb-Zn-Cu)<br>Skarn, telethermal, volcano-sedimentary (Cu-Zn-Pb) deposits | Galena, cerussite, anglesite | 2–3 | >99.9% |
| 83 | Bi | By-product of Cu and Cu-Mo porphyry ores<br>Hydrothermal polymetallic deposits<br>(Pb-Zn-Cu, Au-Ag-Bi, Sn-W, U-Ag-Bi-Co-Ni, Bi-As) | Native bismuth, bismuthinite, tetradymite, cosalite | 12–15 | >99.99% |

**Supplementary table 1 | Elements, minerals and cost.** List of elements from hydrogen to bismuth showing their primary mineralisation type, primary mineral, cost and purity. Co-product refers to deliberately targeted elements during the mining process, often requiring dedicated extraction or refining steps. For example, a mine producing both copper and gold from the same ore, where both metals are economically vital and necessitate planned processing. By-product refers to elements that are recovered incidentally during the main extraction process and are not the primary focus of operations. For example, silver is often recovered during lead–zinc mining, where silver is a secondary output and not central to the mine's focus. The mineralisation data were obtained from Refs.[1, 2, 3]. The costs were obtained from Refs.[4, 5, 6, 7, 8]. Where possible, prices were averaged over a month. Prices in dollars were converted to Euro using the USD/EUR rate on 13th April 2025 (€0.880413).

**Supplementary Table 2** | Abundance, suppliers, and self-reliance

| Element | | Abundance (ppm)[ϒ] | *IR* (%) | *R* (%) | *C* (%)[ς] | $S_E$ | Largest EU Supplier | EU deposits[∀] |
|---|---|---|---|---|---|---|---|---|
| 1 | H | 1400 | 0 | – | – | 100 | Widespread | |
| 2 | He | 0.008 | 94 | – | – | 3 | US (56%) | |
| 3 | Li | 20 | 100 | 0 | 0 | 0 | Chile (79%) | |
| 4 | Be | 2.8 | 100 | 0 | 89 | 0 | US (67%)[⊥] | |
| 5 | B | 10 | 100 | 1 | 10 | 0 | Türkiye (99%) | No known deposits |
| 6 | $C_G$ | 200 | 99 | 3 | – | 2 | China (44%) | NO |
| 6 | C | 200 | 0 | – | – | 100 | Widespread | |
| 7 | N | 19 | 0 | – | – | 100 | Widespread | |
| 8 | O | 461,000 | 0 | – | – | 100 | Widespread | |
| 9 | F | 585 | 60 | 1 | 28 | 29 | Mexico (33%) | |
| 10 | Ne | 0.005 | 0 | – | – | 100 | Widely produced | |
| 11 | Na | 23,600 | 0 | – | – | 100 | Widely produced | |
| 12 | Mg | 23,300 | 100 | 13 | 83 | 0 | China (97%) | IT |
| 13 | Al | 82,300 | 58 | 32 | – | 17 | Guinea (62%) | |
| 14 | Si | 282,000 | 60 | 0 | 55 | 29 | Norway (33%) | |
| 15 | P | 1,050 | 82 | 0 | – | 13 | Morocco (27%) | |
| 16 | S | 350 | 0 | – | – | 100 | Poland (19%) | |
| 17 | Cl | 145 | 0 | – | – | 100 | Widely produced | |
| 18 | Ar | 3.5 | 0 | – | – | 100 | Widely produced | |
| 19 | K | 20,900 | 33 | – | – | 39 | Germany (56%) | |
| 20 | Ca | 41,500 | 0 | – | – | 100 | Widely produced | |
| 21 | Sc | 22 | 100 | 0 | 63 | 0 | China (67%)[⊥] | EL, FI |
| 22 | Ti | 2,650 | 100 | 1 | 83 | 1 | Kazakhstan (36%) | ES, SE, PL, RO |
| 23 | V | 120 | 100 | 6 | – | 4 | Russia (44%) | EE, FI, PL, SE |
| 24 | Cr | 102 | 7 | 21 | 83 | 85 | Finland (34%) | |
| 25 | Mn | 950 | 96 | 9 | – | 8 | South Africa (41%) | |
| 26 | Fe | 56,300 | 77 | 31 | – | 35 | Brazil (33%) | |
| 27 | Co | 25 | 81 | 22 | – | 16 | DRC (63%)[∀] | |
| 28 | Ni | 84 | 31 | 16 | – | 57 | Finland (38%) | |
| 29 | Cu | 60 | 48 | 55 | – | 71 | Poland (19%) | |
| 30 | Zn | 70 | 56 | 34 | – | 58 | Peru (13%) | |
| 31 | Ga | 19 | 98 | 0 | 69 | 1 | China (69%) | |
| 32 | Ge | 1.5 | 42 | 2 | 5 | 14 | China (83%) | |
| 33 | As | 1.8 | 39 | 0 | – | 41 | UK (44%) | |
| 34 | Se | 0.05 | 2 | 1 | – | 92 | Germany (34%) | |
| 35 | Br | 2.4 | 100 | – | – | 0 | Israel (44%)[⊥] | No known deposits |
| 36 | Kr | 0.0002 | 0 | – | – | 100 | Widely produced | |
| 37 | Rb | 90 | 100 | – | – | 10 | China (100%)[#] | |
| 38 | Sr | 370 | 0 | 0 | 58 | 100 | Spain (99%) | |
| 39 | Y | 33 | 100 | 31[∀] | 63 | 13 | China (64%) | |
| 40 | Zr | 165 | 100 | 12 | – | 7 | South Africa (40%) | FR, SE, EL |
| 41 | Nb | 20 | 100 | 0 | 87 | 0 | Brazil (82%) | AT, CZ, FI, FR, PT, Greenland |
| 42 | Mo | 1.2 | 100 | 30 | – | 14 | US (59%) | Greenland, EL, SE, PL |
| 43 | Tc | – | – | – | – | – | – | – |
| 44 | Ru | 0.001 | 100 | 12 | – | 1 | South Africa (94%) | FI, SE, PL, ES |
| 45 | Rh | 0.001 | 100 | 12 | 66 | 2 | South Africa (81%) | FI, SE, PL, ES |
| 46 | Pd | 0.015 | 100 | 12 | 51 | 7 | Russia (40%) | FI, SE, PL, ES |
| 47 | Ag | 0.075 | 5 | 4 | – | 72 | Poland (58%) | |

| Element | | Abundance (ppm)[ϒ] | *IR* (%) | *R* (%) | *C* (%)[ς] | $S_E$ | Largest EU Supplier | EU deposits[∀] |
|---|---|---|---|---|---|---|---|---|
| 48 | Cd | 0.15 | 8 | 30 | – | 89 | Netherlands (24%) | |
| 49 | In | 0.25 | 11 | 1 | 50 | 75 | France (38%) | |
| 50 | Sn | 2.3 | 0 | 31 | – | 100 | Portugal (40%) | |
| 51 | Sb | 0.2 | 100 | 28 | 71 | 11 | Türkiye (63%) | SE, DE, AT, FR, CZ, PT |
| 52 | Te | 0.001 | 0 | 1 | – | 100 | Canada (27%) | |
| 53 | I | 0.45 | 100 | – | – | 0 | Chile (66%)[⊥] | No known deposits |
| 54 | Xe | 0.00003 | 0 | – | – | 100 | Widely produced | |
| 55 | Cs | 3 | 100 | 0 | – | 0 | Canada (100%)[#] | DE |
| 56 | Ba | 425 | 74 | 0 | – | 16 | China (44%) | |
| 57 | La | 39 | 100 | 1[∀] | 67 | 0 | China (80%)[∀] | Greenland |
| 58 | Ce | 66.5 | 100 | 1[∀] | 67 | 0 | Russia (64%)[∀] | Greenland |
| 59 | Pr | 9.2 | 100 | 10[∀] | 63 | 2 | China (80%)[∀] | Greenland |
| 60 | Nd | 41.5 | 100 | 1[∀] | 95 | 0 | China (80%)[∀] | Greenland |
| 61 | Pm | – | – | – | – | – | – | – |
| 62 | Sm | 7 | 100 | 1[∀] | 95 | 0 | China (80%)[∀] | Greenland |
| 63 | Eu | 2 | 100 | 38[∀] | 63 | 16 | China (64%)[∀] | Greenland |
| 64 | Gd | 6.2 | 100 | 1[∀] | 63 | 0 | China (64%)[∀] | Greenland |
| 65 | Tb | 1.2 | 100 | 6[∀] | 63 | 2 | China (64%)[∀] | Greenland |
| 66 | Dy | 5.2 | 100 | 0[∀] | 63 | 0 | China (65%)[∀] | Greenland |
| 67 | Ho | 1.3 | 100 | 1[∀] | 63 | 0 | China (64%)[∀] | Greenland |
| 68 | Er | 3.5 | 100 | 1[∀] | 63 | 0 | China (64%)[∀] | Greenland |
| 69 | Tm | 0.5 | 100 | 1[∀] | 63 | 0 | China (64%)[∀] | Greenland |
| 70 | Yb | 3.2 | 100 | 1[∀] | 63 | 0 | China (64%)[∀] | Greenland |
| 71 | Lu | 0.8 | 100 | 1[∀] | 63 | 0 | China (64%)[∀] | Greenland |
| 72 | Hf | 3 | 0 | 0 | – | 100 | France (76%) | |
| 73 | Ta | 2 | 99 | 1 | – | 2 | DRC (35%) | ES |
| 74 | W | 1.25 | 21 | 42 | 58 | 88 | Austria (34%) | |
| 75 | Re | 0.0007 | 92 | 50 | – | 33 | Chile (49%)[⊥] | |
| 76 | Os | 0.0015 | 100 | 12 | – | 1 | South Africa (94%)[∀] | FI, SE, PL, ES |
| 77 | Ir | 0.001 | 100 | 12 | – | 1 | South Africa (94%) | FI, SE, PL, ES |
| 78 | Pt | 0.005 | 100 | 12 | 50 | 4 | South Africa (71%) | FI, SE, PL, ES |
| 79 | Au | 0.004 | 0 | 5 | – | 100 | Finland (28%) | |
| 80 | Hg | 0.085 | – | – | – | – | – | |
| 81 | Tl | 0.85 | – | – | – | – | Unknown | FR[#] |
| 82 | Pb | 14 | 21 | 83 | – | 94 | Poland (17%) | |
| 83 | Bi | 0.0085 | 71 | 0 | – | 16 | China (50%) | |

**Supplementary table 2 | Elemental abundance, suppliers and self-reliance.** List of elements from hydrogen to bismuth showing their abundance, import reliance (*IR*), EU end-of-life recycling input rate (R), average collection rate (*C*), self-reliance index ($S_E$) , largest EU supplier and the location of EU deposits. The elemental supply resilience is calculated according to equation (1) in the main text. Element 6, carbon, has two entries: one for naturally occurring graphite, $C_G$, and one for elemental carbon, C. All data in this table are taken from the annexes in the *Study on the Critical Raw Materials for the EU (2023)*, except where indicated by the following superscripts: [ϒ]Abundance data taken from Ref. [9]; [ς]Collection rates are taken from the "Update of the 2015 Material System Analyses– Executive summary."; [∀]SCRREEN factsheets: https://scrreen.eu/crms-2023/; [⊥]Global supply data; [#]USGS Mineral Commodity Summaries (2025).

**Supplementary Figure 1** | Periodic table (Crustal abundance redux)

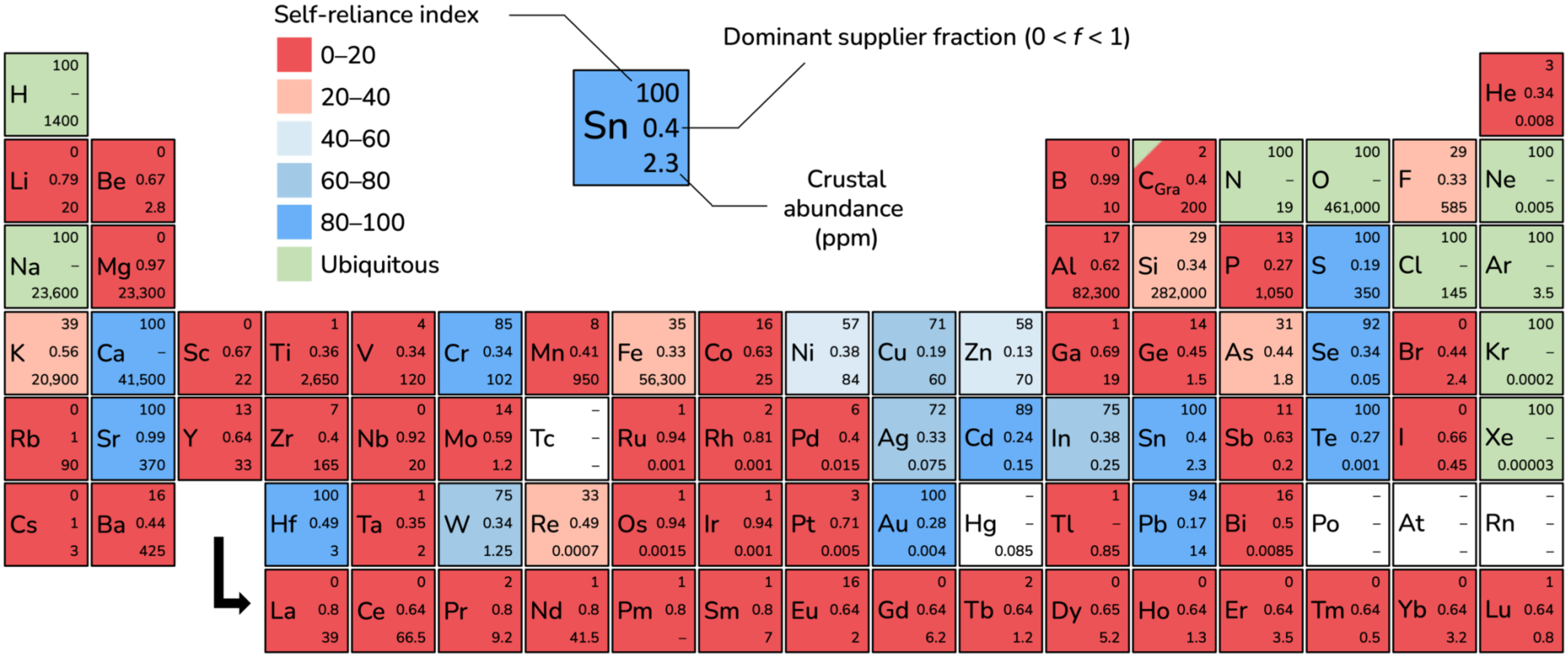


**Supplementary figure 1** | **Periodic table with crustal abundance redux.** Table of elements colour-coded by their self-reliance index. The dominant supplier fraction, self-reliance index, and crustal abundance are given for each element.

**Supplementary Figure 2** | Environmental performance index

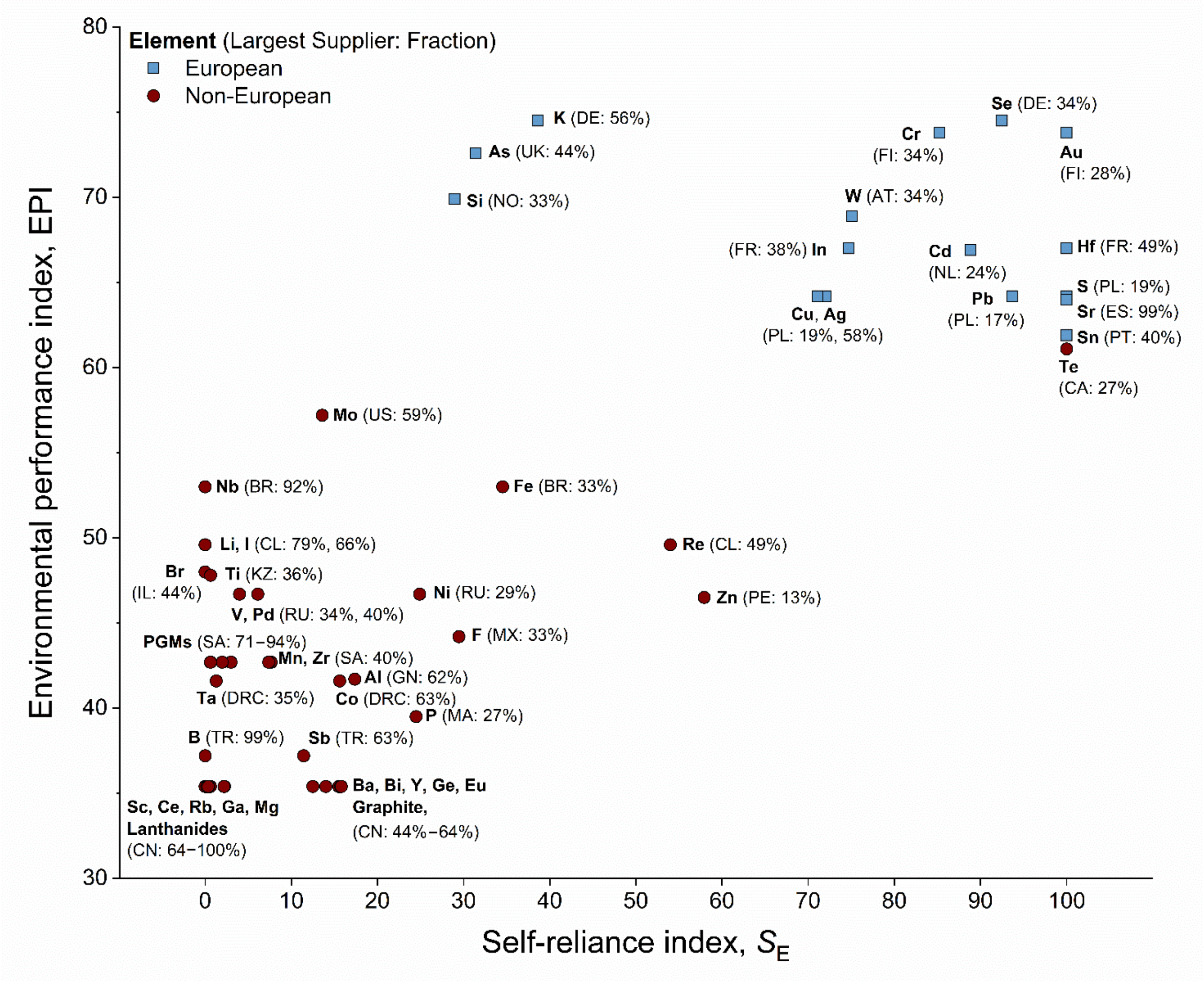


**Supplementary figure 2** | **Environmental sustainability.** Plot of the environmental performance index (*EPI*) as a function of the elemental self-reliance index ($S_E$). The dominant EU supplier is indicated in brackets and identified using the top-level domain short-code used for internet addresses. As discussed in the main text, this graph plots the *EPI* against the self-reliance index for each element where data is available. Many imported elements tend to come from a small number of suppliers and from countries with low *EPI* scores, while those elements that are sourced domestically tend to come from countries with higher *EPI* scores. This emphasises the need to shift extraction and processing to European countries wherever possible.

**Supplementary Note 1** | Aggregating self-reliance index scores

Consider a compound with the stoichiometric formula $A_aB_bC_c$ composed of elements with self-reliance indices, $S_A$, $S_B$, $S_C \in [0, 100]$. To compute the geometric mean over these indices, each element must be represented according to its stoichiometric coefficient. For example, the formula $A_2B$ corresponds to the multiset (A, A, B). The geometric mean over this multiset is mathematically equivalent to a weighted geometric mean over the unique set (A,B), where the weights are the stoichiometric coefficients (2, 1). Thus, by applying these stoichiometric weights within the geometric mean, we establish a direct, formula-derived weighting scheme. This chemistry-driven approach is more fundamental than allocations based on economic or mass measures and directly links the nanomaterial's composition to its calculated self-reliance profile. To justify the use of the geometric mean in aggregating the self-reliance scores of individual elements into a compound, we compare its behaviour with that of the arithmetic mean. For our generic compound $A_aB_bC_c$, the arithmetic mean, *AM*, is given by supplementary equation (1).

$$AM = \frac{aS_A + bS_B + cS_B}{a + b + c} \qquad (1)$$

with the geometric mean, *GM*, given by supplementary equation (2).

$$GM = \left(S_A^a \times S_B^b \times S_C^c\right)^{1/(a+b+c)} \qquad (2)$$

We consider four illustrative scenarios of elemental self-reliance: one where all elements have similar indices; a second where a single element dominates with a high index; a third where one element is substantially weaker than the others; and a fourth where one element has a self-reliance index of zero, as summarised in the table below.

| Case | $S_A$ | $S_B$ | $S_C$ | AM | GM |
|---|---|---|---|---|---|
| 1. Balanced | 60 | 65 | 70 | 65 | 64.8 |
| 2. One strong | 100 | 30 | 30 | 53.3 | 44.8 |
| 3. One weak | 90 | 90 | 10 | 63.3 | 43.3 |
| 4. One zero | 90 | 90 | 0 | 60 | 0 |

When the elemental self-reliance indices are similar, both aggregation methods produce nearly identical results. In the case of a single strong element, the arithmetic mean is more susceptible to the influence of this high outlier. Conversely, the presence of a single weak element substantially reduces the geometric mean relative to the arithmetic mean. Most critically, the inclusion of any element with a self-reliance index of zero sends the geometric mean for the compound to zero, correctly indicating that synthesis is completely dependent on a non-European source. The arithmetic mean, in contrast, remains non-zero and would thus overestimate the resilience of such a compound. The geometric mean therefore exhibits the two properties we desire for this metric: it is not unduly inflated by a single high-score component, and it falls to zero when any component is fully imported, providing a clear signal of supply-chain vulnerability.

*The case of compounds containing ubiquitous elements*

A drawback of using the geometric mean to compute compound self-reliance arises in context of ubiquitous elements such as oxygen. As such an element is assigned an elemental self-reliance index of $S_E = 100$, and owing to many advanced materials containing oxygen (in particular the metal oxide family containing oxide stoichiometries of 4 or 5), the compound stoichiometry can be dominated by the ubiquitous element meaning the geometric mean will inflate a compound's self-reliance index

disproportionately and may mask the deleterious contribution of a low $S_E$ element. Taking the example of $V_2O_5$, vanadium is heavily imported from Russia and China and has a self-reliance index of 4, putting it among the lowest scoring elements. However, including oxygen in the calculation of the compound self-reliance for $V_2O_5$ gives $S_C$ = $(4x4x100x100x100x100x100)^{1/7}$ = 40. As this compound is clearly dependent on the supply of vanadium, the inclusion of oxygen obscures its low self-reliance index.

We demonstrate this in Supplementary Figure 3 where we plot the compound self-reliance scores for a range of oxygen-containing nanomaterials, where the x-axis is the $S_C$ computed including an oxygen self-reliance index of 100 and the y-axis is the $S_C$ computed treating oxygen neutrally (i.e., ignoring its contribution rather than setting it to zero). We see that the values roughly follow a power law as a result of using the geometric mean, which leads to large deviations from the line of y = x for values in the range of 10 > $S_C$ > 90. This means the self-reliance scores for compounds such as $MoO_3$, $V_2O_5$, and $InGaZnO_4$ are significantly inflated as a result of including oxygen in the calculations.

Regarding the designation "strategic" for materials with an $S_C$ > 60, the inclusion of oxygen in the computation of $S_C$ in Supplementary Figure 3 would mean four materials ($MoO_3$, $BaSnO_3$, $Na_2FeP_2O_7$, and ZnO) would be considered strategic owing to the inflationary effect of the high oxygen index, while excluding it means their scores fall below the strategic threshold.

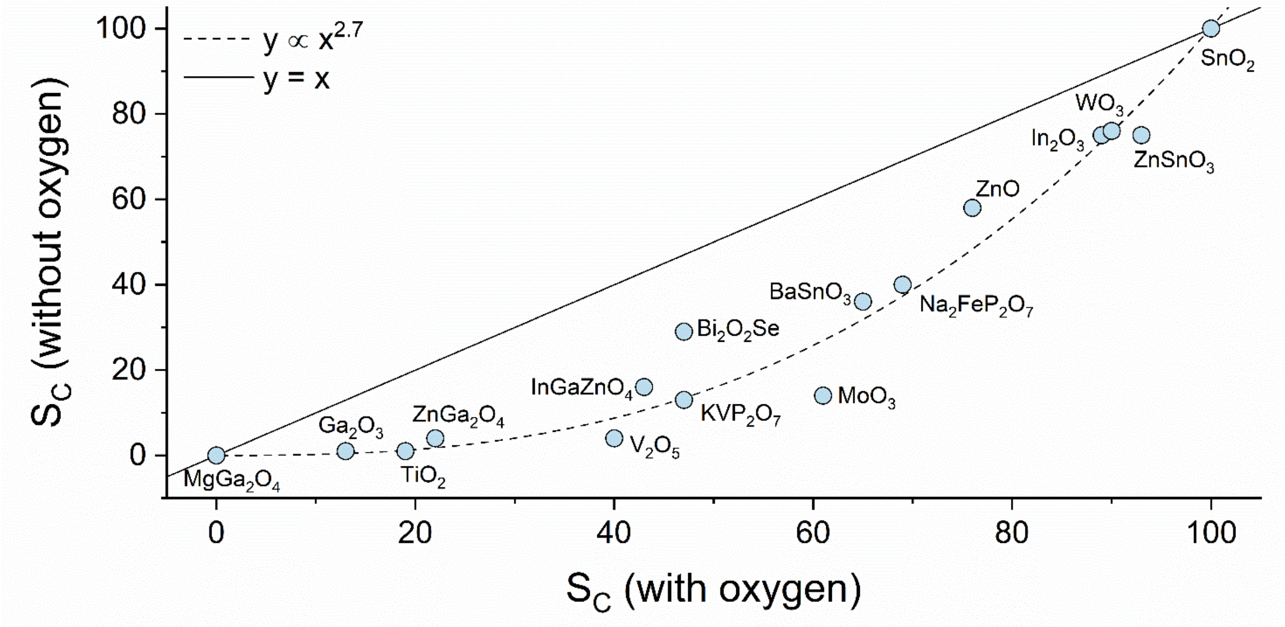


**Supplementary figure 3 | The effect of oxygen on compound calculations.** Compound self-reliance indices ($S_C$) are computed for the materials shown using equation (3) in the main text, with the x-axis including oxygen and the y axis treating it neutrally.

To eliminate the inflationary effects of elements that are common in advanced materials and are available planet-wide, we require a specific definition for "ubiquitous" that captures this. To the best of the authors' knowledge, a definition for ubiquitous in the context of criticality assessments has not been needed as the materials of interest are defined by their scarcity. However, advanced materials often have elements such as oxygen as core components in their structure, meaning if we are to treat such elements neutrally, we need a definition to identify them.

In the context of the composition of advanced materials, we define a "ubiquitous" element as one that:

1. is derived from planetary-scale reservoirs such as the atmosphere or oceans
2. is geographically and politically dispersed
3. is extractable using simple and widely implemented physical or chemical processes (e.g., those that can be taught at an undergraduate level)
4. is routinely purifiable to high grade (≥ 4N) using non-proprietary technologies and without requiring significant energy inputs
5. is not susceptible to long-term monopolisation through geological, chemical, or technological advantages

Via this definition, we identify H, C, N, O, Na, Cl and the noble gases Ne, Ar, K, and Xe (He is excluded as its extraction is tied to specific gas fields) as ubiquitous and are therefore treated neutrally in the compound calculations.

**Supplementary Table 3** | Conductive materials

| | Compound | Self-reliance, $S_C$ | Electrical Conductivity (S $m^{-1}$) | Reference |
|---|---|---|---|---|
| MXenes | $Mo_2AuC$ | 26 | 435 | Ref.[10] |
| | $Mo_2GaC$ | 5 | 80 | Ref.[10] |
| | $Mo_2GaN$ | 5 | 208300 | Ref.[10] |
| | $Nb_2AlC$ | 0 | 0.164 | Ref.[10] |
| | $Nb_4AlC_3$ | 0 | 217 | Ref.[10] |
| | $Ta_4AlC_3$ | 2 | 47.6 | Ref.[10] |
| | $Ti_2AlC$ | 2 | 14.7 | Ref.[10] |
| | $Ti_2AlN$ | 2 | 525000 | Ref.[10] |
| | $Ti_3AlC_2$ | 1 | 277000 | Ref.[11] |
| | $Ti_3GaC_2$ | 1 | 104000 | Ref.[12] |
| | $Ti_3GeC_2$ | 1 | $1.51 \times 10^6$ | Ref.[13] |
| | $V_2AlC$ | 7 | 38460 | Ref.[10] |
| | $V_2GaN$ | 2 | 416600 | Ref.[10] |
| Conductors | Graphene | 100 | $1 \times 10^2 – 1.3 \times 10^5$ | Ref.[14, 15] |
| | AgNPs | 72 | $1.8 \times 10^6 – 2.5 \times 10^7$ | Refs[16, 17]. |
| | AgNWs | 72 | $1.2 \times 10^5 – 5 \times 10^6$ | Refs.[18, 19] |
| | AuNPs | 100 | $4 \times 10^4 – 8 \times 10^6$ | Refs.[20, 21] |
| | NiNWs | 57 | 74626 | Ref.[22] |
| | CNTs | 100 | $2 \times 10^2 – 6.7 \times 10^5$ | Refs.[23, 24] |
| | 1T-$MoS_2$ | 51 | 1100 | Ref.[25] |
| | $MgB_2$ | 0 | 95000 | Ref.[26] |
| | Al | 17 | $4.13 \times 10^7$ | Ref.[27] |
| | Ti | 1 | $2.56 \times 10^6$ | Ref.[27] |
| | Pt | 3 | $1.02 \times 10^7$ | Ref.[27] |
| | Cu | 71 | $6.49 \times 10^7$ | Ref.[27] |
| | Ni | 57 | $1.62 \times 10^7$ | Ref.[27] |
| | W | 75 | $2.07 \times 10^7$ | Ref.[27] |
| | ITO | 83 | 330000 | Ref.[28] |
| | Ag | 72 | $6.8 \times 10^7$ | Ref.[27] |
| | Au | 100 | $4.88 \times 10^7$ | Ref.[27] |
| | FTO | 54 | 130000 | Ref.[29] |
| | AgNS | 72 | $6 \times 10^6 – 1.5 \times 10^7$ | Ref.[30] |
| | CuNPs | 71 | $7.1 \times 10^6$ | Ref.[31] |

**Supplementary table 3** | **Conductive materials.** Thin-film conductivity and self-reliance index ($S_C$) of conductive nanomaterials.

**Supplementary Table 4** | Dielectric materials

| | Compound | Self-reliance, $S_C$ | Dielectric Constant | Reference |
|---|---|---|---|---|
| Dielectric Materials | $Al_2O_3$ | 17 | 9 | Ref.[32] |
| | BaO | 16 | 32 | Ref.[32] |
| | $BaTiO_3$ | 4 | 500 | Ref.[33] |
| | $BaZrO_3$ | 11 | 4 | Ref.[34] |
| | BiOCl | 16 | 70 | Ref.[35] |
| | h-BN | 0 | 4.2 | Ref.[36] |
| | Cellulose | 100 | 7.5 | Ref.[37] |
| | Chitosan | 100 | 8 | Ref.[38] |
| | Alginate | 100 | 35 | Ref.[39] |
| | $HfO_2$ | 100 | 25 | Ref.[32] |
| | $HfSiO_4$ | 54 | 11 | Ref.[32] |
| | Kaolin | 72 | 5.12 | Ref.[40] |
| | $La_2O_3$ | 1 | 30 | Ref.[32] |
| | $LaAlO_3$ | 4 | 30 | Ref.[32] |
| | MMT | 100 | 5.7 | Ref.[41] |
| | Muscovite | 100 | 9.3 | Ref.[42] |
| | $PbTiO_3$ | 10 | 6.25 | Ref.[34] |
| | $Si_3N_4$ | 29 | 7 | Ref.[32] |
| | $SiO_2$ | 29 | 3.9 | Ref.[32] |
| | $SrTiO_3$ | 1 | 2000 | Ref.[32] |
| | $Ta_2O_5$ | 93 | 22 | Ref.[32] |
| | Talc | 1 | 4.7 | Ref.[43] |
| | $Ti_2O_3$ | 1 | 125 | Ref.[44] |
| | $TiO_2$ | 0 | 80 | Ref.[32] |
| | $Y_2O_3$ | 7 | 15 | Ref.[32] |
| | $ZrO_2$ | 44 | 25 | Ref.[32] |
| | $CaF_2$ | 17 | 8.43 | Ref.[45] |

**Supplementary table 4 | Dielectric materials.** Dielectric constant and self-reliance index ($S_C$) of insulating nanomaterials.

**Supplementary Table 5** | Semiconducting materials: Single crystals

| | Compound | $S_C$ | Carrier Mobility ($cm^2 V^{-1} s^{-1}$) | Synthesis Technique | Layers (L) or thickness (nm) | Reference |
|---|---|---|---|---|---|---|
| **2D Materials** | As | 31 | 51 | Mechanical Exfoliation | 1 L | Ref.[46] |
| | Bi | 16 | 220 | Pulsed Laser Deposition | ~4 L | Ref.[47] |
| | $Bi_2Se_2$ | 38 | 400 | Vapor Phase Epitaxy | 40 nm | Ref.[48] |
| | $Bi_2Te_3$ | 48 | 800 | Metal Organic CVD | 32 nm | Ref.[49] |
| | $Bi_4Te_6$:Cs | 0 | 1000 | - | Bulk | Ref.[50] |
| | $CrSiTe_3$ | 76 | 27 | - | Bulk | Ref.[51] |
| | GaS | 8 | 0.1 | Mechanical Exfoliation | Few Layer | Ref.[52] |
| | GaSe | 8 | 0.6 | Mechanical Exfoliation | Few Layer | Ref.[52] |
| | GeSe | 36 | 85 | Vapor Liquid–Solid Growth | <40 nm | Ref.[53] |
| | GeAs | 21 | 0.6 | Mechanical Exfoliation | 6 nm | Ref.[54] |
| | GeP | 2 | 0.03 | LPE | 5.7 nm | Ref.[55] |
| | $HfS_2$ | 100 | 45 | Mechanical Exfoliation | 4-5 L | Ref.[56] |
| | $HfSe_2$ | 95 | 3 | Mechanical Exfoliation | 6 L | Ref.[57] |
| | $HfS_3$ | 94 | 1.20E-05 | Mechanical Exfoliation | ~11 L | Ref.[58] |
| | $In_2Se_3$ | 85 | 30 | Mechanical Exfoliation | ~15 L | Ref.[59] |
| | InSe | 83 | 1000 | Mechanical Exfoliation | 6 L | Ref.[60] |
| | $MoS_2$ | 51 | 321 | Mechanical Exfoliation | 4–5 L | Ref.[61] |
| | $MoSe_2$ | 49 | 86 | LPE | 13-17 L | Ref.[62] |
| | $MoTe_2$ | 51 | 45 | CVD | ~7 L | Ref.[63] |
| | $MoO_3$ | 14 | 1100 | Mechanical Exfoliation | 8 L | Ref.[64] |
| | $NiI_2$ | 0 | 0.16 | Physical Vapor Deposition | 8 L | Ref.[65] |
| | $NiPS_3$ | 55 | 10 | LPE | 10-12 L | Ref.[66] |
| | BP | 24 | 984 | Pulsed Laser Deposition | 10 nm | Ref.[67] |
| | $PdS_2$ | 39 | 2.85 | Sulfurisation of Pd thin films | 25 L | Ref.[68] |
| | PdPS | 25 | 14.4 | Mechanical Exfoliation | 10 L | Ref.[69] |
| | PdPSe | 24 | 21.37 | Mechanical Exfoliation | 10 L | Ref.[70] |
| | $PtS_2$ | 31 | 62.5 | Mechanical Exfoliation | 8 L | Ref.[71] |
| | $PtSe_2$ | 30 | 210 | Mechanical Exfoliation | 12 L | Ref.[72] |
| | $ReS_2$ | 81 | 12 | Mechanical Exfoliation | 2 L | Ref.[73] |
| | $ReSe_2$ | 77 | 9.78 | Mechanical Exfoliation | 1 L | Ref.[74] |
| | $Sb_2Se_3$ | 40 | 1.25 | Thermal Evaporation | 3100 nm | Ref.[75] |
| | $Sb_2Te_2Se$ | 41 | 55.5 | Mechanical Exfoliation | 181 nm | Ref.[76] |
| | $Sb_2Te_3$ | 42 | 27 | Mechanical Exfoliation | 20 nm | Ref.[77] |
| | SiAs | 30 | 0.039 | Mechanical Exfoliation | 20 nm | Ref.[78] |
| | SnO | 100 | 3 | Liquid-metal Exfoliation | 1 L | Ref.[79] |
| | SnS | 100 | 35 | Liquid-metal Exfoliation | 1 L | Ref.[80] |
| | $SnS_2$ | 100 | 50 | CVD | 1 L | Ref.[81] |
| | SnSe | 96 | 254 | Mechanical Exfoliation | 10 nm | Ref.[82] |
| | $SnSe_2$ | 95 | 85 | Mechanical Exfoliation | 8.6 nm | Ref.[83] |
| | $SnBi_2Se_4$ | 56 | 35 | Melt Growth | 1 mm | Ref.[84] |
| | $SnSb_2Te_4$ | 54 | 317 | CVD | 1 L | Ref.[85] |
| | $TiS_3$ | 28 | 25 | Mechanical Exfoliation | 6.4 nm | Ref.[86] |

| | | | | | | |
|---|---|---|---|---|---|---|
| | $V_2O_5$ | 4 | 7 | Mechanical Exfoliation | 40 nm | Ref.[87] |
| | $WS_2$ | 91 | 83 | Mechanical Exfoliation | 1 L | Ref.[88] |
| | $WSe_2$ | 86 | 202 | Mechanical Exfoliation | 1 L | Ref.[89] |
| | $WTe_2$ | 91 | 492 | Mechanical Exfoliation | 8.25 nm | Ref.[90] |
| | ZnCl | 58 | 12 | | | Ref.[91] |
| | | | | | | |
| **Organics** | Rubrene | 100 | 45 | - | - | Ref.[92] |
| | Pentacene | 100 | 5.2 | - | - | Ref.[92] |
| | TIPS-PEN | 100 | 11 | - | - | Ref.[92] |
| | C8-BTBT | 100 | 30 | - | - | Ref.[92] |
| | | | | | | |
| **Metal Oxides** | $Ga_2O_3$ | 1 | 160 | - | - | Ref.[93] |
| | $In_2O_3$ | 75 | 213 | - | - | Ref.[93] |
| | ZnO | 58 | 187 | - | - | Ref.[93] |
| | $SnO_2$ | 100 | 240 | - | - | Ref.[93] |
| | $ZnSnO_3$ | 76 | 126 | - | - | Ref.[93] |
| | $BaSnO_3$ | 40 | 225 | - | - | Ref.[93] |
| | $MgGa_2O_4$ | 0 | 7 | - | - | Ref.[93] |
| | $ZnGa_2O_4$ | 4 | 107 | - | - | Ref.[93] |
| | $InGaZnO_4$ | 16 | 127 | - | - | Ref.[93] |
| | | | | | | |
| | Silicon | 29 | 2000 | - | - | - |
| | | | | | | |
| **1D Nanowires** | GaAs | 4 | 5000 | - | - | Ref.[94] |
| | Si | 29 | 1350 | - | - | Ref.[94] |
| | InGaSb | 8 | 463 | - | - | Ref.[95] |
| | GaSb | 3 | 400 | - | - | Ref.[96] |
| | $InGaO_3$ | 9 | 750 | - | - | Ref.[97] |
| | InAs | 48 | 6000 | - | - | Ref.[98] |
| | Ge | 14 | 600 | - | - | Ref.[99] |
| | GaN | 1 | 3100 | - | - | Ref.[100] |

**Supplementary table 5 | Semiconducting materials: Single crystals.** Self-reliance index ($S_C$) and carrier mobility for semiconducting nanosheets, organics, metal oxides and 1D nanowires. The exfoliation method and nanosheet thickness/layer number are indicated for the 2D materials.

**Supplementary Table 6** | Semiconducting materials: Thin films

| | Compound | Self-reliance, $S_C$ | Carrier Mobility ($cm^2 V^{-1} s^{-1}$) | Reference |
|---|---|---|---|---|
| 2D: Solution processed | InSe | 83 | 125 | Ref.[101] |
| | $MoS_2$ | 51 | 30 | Ref.[102] |
| | $WS_2$ | 91 | 8.48 | Ref.[103] |
| | $MoWSe_2$ | 54 | 5.6 | Ref.[103] |
| | $MoSe_2$ | 49 | 2.8 | Ref.[103] |
| | $MoTe_2$ | 51 | 2 | Ref.[103] |
| | $PtSe_2$ | 30 | 1.11 | Ref.[103] |
| | $MoSe_2$:Nb | 0 | 0.79 | Ref.[103] |
| | $WSe_2$:Nb | 0 | 2.3 | Ref.[103] |
| | $WSe_2$ | 86 | 1.73 | Ref.[103] |
| | BP | 13 | 0.0013 | Ref.[103] |
| | Te | 100 | 0.001 | Ref.[103] |
| | $MoO_3$ | 14 | 600 | Ref.[104] |
| | $SnSe_2$ | 95 | 20 | Ref.[105] |
| | | | | |
| Metal Oxides | ZnO | 58 | 70 | Ref.[106] |
| | InO | 75 | 41.8 | Ref.[107] |
| | IGZO | 16 | 14 | Ref.[108] |
| | ISnZO | 76 | 70 | Ref.[109] |
| | $TiO_2$ | 1 | 18.6 | Ref.[110] |
| | $WO_3$ | 75 | 6 | Ref.[111] |
| | $BaSnO_3$:La | 0 | 128 | Ref.[112] |
| | | | | |
| 2D: Grown | BP | 13 | 160 | Ref.[113] |
| | $PtSe_2$ | 30 | 625 | Ref.[114] |
| | $MoSe_2$ | 49 | 121 | Ref.[115] |
| | $SnS_2$ | 100 | 50 | Ref.[81] |
| | $WSe_2$ | 86 | 82 | Ref.[116] |
| | $Bi_2O_2Se$ | 29 | 1400 | Ref.[117] |
| | Bi | 16 | 220 | Ref.[47] |
| | | | | |
| Organics | Pentacene | 100 | 10.4 | Ref.[118] |
| | PEDOT:PSS | 100 | 8.12 | Ref.[119] |
| | Rubrene | 100 | 5 | Ref.[120] |
| | | | | |
| 1D: Networks | CNTs | 100 | 20.2 | Ref.[121] |
| | ZnO | 58 | 25 | Ref.[122] |
| | Si | 29 | 119 | Ref.[123] |
| | InAs | 48 | 93.6 | Ref.[124] |
| | $WS_2$ | 91 | 50 | Ref.[125] |
| | | | | |
| Quantum Dots | PbSe | 93 | 24 | Ref.[126] |
| | CdSe | 91 | 0.1 | Ref.[127] |
| | PbS | 97 | 3.7 | Ref.[128] |
| | SiGe | 20 | 140 | Ref.[129] |

**Supplementary table 6 | Semiconducting materials: Thin films.** Self-reliance index ($S_C$) and carrier mobility for semiconducting films comprising solution deposited and grown nanosheets, metal oxides, organics, 1D nanowires and quantum dots.

**Supplementary Note 2** | Semiconducting materials: Theoretical mobilities

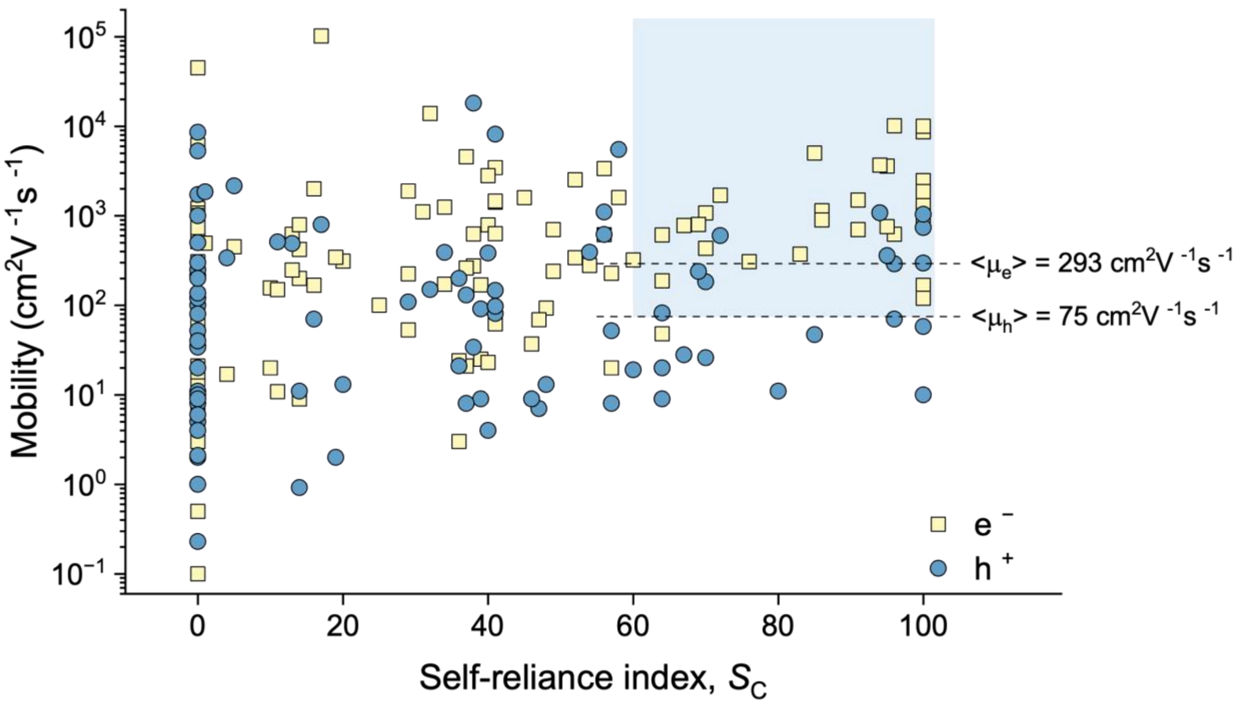


**Supplementary figure 4 | Theoretical mobilities for single crystal 2D materials.** The critical mobilities indicated are calculated using the geometric mean of all electron, $\mu_e$, and all hole, $\mu_h$, values, with the blue region indicating critical nanomaterials.

In addition to the experimental mobility values for the semiconductors in Figure 3c and 3d in the main text, we also sought theoretically predicted mobilities for the 336 semiconducting materials ($E_G$ < 2.5 eV) in the Materials Cloud 2D Database.[130] Here, we found values for 132 materials, with the self-reliance index, mobilities, and references shown in Supplementary Table 7. Almost a third of these materials (39) are composed of fully imported elements that are not recycled ($S_C$ = 0), as shown in Supplementary Figure 4.

To apply our criteria for identifying a "strategic" nanomaterial (see Methods), we take the geometric mean of the electron and hole mobilities, finding critical performance thresholds of $\mu_{e,C}$ = 293 cm$^2$ V$^{-1}$ s$^{-1}$ and $\mu_{h,C}$ = 75 cm$^2$ V$^{-1}$ s$^{-1}$. Taking Europe to be self-reliant for materials with $S_E$ values > 60, this leaves 28 strategic 2D materials, of which 18 are p-type, 2 are n-type, and 8 are ambipolar. We have highlighted these in blue in Supplementary Table 7. Of the >3000 materials in the Materials Cloud 2D Database, this provides a significant down-selection, allowing a small number of promising materials to be targeted for development.

| Compound | Self-reliance, $S_C$ | Electron Mobility ($cm^2 V^{-1} s^{-1}$) | Hole Mobility ($cm^2 V^{-1} s^{-1}$) | Reference |
|---|---|---|---|---|
| AgBr | 0 | 15 | 6 | Ref.[131] |
| $AgF_2$ | 39 | 25 | 9 | Ref.[131] |
| As | 41 | 5290–12320 | 330–350 | Ref.[132] |
| $As_2S_3$ | 70 | 1075 | – | Ref.[133] |
| $As_2Se_3$ | 67 | 779 | – | Ref.[133] |
| $As_2Te_3$ | 70 | 433 | 183 | Ref.[133] |
| AuSe | 96 | 10110 | 70 | Ref.[134] |
| Bi | 16 | 168 | 70 | Ref.[131] |
| $Bi_2Te_2S$ | 48 | 93 | 13 | Ref.[131] |
| $Bi_2Te_2Se$ | 47 | 69 | 7 | Ref.[131] |
| $Bi_2TeSe_2$ | 46 | 37 | 9 | Ref.[131] |
| BiOI | 0 | 21 | 5 | Ref.[131] |
| BiTeCl | 40 | 23 | 4 | Ref.[131] |
| Graphane | 54 | 278 | 391 | Ref.[135] |
| $CdI_2$ | 0 | 5 | 4 | Ref.[131] |
| $CdInGaS_4$ | 49 | 700 | – | Ref.[136] |
| CrSBr | 0 | – | 5300 | Ref.[137] |
| $Cu_2Te$ | 80 | – | 11 | Ref.[138] |
| CuI | 0 | 18 | 8 | Ref.[131] |
| $Ga_2S_3$ | 16 | 657 | 694 | Ref.[139] |
| GaGeTe | 16 | 2000 | – | Ref.[140] |
| GaS | 11 | 10.85 | – | Ref.[141] |
| GaSe | 10 | 156 | – | Ref.[140] |
| GaTe | 10 | 20 | – | Ref.[142] |
| GaTeCl | 14 | 9 | 11 | Ref.[131] |
| $GeI_2$ | 0 | 245 | 123 | Ref.[143] |
| GeS | 37 | 21 | 8 | Ref.[131] |
| GeSe | 36 | 24 | 21 | Ref.[131] |
| $GeAs_2$ | 29 | 224 | – | Ref.[144] |
| GeAsSe | 38 | 275 | 18150 | Ref.[145] |
| $GeBi_2Te_4$ | 45 | 1594 | – | Ref.[146] |
| GeP | 13 | 620 | – | Ref.[147] |
| $GeSb_2Te_4$ | 40 | 792 | – | Ref.[146] |
| HfNBr | 0 | 128 | 100 | Ref.[131] |
| HfNCl | 100 | 167 | 739 | Ref.[148] |
| HfNI | 0 | 65 | 120 | Ref.[131] |
| $HfS_2$ | 100 | 1833 | – | Ref.[149] |
| $HfSe_2$ | 95 | 3579 | – | Ref.[149] |
| $HfGeTe_4$ | 72 | 1700 | 600 | Ref.[150] |
| $HfS_3$ | 100 | 960 | 10 | Ref.[151] |
| $HfSe_3$ | 94 | 3670 | 1080 | Ref.[152] |
| $In_2Se_3$ | 85 | 5022 | 47 | Ref.[153] |
| InSe | 83 | 373 | – | Ref.[154] |
| InTe | 86 | 1138 | 63 | Ref.[155] |
| KAgSe | 64 | 188 | 82 | Ref.[131] |
| $KPt_2Se_3$ | 25 | 100 | – | Ref.[156] |
| $LiAlTe_2$ | 0 | 237 | 80 | Ref.[131] |

| | | | | |
|---|---|---|---|---|
| $Mg_2B_2S_5$ | 0 | 6144 | 8 | Ref.[157] |
| $Mg_2Al_2Se_5$ | 0 | 630 | 2.5 | Ref.[157] |
| $Mg_2Ga_2S_5$ | 0 | 1208 | 12 | Ref.[157] |
| $MgAl_2S_4$ | 0 | 745 | 20 | Ref.[157] |
| $MgAl_2Se_4$ | 0 | 561 | 36 | Ref.[158] |
| $MgGa_2Se_4$ | 0 | 1070 | 215 | Ref.[159] |
| $MnPSe_3$ | 38 | 626 | 35 | Ref.[160] |
| $MoS_2$ | 52 | 340 | – | Ref.[149] |
| $MoSe_2$ | 49 | 240 | – | Ref.[149] |
| $MoTe_2$ | 52 | 2526 | – | Ref.[149] |
| $MoO_2$ | 14 | 200 | – | Ref.[140] |
| $MoO_3$ | 14 | 793 | 396 | Ref.[161] |
| $N_4$ | 100 | 8674 | 0.9 | Ref.[162] |
| $NbOBr_2$ | 0 | 515 | 10 | Ref.[163] |
| $NbOCl_2$ | 0 | 242 | 0.23 | Ref.[163] |
| NbOClBr | 0 | 100 | 1 | Ref.[164] |
| NbOClI | 0 | 99 | 9 | Ref.[164] |
| NbOBrI | 0 | 106 | 53 | Ref.[164] |
| $NbOI_2$ | 0 | 1005 | 137 | Ref.[163] |
| $NbS_2Cl_2$ | 0 | 308 | 6 | Ref.[165] |
| $NiO_2$ | 57 | 21 | 9 | Ref.[166] |
| $NiPSe_3$ | 57 | 228 | 52 | Ref.[167] |
| OsNCl | 1 | 67000 | 1200 | Ref.[168] |
| P | 13 | 248 | 490 | Ref.[140] |
| $PdS_2$ | 39 | 169 | 92 | Ref.[169] |
| $PdSe_2$ | 37 | 4559 | – | Ref.[170] |
| PdPS | 20 | 312 | 13 | Ref.[171] |
| PdPSe | 19 | 344 | 2 | Ref.[171] |
| $PtO_2$ | 4 | 17 | 339 | Ref.[171] |
| $PtS_2$ | 31 | 1107 | – | Ref.[149] |
| $PtSe_2$ | 29 | 1892 | – | Ref.[149] |
| $ReS_2$ | 69 | 799 | 239 | Ref.[172] |
| RhTeCl | 14 | 420 | 0.92 | Ref.[173] |
| Sb | 11 | 150 | 510 | Ref.[174] |
| $Sb_2S_3$ | 41 | 1420 | 97.5 | Ref.[175] |
| $Sb_2Te_2S$ | 41 | 1460 | – | Ref.[176] |
| $Sb_2Te_2Se$ | 41 | 3451 | 147 | Ref.[177] |
| $Sb_2Te_3$ | 41 | 630 | 8170 | Ref.[178] |
| $Sb_2TeSe_2$ | 40 | 2806 | 383 | Ref.[177] |
| SbTeI | 0 | 739 | 34 | Ref.[179] |
| $SbI_3$ | 0 | 90 | 2.1 | Ref.[180] |
| $Sc_2CCl_2$ | 0 | 45000 | – | Ref.[181] |
| $ScPS_4$ | 0 | – | 8580 | Ref.[182] |
| ScSeCl | 0 | 0.5 | 200 | Ref.[183] |
| ScSeBr | 0 | 6 | 250 | Ref.[183] |
| ScSeI | 0 | 4 | 300 | Ref.[183] |
| ScTeI | 0 | 10 | 500 | Ref.[183] |
| ScSI | 0 | 0.1 | 1000 | Ref.[183] |

| | | | | |
|---|---|---|---|---|
| SiH | 29 | 53 | 109 | Ref.[184] |
| SiAs | 34 | 1250 | 390 | Ref.[185] |
| $SiAs_2$ | 37 | 260 | 130 | Ref.[186] |
| $SiP_2$ | 17 | 102144 | 796 | Ref.[187] |
| SiPS | 34 | 172 | – | Ref.[188] |
| SnO | 100 | 2473 | 58 | Ref.[189] |
| SnS | 100 | 10000 | 58 | Ref.[190] |
| $SnS_2$ | 100 | 1398 | 296 | Ref.[131] |
| SnSe | 96 | 620 | 290 | Ref.[190] |
| $SnSe_2$ | 95 | 759 | 358 | Ref.[131] |
| SnTe | 100 | 1866 | 1037 | Ref.[191] |
| $Sn_2Te_2$ | 100 | 120 | – | Ref.[140] |
| $SnBi_2Se_4$ | 56 | 615 | 618 | Ref.[192] |
| $SnI_2$ | 0 | 42 | 40 | Ref.[193] |
| $SnP_2S_6$ | 64 | 48 | 20 | Ref.[194] |
| $SnP_2Se_6$ | 60 | 320 | 19 | Ref.[194] |
| $SnP_2Te_6$ | 64 | 610 | 9 | Ref.[194] |
| $SnSb_2Te_4$ | 56 | 3370 | 1100 | Ref.[195] |
| TiNBr | 0 | 739 | 1723 | Ref.[131] |
| TiNCl | 1 | 493 | 1852 | Ref.[131] |
| TiNF | 5 | 451 | 2156 | Ref.[196] |
| $TiS_3$ | 32 | 13870 | 150 | Ref.[197] |
| $WS_2$ | 91 | 700 | – | Ref.[198] |
| $WSe_2$ | 86 | 900 | – | Ref.[198] |
| $WTe_2$ | 91 | 1500 | – | Ref.[198] |
| $Y_2CBr_2$ | 0 | 19060 | 77 | Ref.[199] |
| YTeBr | 0 | 3 | 300 | Ref.[183] |
| YTeCl | 36 | 3 | 200 | Ref.[183] |
| YTeI | 0 | 10 | 250 | Ref.[183] |
| ZnCl | 58 | 1600 | 5500 | Ref.[168] |
| $ZnI_2$ | 0 | 106 | 78 | Ref.[131] |
| $Zn_2Te_2$ | 76 | 308 | – | Ref.[140] |
| ZrNBr | 0 | 133 | 69 | Ref.[131] |
| ZrNI | 0 | 74 | 116 | Ref.[131] |
| $Zr_2Te_2$ | 26 | 137 | – | Ref.[140] |
| $ZrS_2$ | 41 | 83 | 130 | Ref.[131] |
| $ZrSe_2$ | 39 | 111 | 230 | Ref.[131] |

**Supplementary table 7 | Semiconducting materials: Theoretical mobilities.** Self-reliance index ($S_C$) and theoretical hole and electron carrier mobilities for semiconducting single crystals. Strategic materials, as outlined in the main text, are highlighted blue in the table.

**Supplementary Table 8** | Batteries: Lithium-ion electrodes

| | Element/Compound | Self-reliance, $S_E$ | Self-reliance, $S_C$ | Theor. Capacity (mAh $g^{-1}$) | Reference |
|---|---|---|---|---|---|
| **Elemental capacity** | Ag | 72 | - | 248 | Ref.[200] |
| | Al | 17 | - | 993 | Ref.[200] |
| | As | 41 | - | 1073 | Ref.[200] |
| | Au | 100 | - | 510 | Ref.[200] |
| | Bi | 16 | - | 385 | Ref.[200] |
| | Br | 0 | - | 335 | Ref.[200] |
| | Ca | 100 | - | 1340 | Ref. [201] |
| | Cd | 89 | - | 238 | Ref.[200] |
| | Cl | 100 | - | 756 | Ref.[202] |
| | F | 29 | - | 1410 | Ref.[202] |
| | Ga | 1 | - | 769 | Ref.[200] |
| | Ge | 14 | - | 1384 | Ref.[200] |
| | Graphite | 2 | - | 372 | Ref.[200] |
| | I | 0 | - | 211 | Ref.[200] |
| | In | 75 | - | 1012 | Ref.[200] |
| | Li | 0 | - | 3861 | Ref.[200] |
| | Mg | 0 | - | 195 | Ref.[200] |
| | P | 13 | - | 2596 | Ref.[200] |
| | Pb | 94 | - | 550 | Ref.[200] |
| | S | 100 | - | 1675 | Ref.[200] |
| | Sb | 11 | - | 660 | Ref.[200] |
| | Se | 92 | - | 678 | Ref.[200] |
| | Si | 29 | - | 3579 | Ref.[200] |
| | Sn | 100 | - | 960 | Ref.[200] |
| | Te | 100 | - | 420 | Ref.[200] |
| | Zn | 58 | - | 410 | Ref.[200] |
| | | | | | |
| **Anode Material** | $Fe_3O_4$ | - | 35 | 926 | Ref.[203] |
| | α-$Fe_2O_3$ | - | 35 | 1007 | Ref.[204] |
| | TiNbO | - | 0 | 402 | Ref.[203] |
| | Si | - | 29 | 4212 | Ref.[204] |
| | $TiO_2$ | - | 1 | 330 | Ref.[204] |
| | $LiTi_4O_5$ | - | 0 | 175 | Ref.[204] |
| | Ge | - | 14 | 1624 | Ref.[204] |
| | Sn | - | 100 | 993 | Ref.[204] |
| | Sb | - | 11 | 660 | Ref.[204] |
| | SiO | - | 29 | 1600 | Ref.[204] |
| | $SnO_2$ | - | 100 | 783 | Ref.[204] |
| | CNTs | - | 100 | 1116 | Ref.[204] |
| | Graphite | - | 2 | 372 | Ref.[204] |
| | Graphene | - | 100 | 1116 | Ref.[204] |
| | $Co_3O_4$ | - | 16 | 890 | Ref.[204] |
| | CoO | - | 16 | 715 | Ref.[204] |

| | | | | | |
|---|---|---|---|---|---|
| Cathode Material | $LiTiS_2$ | - | 0 | 225 | Ref.[200] |
| | LCO | - | 0 | 274 | Ref.[200] |
| | LMO | - | 0 | 285 | Ref.[200] |
| | $Li_2MnO_3$ | - | 0 | 458 | Ref.[200] |
| | $LiFeSO_4F$ | - | 0 | 151 | Ref.[200] |
| | NCM | - | 19 | 280 | Ref.[200] |
| | NCA | - | 25 | 279 | Ref.[200] |
| | LCP | - | 0 | 167 | Ref.[200] |
| | LFP | - | 0 | 170 | Ref.[200] |
| | S | - | 100 | 1675 | Ref.[205] |
| | $FeF_3$ | - | 30 | 713 | Ref.[205] |
| | $NiS_2$ | - | 65 | 870 | Ref.[206] |
| | $MnCl_2$ | - | 8 | 425 | Ref.[205] |
| | $FeCl_3$ | - | 35 | 495 | Ref.[205] |
| | $TiF_3$ | - | 12 | 765 | Ref.[205] |
| | $MnS_2$ | - | 43 | 830 | Ref.[205] |
| | $Li_2S$ | - | 0 | 1166 | Ref.[205] |
| | PTMA 68 | - | 100 | 239 | Ref.[207] |
| | NBHQ 113 | - | 100 | 489 | Ref.[207] |
| | PS 120 | - | 100 | 315 | Ref.[207] |
| | PTGE 70 | - | 100 | 147 | Ref.[207] |

**Supplementary table 8 | Batteries: Lithium-ion electrodes.** Theoretical capacity**,** elemental ($S_E$) and compound ($S_C$) self-reliance indexes for lithium-ion battery electrode materials.

**Supplementary Table 9** | Batteries: Sodium-ion electrodes

| | Compound | Self-reliance, $S_C$ | Theoretical Capacity (mAh $g^{-1}$) | Reference |
|---|---|---|---|---|
| **Anode Materials** | Hard carbon | 100 | 305 | Ref. [208] |
| | Sn | 100 | 847 | Ref.[209] |
| | Sb | 11 | 660 | Ref.[210] |
| | P | 13 | 2596 | Ref.[211] |
| | $Na_2Ti_3O_7$ | 1 | 177 | Ref.[212] |
| | $TiO_2$ | 1 | 335 | Ref.[213] |
| | Graphite | 2 | 35 | Ref.[214] |
| | Boron–graphene ($BC_3$) | 0 | 762 | Ref.[215] |
| | $MoO_3$ | 14 | 1117 | Ref.[216] |
| | $Mo_2C$ | 14 | 132 | Ref.[216] |
| | MoC | 14 | 248 | Ref.[216] |
| | $Na_{15}Sn_4$ | 100 | 847 | Ref.[217] |
| | $Na_3Sb$ | 11 | 660 | Ref.[217] |
| | $Sb_2S_3$ | 41 | 946 | Ref.[217] |
| | α-Si | 29 | 725 | Ref.[218] |
| | Ge | 14 | 396 | Ref.[218] |
| | Bi | 16 | 385 | Ref.[218] |
| | Pb | 94 | 485 | Ref.[218] |
| | In | 75 | 467 | Ref.[218] |
| **Cathode Materials** | $NaNiO_2$ | 57 | 250 | Ref.[219] |
| | $Na_3V_2(PO_4)_3$ | 8 | 118 | Ref.[220] |
| | $Na_2FeP_2O_7$ | 18 | 97 | Ref.[221] |
| | $Na_2C_6O_6$ | 100 | 501 | Ref.[222] |
| | $Na_7Fe_7(PO_4)_6F_3$ | 23 | 159 | Ref.[223] |
| | C4Q, P5Q | 100 | 446 | Ref.[222] |
| | PTCDA | 100 | 137 | Ref.[222] |
| | PBQS No. 8 | 100 | 388 | Ref.[222] |
| | PDPPD No. 14 | 100 | 209 | Ref.[222] |
| | TABQ-COF | 100 | 515 | Ref.[222] |
| | PBAs | 100 | 170 | Ref.[224] |

**Supplementary table 9 | Batteries: Sodium-ion electrodes.** Theoretical capacity and compound self-reliance indexes ($S_C$) for sodium-ion battery electrode materials.

**Supplementary Table 10** | Batteries: Potassium-ion electrodes

| | Compound | Self-reliance, $S_C$ | Theoretical Capacity (mAh $g^{-1}$) | Reference |
|---|---|---|---|---|
| Anode Materials | Graphite | 2 | 279 | Ref.[214] |
| | RGO:S | 100 | 361 | Ref.[225] |
| | Sb | 11 | 660 | Ref.[226] |
| | $K_2Ti_8O_{17}$ | 2 | 308 | Ref.[227] |
| | GeSe | 36 | 530 | Ref.[228] |
| | Graphene:N | 100 | 350 | Ref.[229] |
| | $KTiOPO_4$ | 10 | 192.3 | Ref.[229] |
| | Bi | 16 | 385 | Ref.[229] |
| | P | 13 | 843 | Ref.[230] |
| | Sn | 100 | 225 | Ref.[218] |
| | $SnS_2$ | 100 | 733 | Ref.[231] |
| | $Sn_4P_3$ | 42 | 612 | Ref.[232] |
| | $WS_2$ | 91 | 431.6 | Ref.[231] |
| | VK | 100 | 313.5 | Ref.[231] |
| | | | | |
| Cathode Materials | Prussian white | 100 | 155 | Ref.[233] |
| | $K_3V_2(PO_4)_3$ | 14 | 106 | Ref.[234] |
| | PAQS | 100 | 226 | Ref.[235] |
| | $KMoP_2O_7$ | 17 | 87 | Ref.[236] |
| | $KVP_2O_7$ | 13 | 102 | Ref.[236] |
| | $KFeSO_4F$ | 45 | 128 | Ref.[236] |
| | $K_2MnP_2O_7$ | 18 | 175 | Ref.[236] |
| | $\alpha$-$FePO_4$ | 21 | 178 | Ref.[236] |
| | KMnHCF | 21 | 156 | Ref.[229] |
| | KFeHCF | 34 | 170 | Ref.[229] |
| | PTCDI | 100 | 137 | Ref.[229] |
| | PQ-CN | 100 | 184 | Ref.[229] |
| | PDPPD | 100 | 209 | Ref.[229] |
| | Vitamin K | 100 | 313.5 | Ref.[237] |
| | $KFeMnO_2$ | 22 | 220 | Ref.[238] |
| | $K_4Fe_3(PO_4)_2(P_2O_7)$ | 23 | 120 | Ref.[238] |
| | OHTAP | 100 | 440 | Ref.[238] |
| | AQ | 100 | 257 | Ref.[239] |

**Supplementary table 10 | Batteries: Potassium-ion electrodes.** Theoretical capacity and compound self-reliance indexes ($S_C$) for potassium-ion battery electrode materials.

**Supplementary Table 11**| Photovoltaics: Hole transport materials (p-i-n)

| | Compound | Self-reliance, $S_C$ | PCE (%) | Reference |
|---|---|---|---|---|
| Hole Transport Layer (p-i-n) | 2PACz + Me-4PACz | 24 | 26.9 | Ref.[240] |
| | $NiO_X$ \| Me-4PACz | 24 | 24.93 | Ref.[241] |
| | MeO-2PACz | 24 | 24.8 | Ref.[242] |
| | PTAA | 100 | 23.0 | Ref.[243] |
| | PEDOT:PSS | 100 | 21.26 | Ref.[244] |
| | NiO-np \| PMMA | 25 | 22.13 | Ref.[245] |
| | Alkoxy-PTEG | 100 | 21.2 | Ref.[246] |
| | DBTMT | 100 | 21.12 | Ref.[247] |
| | MPA-BTTI | 100 | 21.17 | Ref.[248] |
| | TPE-S | 100 | 21.0 | Ref.[249] |
| | P3CT-N | 100 | 21.03 | Ref.[250] |
| | NiMgLiO | 0 | 19.2 | Ref.[251] |
| | In:$CuCrO_2$ | 77 | 20.53 | Ref.[252] |
| | DBFMT | 100 | 20.51 | Ref.[253] |
| | LiNiO-c | 0 | 20.5 | Ref.[254] |
| | TFB \| $Al_2O_3$-np | 17 | 18.48 | Ref.[255] |
| | 2PACz | 24 | 20.9 | Ref.[256] |
| | P3CT-Na | 100 | 20.71 | Ref.[257] |
| | CzPAF-TPA | 100 | 15.71 | Ref.[258] |
| | PolyTPD \| PFN | 0 | 20.0 | Ref.[259] |
| | NPB | 100 | 19.96 | Ref.[260] |
| | PASQ-IDT | 100 | 18.29 | Ref.[261] |
| | TB(MA) | 100 | 19.76 | Ref.[262] |
| | DFH | 100 | 20.6 | Ref.[263] |
| | $MoO_3$ \| TaTm | 14 | 19.3 | Ref.[264] |
| | BDT-POZ | 0 | 19.16 | Ref.[265] |
| | $NiCo_2O_4$ | 24 | 19.24 | Ref.[266] |
| | $CuCrO_2$ | 78 | 19.0 | Ref.[267] |
| | $CuO_x$ | 71 | 19.0 | Ref.[268] |
| | Trux-OMeTAD | 100 | 18.6 | Ref.[269] |
| | DFBT-MTP | 54 | 20.2 | Ref.[270] |

**Supplementary table 11 | Photovoltaics: Hole transport materials (p-i-n).** Power conversion efficiency (*PCE*) and compound self-reliance indexes ($S_C$) for hole transport materials in a p-i-n solar cell configuration.

**Supplementary Table 12** | Photovoltaics: Hole transport materials (n-i-p)

| | Compound | Self-reliance, $S_C$ | PCE (%) | Reference |
|---|---|---|---|---|
| **Hole Transport Layer (n-i-p)** | Spiro-MeOTAD doped with Co-TFSI; Li-TFSI;TBP | 0 | 25.2 | Ref.[271] |
| | Spiro-MeOTAD doped with Li-TFSI and TBP | 0 | 23.24 | Ref.[272] |
| | Spiro-MeOTAD doped with Zn-TFSI and TBP | 55 | 22.0 | Ref.[273] |
| | Spiro-MeOTAD doped with PFPPY | 100 | 21.38 | Ref.[274] |
| | P3HT | 100 | 23.3 | Ref.[275] |
| | DM | 100 | 23.2 | Ref.[276] |
| | PTAA | 100 | 22.6 | Ref.[277] |
| | WT3 | 58 | 19.44 | Ref.[278] |
| | DBC-OMeDPA | 100 | 22.0 | Ref.[279] |
| | DCZ-OMeTAD | 100 | 21.66 | Ref.[280] |
| | PTA | 31 | 17.0 | Ref.[281] |
| | PDCBT \| $WO_X$ | 87 | 21.2 | Ref.[282] |
| | PHPT-py \| Rutin–AgNP | 85 | 21. | Ref.[283] |
| | T5H-OMeDPA | 100 | 21.1 | Ref.[284] |
| | O5H-OMeDPA | 100 | 20.03 | Ref.[285] |
| | mDPA-DBTP | 100 | 18.09 | Ref.[286] |
| | SFXDAnCBZ | 100 | 20.87 | Ref.[287] |
| | PDCBT \| Ta-$WO_x$ | 20 | 20.8 | Ref.[288] |
| | X59 | 100 | 19.8 | Ref.[289] |
| | OMe-TATPyr | 100 | 20.6 | Ref.[290] |
| | PCDTBT1 | 100 | 20.9 | Ref.[291] |
| | Co(II)P; Co(III)P | 16 | 20.47 | Ref.[292] |
| | DTP-C6Th | 100 | 20.36 | Ref.[293] |
| | CuSCN | 84 | 20.3 | Ref.[294] |
| | P3 | 35 | 20.3 | Ref.[295] |
| | DTB(xDEG) | 100 | 20.19 | Ref.[296] |
| | PDO2 | 100 | 20.2 | Ref.[297] |
| | PBDT(2F)T | 100 | 17.52 | Ref.[298] |
| | TFAP | 100 | 19.74 | Ref.[299] |
| | OMe-TPA-CuPc | 71 | 19.67 | Ref.[300] |
| | SnS | 100 | 13.7 | Ref.[301] |
| | PET-OMeDPA | 100 | 19.8 | Ref.[284] |
| | Co-Porphyrin | 16 | 18.3 | Ref.[302] |
| | PTEG | 100 | 19.8 | Ref.[303] |
| | DTPC8-ThDTPA | 100 | 19.42 | Ref.[304] |
| | $NiO_X$ | 57 | 21.59 | Ref.[305] |

**Supplementary table 12** | **Photovoltaics: Hole transport materials (n-i-p).** Power conversion efficiency (*PCE*) and compound self-reliance indexes ($S_C$) for hole transport materials in a n-i-p solar cell configuration.

**Supplementary Table 13** | Photovoltaics: Electron transport materials (p-i-n)

| | Material | Self-reliance, $S_C$ | PCE (%) | Reference |
|---|---|---|---|---|
| Electron Transport Layer (p-i-n) | C60 \| $SnO_2$ | 100 | 26.9 | Ref.[240] |
| | PCBM-60 \| $SnO_2$ | 100 | 24.93 | Ref.[241] |
| | PCBM-60 \| BCP | 100 | 24.8 | Ref.[242] |
| | C60 \| BCP | 100 | 23.0 | Ref.[243] |
| | PCBM-60 | 100 | 21.43 | Ref.[306] |
| | ZnO-mp \| ZnO-np | 58 | 21.11 | Ref.[307] |
| | NDI-PhE | 100 | 20.5 | Ref.[308] |
| | NDI-ID (RR) | 100 | 20.4 | Ref.[309] |
| | NDI-PM | 100 | 19.6 | Ref.[310] |
| | C70-DPM-OE | 100 | 16.0 | Ref.[311] |
| | $CeO_x$ | 0 | 17.1 | Ref.[312] |
| | IT4M \| s-Bphen | 100 | 17.65 | Ref.[313] |
| | ITCPTC-Th \| Rhodamine 101 | 100 | 17.05 | Ref.[314] |
| | $Nb_2O_5$ | 0 | 19.5 | Ref.[315] |
| | NDI-BTH2 | 100 | 15.4 | Ref.[316] |
| | NDIF1 | 100 | 15.6 | Ref.[317] |
| | P(NDI2OD-T2) | 100 | 18.83 | Ref.[318] |
| | $SiO_2$-c \|Ga- ZnO | 12 | 18.4 | Ref.[319] |
| | TDTP | 100 | 18.2 | Ref.[320] |

**Supplementary table 13** | **Photovoltaics: Electron transport materials (p-i-n).** Power conversion efficiency (*PCE*) and compound self-reliance indexes ($S_C$) for electron transport materials in a p-i-n solar cell configuration.

**Supplementary Table 14** | Photovoltaics: Electron transport materials (n-i-p)

| | Material | Self-reliance, $S_C$ | PCE (%) | Reference |
|---|---|---|---|---|
| Electron Transport Layer (n-i-p) | $SnO_2$ | 100 | 25.7 | Ref.[321] |
| | $TiO_2$-c \| $TiO_2$-mp | 1 | 24.82 | Ref.[322] |
| | $In_2O_3$-c | 75 | 19.21 | Ref.[272] |
| | Al:ITO-c | 56 | 17.2 | Ref.[323] |
| | AZO-c | 31 | 17.6 | Ref.[324] |
| | $Ba(OH)_2$ | 16 | 18.18 | Ref.[325] |
| | BCP | 100 | 19.07 | Ref.[326] |
| | C60 | 100 | 20.4 | Ref.[327] |
| | CDIN | 100 | 17.12 | Ref.[328] |
| | CdS | 94 | 16.1 | Ref.[329] |
| | $CeO_x$\| PCBM | 0 | 17.04 | Ref.[330] |
| | CPTA | 100 | 18.39 | Ref.[331] |
| | $Cr_2O_3$ | 85 | 16.23 | Ref.[332] |
| | EDTA | 100 | 16.42 | Ref.[333] |
| | Ti-$Fe_2O_3$ | 11 | 17.85 | Ref.[334] |
| | IDIC | 100 | 19.1 | Ref.[335] |
| | IZO | 69 | 16.25 | Ref.[336] |
| | $Nb_2O_5$ | 0 | 20.22 | Ref.[337] |
| | $NbO_x$ | 0 | 19.09 | Ref.[338] |
| | nTi-MOF | 1 | 16.41 | Ref.[339] |
| | PCBCB | 100 | 18.8 | Ref.[340] |
| | $SnS_2$ | 100 | 20.12 | Ref.[341] |
| | $SrGeO_3$ | 37 | 17.68 | Ref.[342] |
| | $SrSnO_3$:Y | 51 | 19.0 | Ref.[343] |
| | $TiS_2$ | 22 | 18.79 | Ref.[344] |
| | TMAH | 100 | 20.1 | Ref.[345] |
| | $WO_X$ | 75 | 20.77 | Ref.[346] |
| | ZnO-c | 58 | 20.44 | Ref.[347] |

**Supplementary table 14 | Photovoltaics: Electron transport materials (n-i-p).** Power conversion efficiency (*PCE*) and compound self-reliance indexes ($S_C$) for electron transport materials in a n-i-p solar cell configuration.

**Supplementary Table 15** | Photovoltaics: Absorption layers

| | Compound | Self-reliance, $S_C$ | PCE (%) | Reference |
|---|---|---|---|---|
| Perovskites | $Cs_{0.05}MA_{0.1}FA_{0.85}PbI_3$ | 0 | 26.9 | Ref.[240] |
| | $FA_{0.992}MA_{0.008}PbBr_{0.024}I_{2.976}$ | 0 | 25.4 | Ref.[271] |
| | $Cs_{0.05}FA_{0.79}MA_{0}.17PbBr_{0.51}I_{2.49}$ | 0 | 24.8 | Ref.[242] |
| | $FA_{0.93}MA_{0}.02Cs_{0}.05PbI_{2.94}Br_{0.06}$ | 0 | 24.93 | Ref.[241] |
| | $FAPbI_3$ | 0 | 24.66 | Ref.[348] |
| | $Cs_{0.1}FA_{0.9}PbI$ | 0 | 23.21 | Ref.[349] |
| | $MAPbI_3$ | 0 | 21.8 | Ref.[350] |
| | $CsPbBrI_2$ | 0 | 19.02 | Ref.[351] |
| | $CsPbI_3$ | 0 | 19.03 | Ref.[352] |
| | $FAPbBr_3$ | 0 | 19.3 | Ref.[353] |
| | $CsPbI_2Br$ | 0 | 14 | Ref.[354] |
| Iodine/bromine free perovskites | $FAPbCl_3$ | 94 | 8.75 | Ref.[355] |
| | $MASnCl_3$ | 100 | 10.46 | Ref.[356] |
| | $FA_4GeSbCl_{12}$ | 12 | 4.7 | Ref.[357] |
| | $MA_2NaBiCl_6$ | 16 | 2.09 | Ref.[358] |
| | $MAAlCl_4$ | 17 | 0.96 | Ref.[359] |
| | $SrTiO_3$ | 10 | 0.88 | Ref.[360] |
| Organics | D18-Cl (2% CN):BTP4F-P2EH (0.5% CN) | 54 | 20.8 | Ref.[361] |
| | D18:L8-BO with 0.5% B6Cl | 0 | 20.2 | Ref.[362] |
| | PBDB-TF:L8-BO:BTP-eC9 | 54 | 20.17 | Ref.[363] |
| | D18:20%PM6:L8-BO | 81 | 19.9 | Ref.[364] |
| | PM6:BTP-C9:o-BTP-eC9 | 100 | 19.88 | Ref.[365] |
| | PM6:BTP-eC9:BTP-S16:BTP-S17 | 54 | 19.76 | Ref.[366] |
| Dye-synthesised | $TiO_2$ - SL9+SL10/BPHA | 10 | 15.2 | Ref.[367] |
| | $TiO_2$ - ADEKA-1+LEG4 | 5 | 14.3 | Ref.[368] |
| | SGT021+SGT-149 | 25 | 14.2 | Ref.[369] |
| | ZS4 | 10 | 13.2 | Ref.[370] |
| | SGT-021 | 18 | 13.17 | Ref.[371] |
| | Black dye + Y1 | 0 | 11.4 | Ref.[372] |
| | XW40 | 18 | 10.6 | Ref.[373] |
| Ferroelectrics | $0.5Ba(Zr_{0.2}Ti_{0.8})O_3$-$0.5(Ba_{0.7}Ca_{0.3})TiO_3$ | 8 | 10.9 | Ref.[374] |
| | $Bi_2FeCrO$ | 30 | 8.1 | Ref.[375] |
| | $Bi_{0.95}La_{0.05}FeO_3$ | 0 | 5.62 | Ref.[376] |
| | $BaTiO_3$ | 4 | 4.8 | Ref.[377] |
| | $PbTiO_3$ | 10 | 4.2 | Ref.[378] |
| | $BiFeO_3$ | 24 | 4 | Ref.[379] |
| | $PbZrTiO_3$ | 9 | 2.49 | Ref.[380] |
| Other thin films | GaAs | 6 | 29.1 | Ref.[381] |
| | CdTe | 94 | 23.08 | Ref.[382] |
| | CdTe:Se | 94 | 22.1 | Ref.[383] |
| | CdS:CdTe | 94 | 15.8 | Ref.[383] |

| | | | | |
|---|---|---|---|---|
| | CIGS | 9 | 23.6 | Ref.[382] |
| | $Cu_2O$ | 71 | 5 | Ref.[379] |
| | $Cu_2O$:Na | 71 | 8 | Ref.[379] |

**Supplementary table 15 | Photovoltaics: Absorption layers.** Power conversion efficiency (*PCE*) and compound self-reliance indexes ($S_C$) for absorption layers across a range of photovoltaic technologies.

**Supplementary Note 3** | List of Strategic Nanomaterials

The following nanomaterials shall be considered strategic:

| Application | Critical performance indicator | Nanomaterial |
|---|---|---|
| Semiconductors: Single crystal | $\mu_C$ = 41 cm$^2$ V$^{-1}$ s$^{-1}$ | CNTs<br>$HfS_2$<br>InSe<br>$In_2O_3$<br>Rubrene<br>$SnO_2$<br>$SnS_2$<br>SnSe<br>$SnSe_2$<br>$WS_2$<br>$WSe_2$<br>$WTe_2$<br>$ZnSnO_3$ |
| Semiconductors: Thin films | $\mu_C$ = 14 cm$^2$ V$^{-1}$ s$^{-1}$ | CNTs<br>InO<br>InSe<br>ISZO<br>PbSe<br>$SnS_2$<br>$SnSe_2$<br>$WS_2$<br>$WSe_2$ |
| Batteries: Elemental capacity | $C_C$ = 725 mAh g$^{-1}$ | Ca<br>Cl<br>In<br>S<br>Sn |
| Batteries: Lithium ion | $C_C$ = 534 mAh g$^{-1}$ | CNTs<br>Graphene<br>$NiS_2$<br>S<br>Sn<br>$SnO_2$ |
| Batteries: Sodium ion | $C_C$ = 355 mAh g$^{-1}$ | C4Q, P5Q<br>In<br>Na15Sn4<br>Pb<br>PBQS No. 8<br>TABQ-COF |
| Batteries: Potassium ion | $C_C$ = 249 mAh g$^{-1}$ | AQ<br>Graphene:N<br>OHTAP<br>RGO:S<br>$SnS_2$<br>Vitamin K |

| | | | |
|---|---|---|---|
| | | | VK |
| | | | $WS_2$ |
| Photovoltaics: Hole transport layers | $PCE_C$ = 20.4 % | p-i-n | Alkoxy-PTEG |
| | | | DBTMT |
| | | | DFH |
| | | | In:$CuCrO_2$ |
| | | | MPA-BTTI |
| | | | P3CT-N |
| | | | P3CT-Na |
| | | | PEDOT:PSS |
| | | | PTAA |
| | | | TPE-S |
| | | n-i-p | DBC-OMeDPA |
| | | | DCZ-OMeTAD |
| | | | DM |
| | | | OMe-TATPyr |
| | | | P3HT |
| | | | PCDTBT1 |
| | | | PDCBT \| $WO_X$ |
| | | | PHPT-py \| Rutin-Ag |
| | | | PTAA |
| | | | SFXDAnCBZ |
| | | | Spiro:PFPPY |
| Photovoltaics: Electron transport layers | $PCE_C$ = 19.1 % | p-i-n | C60 \| BCP |
| | | | C60 \| $SnO_2$ |
| | | | NDI-ID (RR) |
| | | | NDI-PhE |
| | | | PCBM-60 |
| | | | PCBM-60 \| BCP |
| | | | PCBM-60 \| $SnO_2$ |
| | | n-i-p | C60 |
| | | | IDIC |
| | | | $In_2O_3$-c |
| | | | $SnO_2$ |
| | | | $SnS_2$ |
| Photovoltaics: Light-absorbing layers | $PCE_C$ = 12.8 % | | CdTe |
| | | | CdTe:Se |
| | | | CdS:CdTe |
| | | | D18:20%PM6:L8-BO |
| | | | PM6:BTP-C9:o-BTP-eC9 |

## Supplementary References


1. Jakub Jirasek MS, Petr Laznicka. *Mineral Deposits*. Anagram (2017).

2. Miloš Kužvart ZM, František Patočka, Zdeněk Pertold, Zdeněk Pouba. *Mineral resources of the world: Ores and Industrial Minerals*, 2nd revised edition edn. Academia (1995).

3. Smirnov VI. *Geology of Mineral Deposits*. SNTL – Nakladatelství technické literatury (1982).

4. Daily Metal Price, Metal prices database, available at: https://www.dailymetalprice.com/ (accessed 23 March 2026).

5. Shanghai Metals Market (SMM), Metal.com, available at: https://www.metal.com/ (accessed 23 March 2026).

6. IMARC Group, Pricing reports, available at: https://www.imarcgroup.com/ (accessed 23 March 2026).

7. BOC UK, available at: https://www.boconline.co.uk/shop/en/uk/home (accessed 23 March 2026).

8. Business Analytiq, Procurement Analytics, available at: https://businessanalytiq.com/procurementanalytics/index (accessed 23 March 2026).

9. Haynes WM. *CRC Handbook of Chemistry and Physics*, 97 edn. CRC Press (2016).

10. Gogotsi YE. *MXenes: From Discovery to Applications of Two-Dimensional Metal Carbides and Nitrides*. Jenny Stanford Publishing (2023).

11. Zhang C*, et al.* Additive-free MXene inks and direct printing of micro-supercapacitors. *Nature Communications* **10**, (2019).

12. Chen H, Wen Y, Qi Y, Zhao Q, Qu L, Li C. Pristine Titanium Carbide MXene Films with Environmentally Stable Conductivity and Superior Mechanical Strength. *Advanced Functional Materials* **30**, (2019).

13. Zhang J*, et al.* Scalable Manufacturing of Free-Standing, Strong Ti3C2Tx MXene Films with Outstanding Conductivity. *Advanced Materials* **32**, (2020).

14. Torrisi F*, et al.* Inkjet-Printed Graphene Electronics. *ACS Nano* **6**, 2992-3006 (2012).

15. Cassidy O*, et al.* Layer-by-layer assembly yields thin graphene films with near theoretical conductivity. *npj 2D Materials and Applications* **9**, (2025).

16. Fernandes IJ*, et al.* Silver nanoparticle conductive inks: synthesis, characterization, and fabrication of inkjet-printed flexible electrodes. *Scientific Reports* **10**, (2020).

17. Jeong S, Song HC, Lee WW, Choi Y, Ryu B-H. Preparation of aqueous Ag Ink with long-term dispersion stability and its inkjet printing for fabricating conductive tracks on a polyimide film. *Journal of Applied Physics* **108**, (2010).

18. Williams NX, Noyce S, Cardenas JA, Catenacci M, Wiley BJ, Franklin AD. Silver nanowire inks for direct-write electronic tattoo applications. *Nanoscale* **11**, 14294-14302 (2019).

19. Coleman E*, et al.* Extracting the Temperature Dependence of Both Nanowire Resistivity and Junction Resistance from Electrical Measurements on Printed Silver Nanowire Networks. *ACS Applied Electronic Materials* **7**, 806-815 (2025).

20. Cui W, Lu W, Zhang Y, Lin G, Wei T, Jiang L. Gold nanoparticle ink suitable for electric-conductive pattern fabrication using in ink-jet printing technology. *Colloids and Surfaces A: Physicochemical and Engineering Aspects* **358**, 35-41 (2010).

21. Wu Y, Li Y, Liu P, Gardner S, Ong BS. Studies of Gold Nanoparticles as Precursors to Printed Conductive Features for Thin-Film Transistors. *Chemistry of Materials* **18**, 4627-4632 (2006).

22. Mani S, Saif T, Han JH. Effect of annealing on the conductivity of electroless deposited Ni nanowires and films. *IEEE Transactions On Nanotechnology* **5**, 138-141 (2006).

23. Lyons PE*, et al.* The relationship between network morphology and conductivity in nanotube films. *Journal of Applied Physics* **104**,  (2008).

24. Wu Z*, et al.* Transparent, Conductive Carbon Nanotube Films. *Science* **305**, 1273-1276 (2004).

25. Wang T*, et al.* Thermoelectric performance of restacked MoS2nanosheets thin-film. *Nanotechnology* **27**,  (2016).

26. Synnatschke K*, et al.* Inert Liquid Exfoliation and Langmuir-Type Thin Film Deposition of Semimetallic Metal Diborides. *ACS Nano* **18**, 28596-28608 (2024).

27. Kasap S, Koughia C, Ruda HE. Electrical conduction in metals and semiconductors. *Springer handbook of electronic and photonic materials*, 1-1 (2017).

28. George J, Menon CS. Electrical and optical properties of electron beam evaporated ITO thin films. *Surface and Coatings Technology* **132**, 45-48 (2000).

29. Yang JK, Liang B, Zhao MJ, Gao Y, Zhang FC, Zhao HL. Reference of Temperature and Time during tempering process for non-stoichiometric FTO films. *Scientific Reports* **5**,  (2015).

30. Kelly AG*, et al.* Highly Conductive Networks of Silver Nanosheets. *Small* **18**,  (2022).

31. Joo M, Lee B, Jeong S, Lee M. Comparative studies on thermal and laser sintering for highly conductive Cu films printable on plastic substrate. *Thin Solid Films* **520**, 2878-2883 (2012).

32. Robertson J. High dielectric constant oxides. *The European Physical Journal Applied Physics* **28**, 265-291 (2004).

33. Hoerman BH, Ford GM, Kaufmann LD, Wessels BW. Dielectric properties of epitaxial BaTiO3 thin films. *Applied Physics Letters* **73**, 2248-2250 (1998).

34. Robertson J. Band offsets of wide-band-gap oxides and implications for future electronic devices. *Journal of Vacuum Science & Technology B: Microelectronics and Nanometer Structures Processing, Measurement, and Phenomena* **18**, 1785-1791 (2000).

35. Nalawade Y*, et al.* All-Printed Dielectric Capacitors from High-Permittivity, Liquid-Exfoliated BiOCl Nanosheets. *ACS Applied Electronic Materials* **2**, 3233-3241 (2020).

36. Lu S*, et al.* Flexible, Print-in-Place 1D–2D Thin-Film Transistors Using Aerosol Jet Printing. *ACS Nano* **13**, 11263-11272 (2019).

37. Sanchez-Duenas L*, et al.* A Review on Sustainable Inks for Printed Electronics: Materials for Conductive, Dielectric and Piezoelectric Sustainable Inks. *Materials* **16**, (2023).

38. Ayhan NK, Torgut G. Structural, Dielectric, and Electrical Properties of Chitosan Composites Doped with Silver for Electrochemical Applications. *ChemistrySelect* **9**, (2024).

39. Bekin S, Sarmad S, Gürkan K, Yenici G, Keçeli G, Gürdağ G. Dielectric, thermal, and swelling properties of calcium ion-crosslinked sodium alginate film. *Polymer Engineering & Science* **54**, 1372-1382 (2014).

40. Yang X, Yang W, Hu J. Preparation of Low-Dielectric-Constant Kaolin Clay Ceramics by Chemical Cleaning Method. *Frontiers in Materials* **8**, (2021).

41. Zhu J*, et al.* Layer-by-Layer Assembled 2D Montmorillonite Dielectrics for Solution-Processed Electronics. *Advanced Materials* **28**, 63-68 (2015).

42. Low CG, Zhang Q. Ultra-thin and Flat Mica as Gate Dielectric Layers. *Small* **8**, 2178-2183 (2012).

43. Mania E*, et al.* Spontaneous doping on high quality talc-graphene-hBN van der Waals heterostructures. *2D Materials* **4**, (2017).

44. Osada M*, et al.* High-κ Dielectric Nanofilms Fabricated from Titania Nanosheets. *Advanced Materials* **18**, 1023-1027 (2006).

45. Illarionov YY*, et al.* Insulators for 2D nanoelectronics: the gap to bridge. *Nature Communications* **11**, (2020).

46. Zhong M*, et al.* Thickness-Dependent Carrier Transport Characteristics of a New 2D Elemental Semiconductor: Black Arsenic. *Advanced Functional Materials* **28**, (2018).

47. Yang Z, Wu Z, Lyu Y, Hao J. Centimeter-scale growth of two-dimensional layered high-mobility bismuth films by pulsed laser deposition. *InfoMat* **1**, 98-107 (2019).

48. Lin Y-C*, et al.* A study on the epitaxial Bi2Se3 thin film grown by vapor phase epitaxy. *AIP Advances* **6**, (2016).

49. Locatelli L, Kumar A, Tsipas P, Dimoulas A, Longo E, Mantovan R. Magnetotransport and ARPES studies of the topological insulators Sb2Te3 and Bi2Te3 grown by MOCVD on large-area Si substrates. *Scientific Reports* **12**, (2022).

50. Chung D-Y*, et al.* CsBi4Te6: A High-Performance Thermoelectric Material for Low-Temperature Applications. *Science* **287**, 1024-1027 (2000).

51. Lee KH, Yu HS, Kim S-I, Moon SP, Hwang J-Y, Kim SW. Correlation between thermoelectric transport properties and crystal structure in two-dimensional CrSiTe3. *Journal of Alloys and Compounds* **790**, 93-98 (2019).

52. Late DJ*, et al.* GaS and GaSe Ultrathin Layer Transistors. *Advanced Materials* **24**, 3549-3554 (2012).

53. Sutter E*, et al.* Single Crystalline GeSe Van Der Waals Ribbons With Uniform Layer Stacking, High Carrier Mobility, and Adjustable Edge Morphology. *Small* **20**, (2024).

54. Di Bartolomeo A*, et al.* Field emission from two-dimensional GeAs. *Journal of Physics D: Applied Physics* **54**, (2020).

55. Kim D*, et al.* Thickness-dependent bandgap and electrical properties of GeP nanosheets. *Journal of Materials Chemistry A* **7**, 16526-16532 (2019).

56. Kanazawa T*, et al.* Few-layer HfS2 transistors. *Scientific Reports* **6**, (2016).

57. Mleczko MJ*, et al.* HfSe2 and ZrSe2: Two-dimensional semiconductors with native high-κ oxides. *Science Advances* **3**, (2017).

58. Lipatov A*, et al.* Electronic transport and polarization-dependent photoresponse in few-layered hafnium trisulfide (HfS3) nanoribbons. *Journal of Materials Chemistry C* **11**, 9425-9437 (2023).

59. Island JO, Blanter SI, Buscema M, van der Zant HSJ, Castellanos-Gomez A. Gate Controlled Photocurrent Generation Mechanisms in High-Gain In2Se3 Phototransistors. *Nano Letters* **15**, 7853-7858 (2015).

60. Bandurin DA*, et al.* High electron mobility, quantum Hall effect and anomalous optical response in atomically thin InSe. *Nature Nanotechnology* **12**, 223-227 (2016).

61. Wang J*, et al.* High Mobility MoS2 Transistor with Low Schottky Barrier Contact by Using Atomic Thick h-BN as a Tunneling Layer. *Advanced Materials* **28**, 8302-8308 (2016).

62. Kelly AG*, et al.* All-printed thin-film transistors from networks of liquid-exfoliated nanosheets. *Science* **356**, 69-73 (2017).

63. Zheng W, Bonn M, Wang HI. Photoconductivity Multiplication in Semiconducting Few-Layer MoTe2. *Nano Letters* **20**, 5807-5813 (2020).

64. Balendhran S*, et al.* Enhanced Charge Carrier Mobility in Two-Dimensional High Dielectric Molybdenum Oxide. *Advanced Materials* **25**, 109-114 (2012).

65. Liu H*, et al.* Vapor Deposition of Magnetic Van der Waals NiI2 Crystals. *ACS Nano* **14**, 10544-10551 (2020).

66. Liu J*, et al.* NiPS3 nanoflakes: a nonlinear optical material for ultrafast photonics. *Nanoscale* **11**, 14383-14391 (2019).

67. Li L*, et al.* Black phosphorus field-effect transistors. *Nature Nanotechnology* **9**, 372-377 (2014).

68. Jo HS, Oh GH, Kim S-i, Kim Tw. Atomically thin PdS2: physical characteristics and electronic device applications. *Journal of the Korean Physical Society* **83**, 751-755 (2023).

69. Lee B*, et al.* Fabrication of a Field-Effect Transistor Based on 2D Novel Ternary Chalcogenide PdPS. *ACS Applied Materials & Interfaces* **15**, 42891-42899 (2023).

70. Bafekry A*, et al.* Puckered Penta-like PdPX (X = O, S, Te) Semiconducting Nanosheets: First-Principles Study of the Mechanical, Electro-Optical, and Photocatalytic Properties. *ACS Applied Materials & Interfaces* **14**, 21577-21584 (2022).

71. Wang Z*, et al.* A Noble Metal Dichalcogenide for High-Performance Field-Effect Transistors and Broadband Photodetectors. *Advanced Functional Materials* **30**, (2019).

72. Zhao Y*, et al.* High-Electron-Mobility and Air-Stable 2D Layered PtSe2 FETs. *Advanced Materials* **29**, (2016).

73. Corbet CM, McClellan C, Rai A, Sonde SS, Tutuc E, Banerjee SK. Field Effect Transistors with Current Saturation and Voltage Gain in Ultrathin ReS2. *ACS Nano* **9**, 363-370 (2014).

74. Yang S*, et al.* Layer-dependent electrical and optoelectronic responses of ReSe2 nanosheet transistors. *Nanoscale* **6**, (2014).

75. Chen C*, et al.* Characterization of basic physical properties of Sb2Se3 and its relevance for photovoltaics. *Frontiers of Optoelectronics* **10**, 18-30 (2017).

76. Huang S-M*, et al.* Extremely high-performance visible light photodetector in the Sb2SeTe2 nanoflake. *Scientific Reports* **7**, (2017).

77. Ahmad M*, et al.* Engineering Interfacial Effects in Electron and Phonon Transport of Sb2Te3/MoS2 Multilayer for Thermoelectric ZT Above 2.0. *Advanced Functional Materials* **32**, (2022).

78. Kim D, Park K, Lee JH, Kwon IS, Kwak IH, Park J. Anisotropic 2D SiAs for High-Performance UV–Visible Photodetectors. *Small* **17**, (2021).

79. Daeneke T*, et al.* Wafer-Scale Synthesis of Semiconducting SnO Monolayers from Interfacial Oxide Layers of Metallic Liquid Tin. *ACS Nano* **11**, 10974-10983 (2017).

80. Khan H*, et al.* Liquid metal-based synthesis of high performance monolayer SnS piezoelectric nanogenerators. *Nature Communications* **11**, (2020).

81. Song HS*, et al.* High-performance top-gated monolayer SnS2 field-effect transistors and their integrated logic circuits. *Nanoscale* **5**, (2013).

82. Yang S*, et al.* Highly-anisotropic optical and electrical properties in layered SnSe. *Nano Research* **11**, 554-564 (2017).

83. Guo C, Tian Z, Xiao Y, Mi Q, Xue J. Field-effect transistors of high-mobility few-layer SnSe2. *Applied Physics Letters* **109**, (2016).

84. Zhu J*, et al.* Improving the thermoelectric properties of septuple atomic-layer SnBi2Se4 by regulating the carrier concentration through Nb doping. *Applied Physics Letters* **126**, (2025).

85. Li Y*, et al.* Chemical Vapor Deposition Growth of Atomically Thin SnSb2Te4 Single Crystals Toward Fast Photodetection. *Advanced Functional Materials* **34**, (2024).

86. Island JO*, et al.* Titanium trisulfide (TiS3): a 2D semiconductor with quasi-1D optical and electronic properties. *Scientific Reports* **6**, (2016).

87. Sucharitakul S*, et al.* V2O5: A 2D van der Waals Oxide with Strong In-Plane Electrical and Optical Anisotropy. *ACS Applied Materials & Interfaces* **9**, 23949-23956 (2017).

88. Cui Y*, et al.* High-Performance Monolayer WS2 Field-Effect Transistors on High-κ Dielectrics. *Advanced Materials* **27**, 5230-5234 (2015).

89. Liu W, Kang J, Sarkar D, Khatami Y, Jena D, Banerjee K. Role of Metal Contacts in Designing High-Performance Monolayer n-Type WSe2 Field Effect Transistors. *Nano Letters* **13**, 1983-1990 (2013).

90. Luo X*, et al.* Magnetoresistance and Hall resistivity of semimetal WTe2 ultrathin flakes. *Nanotechnology* **28**, (2017).

91. Daniel B, Agarwal KC, Lupaca-Schomber J, Klingshirn C, Hetterich M. Carrier concentration, mobility, and electron effective mass in chlorine-doped n-type Zn1−xMnxSe epilayers grown by molecular-beam epitaxy. *Applied Physics Letters* **87**, (2005).

92. Zhang L*, et al.* 2D organic single crystals: Synthesis, novel physics, high-performance optoelectronic devices and integration. *Materials Today* **50**, 442-475 (2021).

93. Galazka Z*, et al.* Experimental Hall electron mobility of bulk single crystals of transparent semiconducting oxides. *Journal of Materials Research* **36**, 4746-4755 (2021).

94. Balaghi L*, et al.* High electron mobility in strained GaAs nanowires. *Nature Communications* **12**, (2021).

95. Li D*, et al.* Ultra-fast photodetectors based on high-mobility indium gallium antimonide nanowires. *Nature Communications* **10**, (2019).

96. Yang Z-x*, et al.* Approaching the Hole Mobility Limit of GaSb Nanowires. *ACS Nano* **9**, 9268-9275 (2015).

97. Zhou Z, *et al.* Towards high-mobility In2xGa2–2xO3 nanowire field-effect transistors. *Nano Research* **11**, 5935-5945 (2018).

98. Ford AC, *et al.* Diameter-Dependent Electron Mobility of InAs Nanowires. *Nano Letters* **9**, 360-365 (2009).

99. Wang D, *et al.* Germanium nanowire field-effect transistors with SiO2 and high-κ HfO2 gate dielectrics. *Applied Physics Letters* **83**, 2432-2434 (2003).

100. Li Y, *et al.* Dopant-Free GaN/AlN/AlGaN Radial Nanowire Heterostructures as High Electron Mobility Transistors. *Nano Letters* **6**, 1468-1473 (2006).

101. He J, *et al.* Solution-processed wafer-scale indium selenide semiconductor thin films with high mobilities. *Nature Electronics* **8**, 244-253 (2025).

102. Neilson J, *et al.* Production of Ultrathin and High-Quality Nanosheet Networks via Layer-by-Layer Assembly at Liquid–Liquid Interfaces. *ACS Nano* **18**, 32589-32601 (2024).

103. Carey T, *et al.* A Portfolio of Electrochemically Exfoliated Two-Dimensional Materials: From Crystals and Simulations to Electronic Inks and Circuits. (2024).

104. Alsaif MMYA, *et al.* High-Performance Field Effect Transistors Using Electronic Inks of 2D Molybdenum Oxide Nanoflakes. *Advanced Functional Materials* **26**, 91-100 (2015).

105. Veeralingam S, Badhulika S. 2D - SnSe2 nanoflakes on paper with 1D - NiO gate insulator based MISFET as multifunctional NIR photo switch and flexible temperature sensor. *Materials Science in Semiconductor Processing* **105**, (2020).

106. Fortunato E, *et al.* High field-effect mobility zinc oxide thin film transistors produced at room temperature. *Journal of Non-Crystalline Solids* **338-340**, 806-809 (2004).

107. Lee J, *et al.* High mobility ultra-thin crystalline indium oxide thin film transistor using atomic layer deposition. *Applied Physics Letters* **113**, (2018).

108. Kim Y-H, *et al.* Flexible metal-oxide devices made by room-temperature photochemical activation of sol–gel films. *Nature* **489**, 128-132 (2012).

109. Shiah Y-S, *et al.* Mobility–stability trade-off in oxide thin-film transistors. *Nature Electronics* **4**, 800-807 (2021).

110. Krasienapibal TS, Fukumura T, Hirose Y, Hasegawa T. Improved room temperature electron mobility in self-buffered anatase TiO2epitaxial thin film grown at low temperature. *Japanese Journal of Applied Physics* **53**, (2014).

111. Patel KJ, Panchal CJ, Kheraj VA, Desai MS. Growth, structural, electrical and optical properties of the thermally evaporated tungsten trioxide (WO3) thin films. *Materials Chemistry and Physics* **114**, 475-478 (2009).

112. Wang Z, Paik H, Chen Z, Muller DA, Schlom DG. Epitaxial integration of high-mobility La-doped BaSnO3 thin films with silicon. *APL Materials* **7**, (2019).

113. Li C, *et al.* Synthesis of Crystalline Black Phosphorus Thin Film on Sapphire. *Advanced Materials* **30**, (2018).

114. Han SS, *et al.* Horizontal-to-Vertical Transition of 2D Layer Orientation in Low-Temperature Chemical Vapor Deposition-Grown PtSe2 and Its Influences on Electrical Properties and Device Applications. *ACS Applied Materials & Interfaces* **11**, 13598-13607 (2019).

115. Rhyee JS, *et al.* High-Mobility Transistors Based on Large-Area and Highly Crystalline CVD-Grown MoSe2 Films on Insulating Substrates. *Advanced Materials* **28**, 2316-2321 (2016).

116. Ji HG, *et al.* Chemically Tuned p- and n-Type WSe2 Monolayers with High Carrier Mobility for Advanced Electronics. *Advanced Materials* **31**, (2019).

117. Yang X, *et al.* High Mobility Two-Dimensional Bismuth Oxyselenide Single Crystals with Large Grain Size Grown by Reverse-Flow Chemical Vapor Deposition. *ACS Applied Materials & Interfaces* **13**, 49153-49162 (2021).

118. Han CY, Tang WM, Lai P-T. High-mobility pentacene organic thin-film transistors achieved by reducing remote phonon scattering and surface-roughness scattering. *Applied Surface Science* **544**, (2021).

119. Paulraj I, *et al.* High Performance of Post-Treated PEDOT:PSS Thin Films for Thermoelectric Power Generation Applications. *ACS Applied Materials & Interfaces* **13**, 42977-42990 (2021).

120. Euvrard J, Gunawan O, Kahn A, Rand BP. From Amorphous to Polycrystalline Rubrene: Charge Transport in Organic Semiconductors Paralleled with Silicon. *Advanced Functional Materials* **32**, (2022).

121. Scuratti F, *et al.* Charge Transport in High-Mobility Field-Effect Transistors Based on Inkjet Printed Random Networks of Polymer Wrapped Single-Walled Carbon Nanotubes. *Advanced Functional Materials* **31**, (2020).

122. Unalan HE, *et al.* Zinc oxide nanowire networks for macroelectronic devices. *Applied Physics Letters* **94**, (2009).

123. Duan X, *et al.* High-performance thin-film transistors using semiconductor nanowires and nanoribbons. *Nature* **425**, 274-278 (2003).

124. Koskinen T, *et al.* Thermoelectric Characteristics of InAs Nanowire Networks Directly Grown on Flexible Plastic Substrates. *ACS Applied Energy Materials* **4**, 14727-14734 (2021).

125. Sugahara M, Kawai H, Yomogida Y, Maniwa Y, Okada S, Yanagi K. Ambipolar transistors based on random networks of WS2 nanotubes. *Applied Physics Express* **9**, (2016).

126. Balazs DM, *et al.* Electron Mobility of 24 cm2 V−1 s−1 in PbSe Colloidal-Quantum-Dot Superlattices. *Advanced Materials* **30**, (2018).

127. Talgorn E, *et al.* Supercrystals of CdSe Quantum Dots with High Charge Mobility and Efficient Electron Transfer to TiO2. *ACS Nano* **4**, 1723-1731 (2010).

128. Nugraha MI, *et al.* Rapid Photonic Processing of High-Electron-Mobility PbS Colloidal Quantum Dot Transistors. *ACS Applied Materials & Interfaces* **12**, 31591-31600 (2020).

129. Bao Y, Balandin AA, Liu JL, Liu J, Xie YH. Experimental investigation of Hall mobility in Ge/Si quantum dot superlattices. *Applied Physics Letters* **84**, 3355-3357 (2004).

130. Campi D, Mounet N, Gibertini M, Pizzi G, Marzari N. Expansion of the Materials Cloud 2D Database. *ACS Nano* **17**, 11268-11278 (2023).

131. Ha V-A, Giustino F. High-throughput screening of 2D materials identifies p-type monolayer WS2 as potential ultra-high mobility semiconductor. *npj Computational Materials* **10**, (2024).

132. Zhang Z, Xie J, Yang D, Wang Y, Si M, Xue D. Manifestation of unexpected semiconducting properties in few-layer orthorhombic arsenene. *Applied Physics Express* **8**, (2015).

133. Mortazavi B, Shojaei F, Azizi M, Rabczuk T, Zhuang X. As2S3, As2Se3 and As2Te3 nanosheets: superstretchable semiconductors with anisotropic carrier mobilities and optical properties. *Journal of Materials Chemistry C* **8**, 2400-2410 (2020).

134. Tang C, Zhang L, Zhang C, MacLeod J, Ostrikov K, Du A. Highly stable two-dimensional gold selenide with large in-plane anisotropy and ultrahigh carrier mobility. *Nanoscale Horizons* **5**, 366-371 (2020).

135. Khatami MM, Gaddemane G, Van de Put ML, Moravvej-Farshi MK, Vandenberghe WG. Electronic transport properties of hydrogenated and fluorinated graphene: a computational study. *Journal of Physics: Condensed Matter* **32**, (2020).

136. Mahdavifar Z, Shojaei F. CdInGaS4: An unexplored two- dimensional materials with desirable band gap for optoelectronic devices. *Journal of Alloys and Compounds* **854**, (2021).

137. Guo Y, Zhang Y, Yuan S, Wang B, Wang J. Chromium sulfide halide monolayers: intrinsic ferromagnetic semiconductors with large spin polarization and high carrier mobility. *Nanoscale* **10**, 18036-18042 (2018).

138. Li W, *et al.* High thermoelectric properties of Cu2Te Ag2Te composite with Fe addition and non-stoichiometric Te. *Journal of Materiomics* **10**, 37-44 (2024).

139. Zhang G, Lu K, Wang Y, Wang H, Chen Q. Mechanical and electronic properties of α−M2X3 (M=Ga, In; X=S, Se)monolayers. *Physical Review B* **105**, (2022).

140. Zhang C, Wang R, Mishra H, Liu Y. Two-Dimensional Semiconductors with High Intrinsic Carrier Mobility at Room Temperature. *Physical Review Letters* **130**, (2023).

141. Wang J, Zhang R, Xiao H, Zhou R, Gao T. The carrier mobility of monolayer and bulk GaS: from first-principles calculations. *Physical Chemistry Chemical Physics* **24**, 21666-21673 (2022).

142. Cheng L, Zhang C, Liu Y. Why Two-Dimensional Semiconductors Generally Have Low Electron Mobility. *Physical Review Letters* **125**, (2020).

143. Liu C-S, Yang X-L, Liu J, Ye X-J. Exfoliated Monolayer GeI2: Theoretical Prediction of a Wide-Band Gap Semiconductor with Tunable Half-Metallic Ferromagnetism. *The Journal of Physical Chemistry C* **122**, 22137-22142 (2018).

144. Zhao T, Sun Y, Shuai Z, Wang D. GeAs2: A IV–V Group Two-Dimensional Semiconductor with Ultralow Thermal Conductivity and High Thermoelectric Efficiency. *Chemistry of Materials* **29**, 6261-6268 (2017).

145. Guo Y, Gao N, Bai Y, Zhao J, Zeng XC. Monolayered semiconducting GeAsSe and SnSbTe with ultrahigh hole mobility. *Frontiers of Physics* **13**, (2018).

146. Fang W-y*, et al.* Theoretical investigation of the electronic structure and thermoelectric performance of 2D GeSb2Te4 and GeBi2Te4. *Vacuum* **216**, (2023).

147. Shi L-B, Cao S, Yang M. Strain behavior and Carrier mobility for novel two-dimensional semiconductor of GeP: First principles calculations. *Physica E: Low-dimensional Systems and Nanostructures* **107**, 124-130 (2019).

148. Zhang X, Zhang Z, Zhao X, Wu D, Zhang X, Zhou Z. Tetragonal-structured anisotropic 2D metal nitride monolayers and their halides with versatile promises in energy storage and conversion. *Journal of Materials Chemistry A* **5**, 2870-2875 (2017).

149. Zhang W, Huang Z, Zhang W, Li Y. Two-dimensional semiconductors with possible high room temperature mobility. *Nano Research* **7**, 1731-1737 (2014).

150. Banjade HR, Pan J, Yan Q. Monolayer 2D semiconducting tellurides for high-mobility electronics. *Physical Review Materials* **5**, (2021).

151. Arora A, De Sarkar A. Advancing intrinsic carrier mobility estimation in transition metal trichalcogenide monolayers using DFT-BTE. *Applied Physics Letters* **124**, (2024).

152. Zhao Q*, et al.* Flexibility and anisotropy of MX3 (M = Zr, Hf; X = S, Se): New semiconductors with high photovoltaic performance. *Journal of Applied Physics* **134**, (2023).

153. Nian T, Wang Z, Dong B. Thermoelectric properties of α-In2Se3 monolayer. *Applied Physics Letters* **118**, (2021).

154. Kang P, Michaud-Rioux V, Kong XH, Yu GH, Guo H. Calculated carrier mobility of h-BN/γ-InSe/h-BN van der Waals heterostructures. *2D Materials* **4**, (2017).

155. Chen Z-Y, Xiong M, Zeng Z-Y, Chen X-R, Chen Q-F. Comparative study of elastic, thermodynamic properties and carrier mobility of InX (X = O, S, Se, Te) monolayers via first-principles. *Solid State Communications* **326**, (2021).

156. Zeng S, *et al.* Excellent thermoelectric performance of layered trigonal crystals XPt2Se3 (X = K, Rb). *Applied Physics Letters* **124**, (2024).

157. Abyaz B, Mahdavifar Z, Schreckenbach G. 2D Mg2M2X5 (M = B, Al, Ga, In, Tl; X = S, Se, Te) monolayers: Novel stable semiconductors for water splitting photocatalysts. *Applied Surface Science* **621**, (2023).

158. Wang X, Li X, Wang X, Ju W. Highly stable two-dimensional α1-MA2Z4 (M = Mg, Ca, Sr; A = Al; Z = S, Se) monolayers with promising photocatalysis and piezoresistive effect. *Applied Physics Letters* **123**, (2023).

159. Li P, Zhang W, Liang C, Zeng XC. Two-dimensional MgX2Se4 (X = Al, Ga) monolayers with tunable electronic properties for optoelectronic and photocatalytic applications. *Nanoscale* **11**, 19806-19813 (2019).

160. Zhang X, Zhao X, Wu D, Jing Y, Zhou Z. MnPSe3 Monolayer: A Promising 2D Visible-Light Photohydrolytic Catalyst with High Carrier Mobility. *Advanced Science* **3**, (2016).

161. Zhang W-B, Qu Q, Lai K. High-Mobility Transport Anisotropy in Few-Layer MoO3 and Its Origin. *ACS Applied Materials & Interfaces* **9**, 1702-1709 (2017).

162. Li W, He Q, Li X-P, Ma D-S, Fu B. Two-dimensional double kagome lattice nitrogene: A direct band gap semiconductor with nontrivial corner state. *Physical Review B* **110**, (2024).

163. Pan L, Wan Y-L, Wang Z-Q, Geng H-Y, Chen X-R. Two-dimensional anisotropic monolayers NbOX2 (X = Cl, Br, I): Promising candidates for photocatalytic water splitting with high solar-to-hydrogen efficiency. *Journal of Applied Physics* **134**, (2023).

164. Su T, *et al.* 2D janus niobium oxydihalide NbOXY: Multifunctional piezoelectric semiconductor for electronics, photonics, sensing and sustainable energy applications. *Materials Today Physics* **31**, (2023).

165. Qiao M, Wang C, Jing Y, Zhou X, Li Y. NbS2Cl2 monolayer: A promising 2D semiconductor for photocatalytic water splitting. *FlatChem* **27**, (2021).

166. Wani AF, Rani B, Sharopov UB, Dhiman S, Kaur K. Thermoelectric investigation of transition metal oxide NiO2: A first principles study. *International Journal of Energy Research* **46**, 8527-8535 (2022).

167. Tan J, Hu H, Cai B, Xu D, Ouyang G. Instability of the magnetic state of MPX3(M=Mn,Ni;X=S,Se) monolayers induced by strain and doping. *Physical Review B* **106**, (2022).

168. Xie W, Pang J, Yang J, Kuang X, Mao A. Highly-efficient heterojunction solar cells based on 2D Janus transition-metal nitride halide (TNH) monolayers with ultrahigh carrier mobility. *Nanoscale* **15**, 18328-18336 (2023).

169. Wang Y, Li Y, Chen Z. Not your familiar two dimensional transition metal disulfide: structural and electronic properties of the PdS2monolayer. *Journal of Materials Chemistry C* **3**, 9603-9608 (2015).

170. Jakhar M, Kumar A. Tunable photocatalytic water splitting and solar-to-hydrogen efficiency in β-PdSe2monolayer. *Catalysis Science & Technology* **11**, 6445-6454 (2021).

171. Jing Y, Ma Y, Wang Y, Li Y, Heine T. Ultrathin Layers of PdPX (X=S, Se): Two Dimensional Semiconductors for Photocatalytic Water Splitting. *Chemistry – A European Journal* **23**, 13612-13616 (2017).

172. Yu S, Zhu H, Eshun K, Shi C, Zeng M, Li Q. Strain-engineering the anisotropic electrical conductance in ReS2 monolayer. *Applied Physics Letters* **108**, (2016).

173. Ying Y, Fan K, Zhu S, Luo X, Huang H. Theoretical Investigation of Monolayer RhTeCl Semiconductors as Photocatalysts for Water Splitting. *The Journal of Physical Chemistry C* **124**, 639-646 (2019).

174. Wang Y*, et al.* Many-body Effect, Carrier Mobility, and Device Performance of Hexagonal Arsenene and Antimonene. *Chemistry of Materials* **29**, 2191-2201 (2017).

175. Wang F, Yang C-L, Wang M-S, Ma X. Photocatalytic hydrogen evolution reaction with high solar-to-hydrogen efficiency driven by the Sb2S3 monolayer and RuI2/Sb2S3 heterostructure with solar light. *Journal of Power Sources* **532**, (2022).

176. Liang Y, Li J, Jin H, Huang B, Dai Y. Propose two-dimensional Sb2Te2X (X = S, Se) with isotropic electron mobility and remarkable visible-light response. *Physical Chemistry Chemical Physics* **21**, 14904-14910 (2019).

177. Wang C, Jing Y, Zhou X, Li Y-f. Sb2TeSe2 Monolayers: Promising 2D Semiconductors for Highly Efficient Excitonic Solar Cells. *ACS Omega* **6**, 20590-20597 (2021).

178. Sun X, Zheng K, Cai M, Bao J, Chen X. Excellent carrier mobility and opto/electronics performance material prediction: Focusing on single layer X2Te3 (X = Sb, Bi). *Applied Surface Science* **491**, 690-697 (2019).

179. Guo S-D, Guo X-S, Liu Z-Y, Quan Y-N. Large piezoelectric coefficients combined with high electron mobilities in Janus monolayer XTeI (X = Sb and Bi): A first-principles study. *Journal of Applied Physics* **127**, (2020).

180. Lai K, Yan C-L, Gao L-Q, Zhang W-B. AI3 (A = As, Sb) Single Layers and Their vdW Heterostructure for Photocatalysis and Solar Cell Applications. *The Journal of Physical Chemistry C* **122**, 7656-7663 (2018).

181. Zhou L, Zhang Y, Zhuo Z, Neukirch AJ, Tretiak S. Interlayer-Decoupled Sc-Based Mxene with High Carrier Mobility and Strong Light-Harvesting Ability. *The Journal of Physical Chemistry Letters* **9**, 6915-6920 (2018).

182. Chen Q, Ding Q, Wang Y, Xu Y, Wang J. Electronic and Magnetic Properties of a Two-Dimensional Transition Metal Phosphorous Chalcogenide TMPS4. *The Journal of Physical Chemistry C* **124**, 12075-12080 (2020).

183. Li P*, et al.* Semiconducting Scandium/Yttrium Chalcohalides: Promising Visible-Light-Driven Photocatalysts for Overall Water Splitting. *ACS Catalysis* **15**, 4533-4540 (2025).

184. Khatami M*, et al.* Electronic Transport Properties of Silicane Determined from First Principles. *Materials* **12**,  (2019).

185. Li P*, et al.* Two-Dimensional IV–V Monolayers with Highly Anisotropic Carrier Mobility and Electric Transport Properties. *The Journal of Physical Chemistry Letters* **12**, 1058-1065 (2021).

186. Bai S, Niu C-Y, Yu W, Zhu Z, Cai X, Jia Y. Strain Tunable Bandgap and High Carrier Mobility in SiAs and SiAs2 Monolayers from First-Principles Studies. *Nanoscale Research Letters* **13**,  (2018).

187. Shojaei F, Mortazavi B, Zhuang X, Azizi M. Silicon diphosphide (SiP2) and silicon diarsenide (SiAs2): Novel stable 2D semiconductors with high carrier mobilities, promising for water splitting photocatalysts. *Materials Today Energy* **16**,  (2020).

188. Yao Y, Wu B, Wang T, Lu K, Yin J. Strain engineering and stacking pattern tune the electrical conductivity of two-dimensional SiPS. *Semiconductor Science and Technology* **35**,  (2020).

189. Guo Y*, et al.* Eighteen functional monolayer metal oxides: wide bandgap semiconductors with superior oxidation resistance and ultrahigh carrier mobility. *Nanoscale Horizons* **4**, 592-600 (2019).

190. Xinyu Liu Sl, Kacek K. Furdyna, Tengfei Luo, Yong-Hang Zhang (Eds.). *Chalcogenide: From 3D to 2D and Beyond*. Woodhead Publishing (2020).

191. Li Y*, et al.* Evolutional carrier mobility and power factor of two-dimensional tin telluride due to quantum size effects. *Journal of Materials Chemistry C* **8**, 4181-4191 (2020).

192. Yao C, Rao X, Fang W, Sheng X, Peng S, Zhang P. Single-layer XBi2Se4 (X = Sn Pb) with multi-valley band structures and excellent thermoelectric performance. *Ceramics International* **49**, 25455-25462 (2023).

193. Xie Q-Y, Liu P-F, Ma J-J, Kuang F-G, Zhang K-W, Wang B-T. Monolayer SnI2: An Excellent p-Type Thermoelectric Material with Ultralow Lattice Thermal Conductivity. *Materials* **15**,  (2022).

194. Lin M*, et al.* Two-dimensional nanoporous metal chalcogenophosphates MP2X6 with high electron mobilities. *Applied Surface Science* **493**, 1334-1339 (2019).

195. Zhou W*, et al.* Unusual Electronic Transitions in Two-dimensional Layered SnSb2Te4 Driven by Electronic State Rehybridization. *Physical Review Applied* **11**,  (2019).

196. Shi X*, et al.* Janus 2D titanium nitride halide TiNX0.5Y0.5 (X, Y = F, Cl, or Br, and X ≠ Y) monolayers with giant out-of-plane piezoelectricity and high carrier mobility. *Physical Chemistry Chemical Physics* **23**, 3637-3645 (2021).

197. Dai J, Zeng XC. Titanium Trisulfide Monolayer: Theoretical Prediction of a New Direct-Gap Semiconductor with High and Anisotropic Carrier Mobility. *Angewandte Chemie International Edition* **54**, 7572-7576 (2015).

198. Amin B, Kaloni TP, Schwingenschlögl U. Strain engineering of WS2, WSe2, and WTe2. *RSC Advances* **4**,  (2014).

199. Ould-Mohamed M, Ouahrani T, Ougherb C. Biaxial strain enhanced electronic and photocatalytic properties of Y2CBr2 MXene. *Journal of Physics and Chemistry of Solids* **199**, (2025).

200. Nitta N, Wu F, Lee JT, Yushin G. Li-ion battery materials: present and future. *Materials Today* **18**, 252-264 (2015).

201. Arroyo-de Dompablo ME, Ponrouch A, Johansson P, Palacín MR. Achievements, Challenges, and Prospects of Calcium Batteries. *Chemical Reviews* **120**, 6331-6357 (2019).

202. Yuan B*, et al.* Revitalizing Chlorine–Based Batteries for Low–Cost and High–Performance Energy Storage. *Advanced Energy Materials* **14**,  (2023).

203. Nzereogu PU, Omah AD, Ezema FI, Iwuoha EI, Nwanya AC. Anode materials for lithium-ion batteries: A review. *Applied Surface Science Advances* **9**,  (2022).

204. Goriparti S, Miele E, De Angelis F, Di Fabrizio E, Proietti Zaccaria R, Capiglia C. Review on recent progress of nanostructured anode materials for Li-ion batteries. *Journal of Power Sources* **257**, 421-443 (2014).

205. Wu F, Yushin G. Conversion cathodes for rechargeable lithium and lithium-ion batteries. *Energy & Environmental Science* **10**, 435-459 (2017).

206. Muthu P*, et al.* Review of Transition Metal Chalcogenides and Halides as Electrode Materials for Thermal Batteries and Secondary Energy Storage Systems. *ACS Omega*, (2024).

207. Liang Y, Tao Z, Chen J. Organic Electrode Materials for Rechargeable Lithium Batteries. *Advanced Energy Materials* **2**, 742-769 (2012).

208. Stevens DA, Dahn JR. High Capacity Anode Materials for Rechargeable Sodium-Ion Batteries. *Journal of The Electrochemical Society* **147**,  (2000).

209. Komaba S, Matsuura Y, Ishikawa T, Yabuuchi N, Murata W, Kuze S. Redox reaction of Sn-polyacrylate electrodes in aprotic Na cell. *Electrochemistry Communications* **21**, 65-68 (2012).

210. Li Y, Lai X, Qu J, Lai Q, Yi T. Research Progress in Regulation Strategies of High-Performance Antimony-Based Anode Materials for Sodium Ion Batteries. *Acta Physico Chimica Sinica* **0**, 2204049-2204040 (2022).

211. Liu Y*, et al.* Red-phosphorus-impregnated carbon nanofibers for sodium-ion batteries and liquefaction of red phosphorus. *Nature Communications* **11**, (2020).

212. Dong J, Jiang Y, Wang R, Wei Q, An Q, Zhang X. Review and prospects on the low-voltage Na2Ti3O7 anode materials for sodium-ion batteries. *Journal of Energy Chemistry* **88**, 446-460 (2024).

213. Liang S*, et al.* Bronze-Phase TiO2 as Anode Materials in Lithium and Sodium-Ion Batteries. *Advanced Functional Materials* **32**, (2022).

214. Zhang L, Wang W, Lu S, Xiang Y. Carbon Anode Materials: A Detailed Comparison between Na-ion and K-ion Batteries. *Advanced Energy Materials* **11**, (2021).

215. Ling C, Mizuno F. Boron-doped graphene as a promising anode for Na-ion batteries. *Phys Chem Chem Phys* **16**, 10419-10424 (2014).

216. Jiang Y, Wang Y, Ni J, Li L. Molybdenum-based materials for sodium-ion batteries. *InfoMat* **3**, 339-352 (2021).

217. Yu DYW*, et al.* High-capacity antimony sulphide nanoparticle-decorated graphene composite as anode for sodium-ion batteries. *Nature Communications* **4**, (2013).

218. Zhang H, Hasa I, Passerini S. Beyond Insertion for Na-Ion Batteries: Nanostructured Alloying and Conversion Anode Materials. *Advanced Energy Materials* **8**, (2018).

219. Kim DH*, et al.* RT-XAMF and TR-XRD studies of solid-state synthesis and thermal stability of NaNiO2 as cathode material for sodium-ion batteries. *Ceramics International* **48**, 19675-19680 (2022).

220. Zhang X*, et al.* Na3V2(PO4)3: an advanced cathode for sodium-ion batteries. *Nanoscale* **11**, 2556-2576 (2019).

221. Gezović A*, et al.* An effective approach to reaching the theoretical capacity of a low-cost and environmentally friendly Na4Fe3(PO4)2(P2O7) cathode for Na-ion batteries. *Electrochimica Acta* **476**, (2024).

222. Zhang H*, et al.* Organic Cathode Materials for Sodium-Ion Batteries: From Fundamental Research to Potential Commercial Application. *Advanced Functional Materials* **32**, (2021).

223. Xiao JC, Si J, Pan B, Chen C. Revealing the Kinetic Limitation of Na7Fe7(PO4)6F3 Polyanion Cathode with High Theoretical Capacity for Sodium-Ion Batteries. *Small* **21**, (2025).

224. Liu J, Wang Y, Jiang N, Wen B, Yang C, Liu Y. Vacancies-regulated Prussian Blue Analogues through Precipitation Conversion for Cathodes in Sodium-ion Batteries with Energy Densities over 500 Wh/kg. *Angewandte Chemie* **136**, (2024).

225. Li J, *et al.* Sulphur-doped reduced graphene oxide sponges as high-performance free-standing anodes for K-ion storage. *Nano Energy* **53**, 415-424 (2018).

226. McCulloch WD, Ren X, Yu M, Huang Z, Wu Y. Potassium-Ion Oxygen Battery Based on a High Capacity Antimony Anode. *ACS Applied Materials & Interfaces* **7**, 26158-26166 (2015).

227. Deng Q, Zhao Y, Zhu X, Yang K, Li M. Recent Advances and Challenges in Ti-Based Oxide Anodes for Superior Potassium Storage. *Nanomaterials* **13**, (2023).

228. Sannyal A, Zhang Z, Gao X, Jang J. Two-dimensional sheet of germanium selenide as an anode material for sodium and potassium ion batteries: First-principles simulation study. *Computational Materials Science* **154**, 204-211 (2018).

229. Xu Y, *et al.* Recent advances in rational design for high-performance potassium-ion batteries. *Chemical Society Reviews* **53**, 7202-7298 (2024).

230. Zhang C, Zhao H, Lei Y. Recent Research Progress of Anode Materials for Potassium-ion Batteries. *Energy & Environmental Materials* **3**, 105-120 (2020).

231. Liu S, *et al.* Recent Advances and Perspectives of Battery-Type Anode Materials for Potassium Ion Storage. *ACS Nano* **15**, 18931-18973 (2021).

232. Song K, Liu C, Mi L, Chou S, Chen W, Shen C. Recent Progress on the Alloy-Based Anode for Sodium-Ion Batteries and Potassium-Ion Batteries. *Small* **17**, (2019).

233. Rajagopalan R, Tang Y, Ji X, Jia C, Wang H. Advancements and Challenges in Potassium Ion Batteries: A Comprehensive Review. *Advanced Functional Materials* **30**, (2020).

234. Zhang Q, Wang Z, Zhang S, Zhou T, Mao J, Guo Z. Cathode Materials for Potassium-Ion Batteries: Current Status and Perspective. *Electrochemical Energy Reviews* **1**, 625-658 (2018).

235. Jian Z, Liang Y, Rodríguez-Pérez IA, Yao Y, Ji X. Poly(anthraquinonyl sulfide) cathode for potassium-ion batteries. *Electrochemistry Communications* **71**, 5-8 (2016).

236. Hosaka T, Shimamura T, Kubota K, Komaba S. Polyanionic Compounds for Potassium-Ion Batteries. *The Chemical Record* **19**, 735-745 (2018).

237. Xue Q, *et al.* Vitamin K as a high-performance organic anode material for rechargeable potassium ion batteries. *Journal of Materials Chemistry A* **6**, 12559-12564 (2018).

238. Liu S, Kang L, Jun SC. Challenges and Strategies toward Cathode Materials for Rechargeable Potassium-Ion Batteries. *Advanced Materials* **33**, (2021).

239. Xu YS, *et al.* High-Performance Cathode Materials for Potassium-Ion Batteries: Structural Design and Electrochemical Properties. *Advanced Materials* **33**, (2021).

240. Chen H, *et al.* Improved charge extraction in inverted perovskite solar cells with dual-site-binding ligands. *Science* **384**, 189-193 (2024).

241. Zhou H, *et al.* Efficient and stable perovskite mini-module via high-quality homogeneous perovskite crystallization and improved interconnect. *Nature Communications* **15**, 6679 (2024).

242. Li G, *et al.* Highly efficient p-i-n perovskite solar cells that endure temperature variations. *Science* **379**, 399-403 (2023).

243. Zheng X, *et al.* Managing grains and interfaces via ligand anchoring enables 22.3%-efficiency inverted perovskite solar cells. *Nature Energy* **5**, 131-140 (2020).

244. Yu D, *et al.* Quasi-2D Bilayer Surface Passivation for High Efficiency Narrow Bandgap Perovskite Solar Cells. *Angewandte Chemie International Edition* **61**, e202202346 (2022).

245. Ru P, *et al.* High Electron Affinity Enables Fast Hole Extraction for Efficient Flexible Inverted Perovskite Solar Cells. *Advanced Energy Materials* **10**, 1903487 (2020).

246. Lee J, Kim G-W, Kim M, Park SA, Park T. Nonaromatic Green-Solvent-Processable, Dopant-Free, and Lead-Capturable Hole Transport Polymers in Perovskite Solar Cells with High Efficiency. *Advanced Energy Materials* **10**, 1902662 (2020).

247. Zhang J, *et al.* Dibenzo[b,d]thiophene-Cored Hole-Transport Material with Passivation Effect Enabling the High-Efficiency Planar p–i–n Perovskite Solar Cells with 83% Fill Factor. *Solar RRL* **4**, 1900421 (2020).

248. Wang Y, *et al.* Dopant-Free Small-Molecule Hole-Transporting Material for Inverted Perovskite Solar Cells with Efficiency Exceeding 21%. *Advanced Materials* **31**, 1902781 (2019).

249. Jiang K, *et al.* Dopant-Free Organic Hole-Transporting Material for Efficient and Stable Inverted All-Inorganic and Hybrid Perovskite Solar Cells. *Advanced Materials* **32**, 1908011 (2020).

250. Li X, Zhang W, Zhang W, Wang H-Q, Fang J. Spontaneous grain polymerization for efficient and stable perovskite solar cells. *Nano Energy* **58**, 825-833 (2019).

251. Xie F, *et al.* Vertical recrystallization for highly efficient and stable formamidinium-based inverted-structure perovskite solar cells. *Energy & Environmental Science* **10**, 1942-1949 (2017).

252. Yang B, Ouyang D, Huang Z, Ren X, Zhang H, Choy WCH. Multifunctional Synthesis Approach of In:CuCrO2 Nanoparticles for Hole Transport Layer in High-Performance Perovskite Solar Cells. *Advanced Functional Materials* **29**, 1902600 (2019).

253. Sun Q, *et al.* High-efficiency planar p-i-n perovskite solar cells based on dopant-free dibenzo[b,d]furan-centred linear hole transporting material. *Journal of Power Sources* **449**, 227488 (2020).

254. Tsai H, *et al.* Light-induced lattice expansion leads to high-efficiency perovskite solar cells. *Science* **360**, 67-70 (2018).

255. Hu J, *et al.* Polyfluorene Copolymers as High-Performance Hole-Transport Materials for Inverted Perovskite Solar Cells. *Solar RRL* **4**, 1900384 (2020).

256. Al-Ashouri A, *et al.* Conformal monolayer contacts with lossless interfaces for perovskite single junction and monolithic tandem solar cells. *Energy & Environmental Science* **12**, 3356-3369 (2019).

257. Wu F, *et al.* Sulfur-annulated perylenediimide as an interfacial material enabling inverted perovskite solar cells with over 20% efficiency and high fill factors exceeding 83%. *Journal of Materials Chemistry A* **7**, 21176-21181 (2019).

258. Reddy SS, *et al.* Highly efficient air-stable/hysteresis-free flexible inverted-type planar perovskite and organic solar cells employing a small molecular organic hole transporting material. *Nano Energy* **41**, 10-17 (2017).

259. Liang J, *et al.* Revealing the Mechanism of π Aromatic Molecule as an Effective Passivator and Stabilizer in Highly Efficient Wide-Bandgap Perovskite Solar Cells. *Solar RRL* **5**, 2100249 (2021).

260. Li Y, *et al.* High-Performance Perovskite Solar Cells with a Non-doped Small Molecule Hole Transporting Layer. *ACS Applied Energy Materials* **2**, 1634-1641 (2019).

261. Xiao Q, Wu F, Han M, Li Z, Zhu L, Li Za. A pseudo-two-dimensional conjugated polysquaraine: an efficient p-type polymer semiconductor for organic photovoltaics and perovskite solar cells. *Journal of Materials Chemistry A* **6**, 13644-13651 (2018).

262. Zhang L, Zhou X, Zhong X, Cheng C, Tian Y, Xu B. Hole-transporting layer based on a conjugated polyelectrolyte with organic cations enables efficient inverted perovskite solar cells. *Nano Energy* **57**, 248-255 (2019).

263. Cao Y, *et al.* Dopant-free molecular hole transport material that mediates a 20% power conversion efficiency in a perovskite solar cell. *Energy & Environmental Science* **12**, 3502-3507 (2019).

264. Pérez-del-Rey D, *et al.* Molecular Passivation of MoO3: Band Alignment and Protection of Charge Transport Layers in Vacuum-Deposited Perovskite Solar Cells. *Chemistry of Materials* **31**, 6945-6949 (2019).

265. Chen Y, *et al.* Rational Design of Dopant-Free Coplanar D-π-D Hole-Transporting Materials for High-Performance Perovskite Solar Cells with Fill Factor Exceeding 80%. *Advanced Energy Materials* **9**, 1901268 (2019).

266. Huang Z, Ouyang D, Ma R, Wu W, Roy VAL, Choy WCH. A General Method: Designing a Hypocrystalline Hydroxide Intermediate to Achieve Ultrasmall and Well-Dispersed Ternary Metal Oxide for Efficient Photovoltaic Devices. *Advanced Functional Materials* **29**, 1904684 (2019).

267. Zhang H, *et al.* Low-Temperature Solution-Processed CuCrO2 Hole-Transporting Layer for Efficient and Photostable Perovskite Solar Cells. *Advanced Energy Materials* **8**, 1702762 (2018).

268. Rao H, *et al.* A 19.0% efficiency achieved in CuOx-based inverted CH3NH3PbI3−xClx solar cells by an effective Cl doping method. *Nano Energy* **27**, 51-57 (2016).

269. Huang C, *et al.* Dopant-Free Hole-Transporting Material with a C3h Symmetrical Truxene Core for Highly Efficient Perovskite Solar Cells. *Journal of the American Chemical Society* **138**, 2528-2531 (2016).

270. Wang YK, *et al.* Fluorinating Dopant-Free Small-Molecule Hole-Transport Material to Enhance the Photovoltaic Property. *ACS Applied Materials & Interfaces* **13**, 7705-7713 (2021).

271. Yoo JJ, *et al.* Efficient perovskite solar cells via improved carrier management. *Nature* **590**, 587-593 (2021).

272. Wang P, *et al.* Gradient Energy Alignment Engineering for Planar Perovskite Solar Cells with Efficiency Over 23%. *Advanced Materials* **32**, 1905766 (2020).

273. Akin S. Hysteresis-Free Planar Perovskite Solar Cells with a Breakthrough Efficiency of 22% and Superior Operational Stability over 2000 h. *ACS Applied Materials & Interfaces* **11**, 39998-40005 (2019).

274. Sathiyan G, *et al.* Dual effective dopant based hole transport layer for stable and efficient perovskite solar cells. *Nano Energy* **72**, 104673 (2020).

275. Jung EH, *et al.* Efficient, stable and scalable perovskite solar cells using poly(3-hexylthiophene). *Nature* **567**, 511-515 (2019).

276. Jeon NJ, *et al.* A fluorene-terminated hole-transporting material for highly efficient and stable perovskite solar cells. *Nature Energy* **3**, 682-689 (2018).

277. Yang WS, *et al.* Iodide management in formamidinium-lead-halide–based perovskite layers for efficient solar cells. *Science* **356**, 1376-1379 (2017).

278. Chiang Y-H, Chou H-H, Cheng W-T, Li Y-R, Yeh C-Y, Chen P. Porphyrin Dimers as Hole-Transporting Layers for High-Efficiency and Stable Perovskite Solar Cells. *ACS Energy Letters* **3**, 1620-1626 (2018).

279. Ren M, Wang J, Xie X, Zhang J, Wang P. Double-Helicene-Based Hole-Transporter for Perovskite Solar Cells with 22% Efficiency and Operation Durability. *ACS Energy Letters* **4**, 2683-2688 (2019).

280. Ma X-J, *et al.* Planar starburst hole-transporting materials for highly efficient perovskite solar cells. *Nano Energy* **63**, 103865 (2019).

281. Tsarev S, *et al.* A new polytriarylamine derivative for dopant-free high-efficiency perovskite solar cells. *Sustainable Energy & Fuels* **3**, 2627-2632 (2019).

282. Hou Y, *et al.* A generic interface to reduce the efficiency-stability-cost gap of perovskite solar cells. *Science* **358**, 1192-1197 (2017).

283. Elseman AM*, et al.* Superior Stability and Efficiency Over 20% Perovskite Solar Cells Achieved by a Novel Molecularly Engineered Rutin–AgNPs/Thiophene Copolymer. *Advanced Science* **5**, 1800568 (2018).

284. Xu N*, et al.* D–π–D molecular semiconductors for perovskite solar cells: the superior role of helical versus planar π-linkers. *Chemical Science* **11**, 3418-3426 (2020).

285. Xu N*, et al.* An Oxa[5]helicene-Based Racemic Semiconducting Glassy Film for Photothermally Stable Perovskite Solar Cells. *iScience* **15**, 234-242 (2019).

286. Azmi R*, et al.* High-performance dopant-free conjugated small molecule-based hole-transport materials for perovskite solar cells. *Nano Energy* **44**, 191-198 (2018).

287. Lee DY, Sivakumar G, Manju, Misra R, Seok SI. Carbazole-Based Spiro[fluorene-9,9′-xanthene] as an Efficient Hole-Transporting Material for Perovskite Solar Cells. *ACS Applied Materials & Interfaces* **12**, 28246-28252 (2020).

288. Hou Y*, et al.* Assembling Mesoscale-Structured Organic Interfaces in Perovskite Photovoltaics. *Advanced Materials* **31**, 1806516 (2019).

289. Bi D, Xu B, Gao P, Sun L, Grätzel M, Hagfeldt A. Facile synthesized organic hole transporting material for perovskite solar cell with efficiency of 19.8%. *Nano Energy* **23**, 138-144 (2016).

290. Wei Q, Ning Z. Planar core based starburst triphenylamine molecules as hole transporting materials for high-performance perovskite solar cells. *Science China Chemistry* **62**, 5-6 (2019).

291. Cai F*, et al.* Ionic Additive Engineering Toward High-Efficiency Perovskite Solar Cells with Reduced Grain Boundaries and Trap Density. *Advanced Functional Materials* **28**, 1801985 (2018).

292. Cao J*, et al.* Plant Sunscreen and Co(II)/(III) Porphyrins for UV-Resistant and Thermally Stable Perovskite Solar Cells: From Natural to Artificial. *Advanced Materials* **30**, 1800568 (2018).

293. Yin X*, et al.* Dithieno[3,2-b:2′,3′-d]pyrrol-Cored Hole Transport Material Enabling Over 21% Efficiency Dopant-Free Perovskite Solar Cells. *Advanced Functional Materials* **29**, 1904300 (2019).

294. Arora N*, et al.* Perovskite solar cells with CuSCN hole extraction layers yield stabilized efficiencies greater than 20%. *Science* **358**, 768-771 (2017).

295. Zhang F*, et al.* Polymeric, Cost-Effective, Dopant-Free Hole Transport Materials for Efficient and Stable Perovskite Solar Cells. *Journal of the American Chemical Society* **141**, 19700-19707 (2019).

296. Zhang L, Liu C, Wang X, Tian Y, Jen AKY, Xu B. Side-Chain Engineering on Dopant-Free Hole-Transporting Polymers toward Highly Efficient Perovskite Solar Cells (20.19%). *Advanced Functional Materials* **29**, 1904856 (2019).

297. Ding X*, et al.* Highly efficient phenothiazine 5,5-dioxide-based hole transport materials for planar perovskite solar cells with a PCE exceeding 20%. *Journal of Materials Chemistry A* **7**, 9510-9516 (2019).

298. Kong X*, et al.* Dopant-free F-substituted benzodithiophene copolymer hole-transporting materials for efficient and stable perovskite solar cells. *Journal of Materials Chemistry A* **8**, 1858-1864 (2020).

299. Shao J-Y, Yang N, Guo W, Cui B-B, Chen Q, Zhong Y-W. Introducing fluorene into organic hole transport materials to improve mobility and photovoltage for perovskite solar cells. *Chemical Communications* **55**, 13406-13409 (2019).

300. Feng Y*, et al.* High-Performance and Stable Perovskite Solar Cells Based on Dopant-Free Arylamine-Substituted Copper(II) Phthalocyanine Hole-Transporting Materials. *Advanced Energy Materials* **9**, 1901019 (2019).

301. Li Y*, et al.* SnS Quantum Dots as Hole Transporter of Perovskite Solar Cells. *ACS Applied Energy Materials* **2**, 3822-3829 (2019).

302. Li C*, et al.* Monoammonium Porphyrin for Blade-Coating Stable Large-Area Perovskite Solar Cells with >18% Efficiency. *Journal of the American Chemical Society* **141**, 6345-6351 (2019).

303. Kim G-W, Lee J, Kang G, Kim T, Park T. Donor–Acceptor Type Dopant-Free, Polymeric Hole Transport Material for Planar Perovskite Solar Cells (19.8%). *Advanced Energy Materials* **8**, 1701935 (2018).

304. Dong Z*, et al.* A dithieno[3,2-b:2′,3′-d]pyrrole-cored four-arm hole transporting material for over 19% efficiency dopant-free perovskite solar cells. *Journal of Materials Chemistry C* **7**, 9455-9459 (2019).

305. Cheng F*, et al.* 85 °C/85%-Stable n-i-p Perovskite Photovoltaics with NiO Hole Transport Layers Promoted By Perovskite Quantum Dots. *Advanced Science* **9**, 2201573 (2022).

306. Xu W*, et al.* Efficient Perovskite Solar Cells Fabricated by Co Partially Substituted Hybrid Perovskite. *Advanced Energy Materials* **8**, 1703178 (2018).

307. Yao K*, et al.* Fullerene-Anchored Core-Shell ZnO Nanoparticles for Efficient and Stable Dual-Sensitized Perovskite Solar Cells. *Joule* **3**, 417-431 (2019).

308. Jung S-K*, et al.* Homochiral Asymmetric-Shaped Electron-Transporting Materials for Efficient Non-Fullerene Perovskite Solar Cells. *ChemSusChem* **12**, 224-230 (2019).

309. Jung S-K*, et al.* Chiral Stereoisomer Engineering of Electron Transporting Materials for Efficient and Stable Perovskite Solar Cells. *Advanced Functional Materials* **30**, 1905951 (2020).

310. Heo JH, Lee S-C, Jung S-K, Kwon OP, Im SH. Efficient and thermally stable inverted perovskite solar cells by introduction of non-fullerene electron transporting materials. *Journal of Materials Chemistry A* **5**, 20615-20622 (2017).

311. Xing Y, Sun C, Yip HL, Bazan GC, Huang F, Cao Y. New fullerene design enables efficient passivation of surface traps in high performance p-i-n heterojunction perovskite solar cells. *Nano Energy* **26**, 7-15 (2016).

312. Hu T, Xiao S, Yang H, Chen L, Chen Y. Cerium oxide as an efficient electron extraction layer for p–i–n structured perovskite solar cells. *Chemical Communications* **54**, 471-474 (2018).

313. Liu X*, et al.* Energy level-modulated non-fullerene small molecule acceptors for improved VOC and efficiency of inverted perovskite solar cells. *Journal of Materials Chemistry A* **7**, 3336-3343 (2019).

314. Wu F, Gao W, Yu H, Zhu L, Li L, Yang C. Efficient small-molecule non-fullerene electron transporting materials for high-performance inverted perovskite solar cells. *Journal of Materials Chemistry A* **6**, 4443-4448 (2018).

315. Liu X, Jiang J, Wang F, Xiao Y, Sharp ID, Li Y. High Photovoltage Inverted Planar Heterojunction Perovskite Solar Cells with All-Inorganic Selective Contact Layers. *ACS Applied Materials & Interfaces* **11**, 46894-46901 (2019).

316. Shaikh DB*, et al.* Influences of Structural Modification of Naphthalenediimides with Benzothiazole on Organic Field-Effect Transistor and Non-Fullerene Perovskite Solar Cell Characteristics. *ACS Applied Materials & Interfaces* **11**, 44487-44500 (2019).

317. Liu W*, et al.* Molecular Aggregation of Naphthalene Diimide(NDI) Derivatives in Electron Transport Layers of Inverted Perovskite Solar Cells and Their Influence on the Device Performance. *Chem Asian J* **15**, 112-121 (2020).

318. Chang C-Y, Tsai B-C, Lin M-Z, Huang Y-C, Tsao C-S. An integrated approach towards the fabrication of highly efficient and long-term stable perovskite nanowire solar cells. *Journal of Materials Chemistry A* **5**, 22824-22833 (2017).

319. Tseng C-C*, et al.* Cu2O-HTM/SiO2-ETM assisted for synthesis engineering improving efficiency and stability with heterojunction planar perovskite thin-film solar cells. *Solar Energy* **204**, 270-279 (2020).

320. Gu P-Y*, et al.* Pushing up the efficiency of planar perovskite solar cells to 18.2% with organic small molecules as the electron transport layer. *Journal of Materials Chemistry A* **5**, 7339-7344 (2017).

321. Kim M*, et al.* Conformal quantum dot–SnO<sub>2</sub> layers as electron transporters for efficient perovskite solar cells. *Science* **375**, 302-306 (2022).

322. Jeong M*, et al.* Stable perovskite solar cells with efficiency exceeding 24.8% and 0.3-V voltage loss. *Science* **369**, 1615-1620 (2020).

323. Shini F, Thambidurai M, Harikesh PC, Mathews N, Huang Y, Dang C. Heterogeneous electron transporting layer for reproducible, efficient and stable planar perovskite solar cells. *Journal of Power Sources* **437**, 226907 (2019).

324. Tseng Z-L, Chiang C-H, Chang S-H, Wu C-G. Surface engineering of ZnO electron transporting layer via Al doping for high efficiency planar perovskite solar cells. *Nano Energy* **28**, 311-318 (2016).

325. Zhang P*, et al.* Enhanced thermal stability of electron transport layer-free perovskite solar cells via interface strain releasing. *Journal of Power Sources* **439**, 227091 (2019).

326. Zhao P*, et al.* Insulated Interlayer for Efficient and Photostable Electron-Transport-Layer-Free Perovskite Solar Cells. *ACS Applied Materials & Interfaces* **10**, 10132-10140 (2018).

327. Ahn N*, et al.* Trapped charge-driven degradation of perovskite solar cells. *Nature Communications* **7**, 13422 (2016).

328. Zhu Z*, et al.* A Low-Temperature, Solution-Processable Organic Electron-Transporting Layer Based on Planar Coronene for High-performance Conventional Perovskite Solar Cells. *Advanced Materials* **28**, 10786-10793 (2016).

329. Peng H*, et al.* High-performance cadmium sulphide-based planar perovskite solar cell and the cadmium sulphide/perovskite interfaces. *Journal of Photonics for Energy* **6**, 022002 (2016).

330. Wang X*, et al.* Cerium oxide standing out as an electron transport layer for efficient and stable perovskite solar cells processed at low temperature. *Journal of Materials Chemistry A* **5**, 1706-1712 (2017).

331. Wang Y-C, Li X, Zhu L, Liu X, Zhang W, Fang J. Efficient and Hysteresis-Free Perovskite Solar Cells Based on a Solution Processable Polar Fullerene Electron Transport Layer. *Advanced Energy Materials* **7**, 1701144 (2017).

332. Dong J*, et al.* Annealing-Free Cr2O3 Electron-Selective Layer for Efficient Hybrid Perovskite Solar Cells. *ChemSusChem* **11**, 619-628 (2018).

333. Yang D*, et al.* High efficiency planar-type perovskite solar cells with negligible hysteresis using EDTA-complexed SnO2. *Nature Communications* **9**, 3239 (2018).

334. Zhu W*, et al.* A non-equilibrium Ti4+ doping strategy for an efficient hematite electron transport layer in perovskite solar cells. *Dalton Transactions* **47**, 6404-6411 (2018).

335. Zhang M*, et al.* A low temperature processed fused-ring electron transport material for efficient planar perovskite solar cells. *Journal of Materials Chemistry A* **5**, 24820-24825 (2017).

336. Wang L, Liu F, Cai X, Ma T, Jiang C. Indium Zinc Oxide Electron Transport Layer for High-Performance Planar Perovskite Solar Cells. *The Journal of Physical Chemistry C* **122**, 28491-28496 (2018).

337. Wang Z, Lou J, Zheng X, Zhang W-H, Qin Y. Solution Processed Nb2O5 Electrodes for High Efficient Ultraviolet Light Stable Planar Perovskite Solar Cells. *ACS Sustainable Chemistry & Engineering* **7**, 7421-7429 (2019).

338. Zhang C*, et al.* Room-temperature solution-processed amorphous NbOx as an electron transport layer in high-efficiency photovoltaics. *Journal of Materials Chemistry A* **6**, 17882-17888 (2018).

339. Ryu U*, et al.* Nanocrystalline Titanium Metal–Organic Frameworks for Highly Efficient and Flexible Perovskite Solar Cells. *ACS Nano* **12**, 4968-4975 (2018).

340. McMeekin DP*, et al.* Crystallization Kinetics and Morphology Control of Formamidinium–Cesium Mixed-Cation Lead Mixed-Halide Perovskite via Tunability of the Colloidal Precursor Solution. *Advanced Materials* **29**, 1607039 (2017).

341. Zhao X*, et al.* 20% Efficient Perovskite Solar Cells with 2D Electron Transporting Layer. *Advanced Functional Materials* **29**, 1805168 (2019).

342. Ye Q-Q*, et al.* A SrGeO3 inorganic electron-transporting layer for high-performance perovskite solar cells. *Journal of Materials Chemistry A* **7**, 14559-14564 (2019).

343. Guo H*, et al.* Low-temperature processed yttrium-doped SrSnO3 perovskite electron transport layer for planar heterojunction perovskite solar cells with high efficiency. *Nano Energy* **59**, 1-9 (2019).

344. Huang P*, et al.* Room-Temperature and Aqueous Solution-Processed Two-Dimensional TiS2 as an Electron Transport Layer for Highly Efficient and Stable Planar n–i–p Perovskite Solar Cells. *ACS Applied Materials & Interfaces* **10**, 14796-14802 (2018).

345. Huang C*, et al.* Facile fabrication of highly efficient ETL-free perovskite solar cells with 20% efficiency by defect passivation and interface engineering. *Chemical Communications* **55**, 2777-2780 (2019).

346. Chen C*, et al.* Low-Temperature-Processed WO as Electron Transfer Layer for Planar Perovskite Solar Cells Exceeding 20% Efficiency. *Solar RRL* **4**, 1900499 (2020).

347. Dong X, Chen D, Zhou J, Zheng Y-Z, Tao X. High crystallization of a multiple cation perovskite absorber for low-temperature stable ZnO solar cells with high-efficiency of over 20%. *Nanoscale* **10**, 7218-7227 (2018).

348. Min H*, et al.* Efficient, stable solar cells by using inherent bandgap of α-phase formamidinium lead iodide. *Science* **366**, 749-753 (2019).

349. Li Z*, et al.* Ammonia for post-healing of formamidinium-based Perovskite films. *Nature Communications* **13**, 4417 (2022).

350. Subbiah AS*, et al.* High-Performance Perovskite Single-Junction and Textured Perovskite/Silicon Tandem Solar Cells via Slot-Die-Coating. *ACS Energy Letters* **5**, 3034-3040 (2020).

351. Liu C*, et al.* Promising applications of wide bandgap inorganic perovskites in underwater photovoltaic cells. *Solar Energy* **233**, 489-493 (2022).

352. Wang Y*, et al.* The Role of Dimethylammonium Iodide in $CsPbI_3$ Perovskite Fabrication: Additive or Dopant? *Angewandte Chemie International Edition* **58**, 16691-16696 (2019).

353. Yadavalli SK*, et al.* Mechanisms of exceptional grain growth and stability in formamidinium lead triiodide thin films for perovskite solar cells. *Acta Materialia* **193**, 10-18 (2020).

354. Xiang W*, et al.* Ba-induced phase segregation and band gap reduction in mixed-halide inorganic perovskite solar cells. *Nature Communications* **10**, (2019).

355. Wang J, Peng J, Sun Y, Liu X, Chen Y, Liang Z. $FAPbCl_3$ Perovskite as Alternative Interfacial Layer for Highly Efficient and Stable Polymer Solar Cells. *Advanced Electronic Materials* **2**, 1600329 (2016).

356. Ramavenkateswari K, Venkatachalam P. Stable Tin Chloride Perovskite Sensitized Silver Doped Titania Nanosticks Photoanode Solar Cells with Different Hole Transport Materials. *Journal of Inorganic and Organometallic Polymers and Materials* **26**, 981-990 (2016).

357. Dai WB, Xu S, Zhou J, Hu J, Huang K, Xu M. Lead-free, stable, and effective double FA4GeIISbIIICl12 perovskite for photovoltaic applications. *Solar Energy Materials and Solar Cells* **192**, 140-146 (2019).

358. Neelu N, Pandey N, Chakrabarti S. Morphology of highly stable lead-free hybrid organic–inorganic double perovskites $(CH_3NH_3)_2XBiCl_6$ (X = K, Na, Ag) for solar cell applications. *Journal of Materials Science* **58**, 11139-11158 (2023).

359. Ghosh SS, Sil A. Enhancement of photoresponse property of perovskite solar cell by aluminium chloride ($AlCl_3$). *Semiconductor Science and Technology* **33**, 055002 (2018).

360. Jin KX*, et al.* Tunable photovoltaic effect and solar cell performance of self-doped perovskite $SrTiO_3$. *AIP Advances* **2**, (2012).

361. Zhu L*, et al.* Achieving 20.8% organic solar cells via additive-assisted layer-by-layer fabrication with bulk p-i-n structure and improved optical management. *Joule* **8**, 3153-3168 (2024).

362. Sun Y*, et al.* π-Extended Nonfullerene Acceptor for Compressed Molecular Packing in Organic Solar Cells To Achieve over 20% Efficiency. *Journal of the American Chemical Society* **146**, 12011-12019 (2024).

363. Guan S*, et al.* Self-Assembled Interlayer Enables High-Performance Organic Photovoltaics with Power Conversion Efficiency Exceeding 20%. *Advanced Materials* **36**, 2400342 (2024).

364. Guo C*, et al.* Light-induced quinone conformation of polymer donors toward 19.9% efficiency organic solar cells. *Energy & Environmental Science* **17**, 2492-2499 (2024).

365. Fu J*, et al.* Rational molecular and device design enables organic solar cells approaching 20% efficiency. *Nature Communications* **15**, 1830 (2024).

366. Chen T*, et al.* Compromising Charge Generation and Recombination of Organic Photovoltaics with Mixed Diluent Strategy for Certified 19.4% Efficiency. *Advanced Materials* **35**, 2300400 (2023).

367. Ren Y*, et al.* Hydroxamic acid pre-adsorption raises the efficiency of cosensitized solar cells. *Nature* **613**, 60-65 (2023).

368. Kakiage K, Aoyama Y, Yano T, Oya K, Fujisawa J-i, Hanaya M. Highly-efficient dye-sensitized solar cells with collaborative sensitization by silyl-anchor and carboxy-anchor dyes. *Chemical Communications* **51**, 15894-15897 (2015).

369. Ji J-M, Zhou H, Eom YK, Kim CH, Kim HK. 14.2% Efficiency Dye-Sensitized Solar Cells by Co-sensitizing Novel Thieno[3,2-b]indole-Based Organic Dyes with a Promising Porphyrin Sensitizer. *Advanced Energy Materials* **10**, 2000124 (2020).

370. Grobelny A*, et al.* A Molecularly Tailored Photosensitizer with an Efficiency of 13.2% for Dye-Sensitized Solar Cells. *Advanced Materials* **35**, 2207785 (2023).

371. Zhou H*, et al.* Synergistic Effect of Size-Tailored Structural Engineering and Postinterface Modification for Highly Efficient and Stable Dye-Sensitized Solar Cells. *ACS Applied Materials & Interfaces* **15**, 43835-43844 (2023).

372. Han L*, et al.* High-efficiency dye-sensitized solar cell with a novel co-adsorbent. *Energy & Environmental Science* **5**, 6057-6060 (2012).

373. Zeng K*, et al.* Efficient solar cells sensitized by a promising new type of porphyrin: dye-aggregation suppressed by double strapping. *Chemical Science* **10**, 2186-2192 (2019).

374. Silva JPB*, et al.* Perovskite ferroelectric thin film as an efficient interface to enhance the photovoltaic characteristics of Si/SnOx heterojunctions. *Journal of Materials Chemistry A* **8**, 11314-11326 (2020).

375. Nechache R*, et al.* Bandgap tuning of multiferroic oxide solar cells. *Nature Photonics* **9**, 61-67 (2015).

376. Zhang Y, Sun H, Yang C, Su H, Liu X. Modulating Photovoltaic Conversion Efficiency of BiFeO3-Based Ferroelectric Films by the Introduction of Electron Transport Layers. *ACS Applied Energy Materials* **2**, 5540-5546 (2019).

377. Spanier JE*, et al.* Power conversion efficiency exceeding the Shockley–Queisser limit in a ferroelectric insulator. *Nature Photonics* **10**, 611-616 (2016).

378. Li X, Xuemin W, Liping P, Kuibao Z, Weidong W, and Tang Y. Ferroelectric thin film on a silicon-based pn junction: Coupling photovoltaic properties. *Ferroelectrics* **500**, 250-258 (2016).

379. Fix T. Oxide and Ferroelectric Solar Cells. In: *Advanced Micro- and Nanomaterials for Photovoltaics*) (2019).

380. Tan Z*, et al.* Thinning ferroelectric films for high-efficiency photovoltaics based on the Schottky barrier effect. *NPG Asia Materials* **11**, (2019).

381. Alta Devices Sets Solar World Record – NASA Selects Alta Devices, available at: https://www.altadevices.com/solar-world-record-nasa-selects-alta-devices/ (accessed 28 April 2025).

382. National Renewable Energy Laboratory, Best Research-Cell Efficiency Chart, available at: https://www.nrel.gov/pv/cell-efficiency (accessed 28 April 2025).

383. Scarpulla MA*, et al.* CdTe-based thin film photovoltaics: Recent advances, current challenges and future prospects. *Solar Energy Materials and Solar Cells* **255**, (2023).
