# Supplementary material for: A self-reliance framework for identifying strategic advanced materials
Source: Nat Commun. 2026 May 21;17:6696. doi: 10.1038/s41467-026-73294-8 (PMC13385709; doi:10.1038/s41467-026-73294-8)
Supplement: Supplementary file 2 — Transparent Peer Review file [file 41467_2026_73294_MOESM2_ESM.pdf]

nature portfolio

Peer Review File

A self-reliance framework for identifying strategic advanced materials

Corresponding Author: Dr Adam Kelly

This file contains all reviewer reports in order by version, followed by all author rebuttals in order by version.

Version 0:

Reviewer comments:

Reviewer #1

(Remarks to the Author)
The paper has a very interesting and timely topic, especially in the light of current global developments. The focus on European resilience fits current policy developments and narratives, e.g. with the recent Clean Industrial Deal and the Draghi report published last year, both aiming to increase the resilience of European industry. It also aligns well with last year's Critical Raw Materials Act, with a lot of attention for minerals. Extending this focus to nanomaterials and compounds is a valuable contribution to the field. The paper is generally well-written.

The introduction clearly communicates the urgency and the relevance of the study. I miss a bit of background on nanotechnology and advanced materials. For what key technologies are these materials crucial? You mention frontier research, but do not further explain what this means and entails. This could be quickly touched upon before addressing the research gap. (Frontier research is mentioned again in the section 'supply resilience: the elements', also without any further explanation.).

Figure 1b with import reliance against elemental abundance is very interesting, showing that even for very much abundant elements the import reliance is often high.

I feel the link between import reliance and supply resilience is a bit oversimplified in the paper. Obviously, supply resilience is generally higher if import reliance is lower. But how about diversification of the imports? Of course the resilience will be low if an element is 100% imported, from 1 country. But what if it's 100% imported from 100 different countries? The RE number will be the same, but the practical situation will be very different. This could be touched upon in the paper.

The formula for calculating the supply resilience of a compound is a very interesting addition, I guess many policy-makers and practitioners will be using this to indicate the resilience of supply chains.

The first paragraph of the section 'supply resilience: electronic components' is very technical and does not fit in very well with the rest of the paper. Some context is missing – why are these different materials so important? This section feels like a recital of different materials and their supply resilience. But what is the bigger picture here?

This is done much better for the section 'supply resilience: battery materials', and 'supply resilience: photovoltaics'. Consider rewriting the electronic components section to better match the other sections.

All figures look clear and are well-designed.

The SI is very extensive, rigorous and well-documented.

Some minor comments:
- 'As the performance of enormous number of nanomaterials has now been investigated,' number should be numberS
- 'In many cases, no primary deposits exist within Europe but, once imported, can be processed domestically, with an example being borates imported from Turkey and processed in Germany.', sentence not correct

Reviewer #2

(Remarks to the Author)
This paper demonstrates methodological and data-related innovation, particularly in its attempt to apply the supply resilience framework to critical minerals and related product chains, which is of significant value. However, the current analysis remains insufficient in several key aspects, including differentiation of elemental sources, transmission mechanisms of compound and upstream risks, technological complexity, and diversity of product pathways. Expanding and refining these areas would greatly enhance the explanatory power and representativeness of the study, making it more aligned with Nature Communications' standards for systemic and cross-disciplinary research.
1. Supply resilience: The elements
The current assessment assigns equal weights to different sources of the same element, without accounting for the actual differences in risk. For instance, lithium sourced from China, Australia, or Bolivia entails very different supply risks. Treating them uniformly introduces clear bias in resilience evaluation. It is therefore necessary to refine the treatment of source-specific risks, for example by weighting according to import shares or geopolitical relationships.
2. Supply resilience: Compounds, electronic components, battery materials, photovoltaics
The geometric mean method based on stoichiometry overlooks important complexities. First, many elements are co-produced, so the resilience of a compound is often determined by its least resilient element. Second, the analysis neglects the production–trade transmission effect. For example, if Europe imports compound X from China (containing lithium, cobalt, and nickel), its resilience should not be assessed solely based on the elemental composition, but should trace back to China's upstream dependencies—such as Congolese cobalt or Bolivian lithium. In other words, compound-level resilience should be evaluated in relation to the supplier country's upstream risks, not merely Europe's direct import structure.
3. Technological complexity
Along the path "elements ⟶ compounds ⟶ electronic components ⟶ battery materials ⟶ photovoltaic modules," technological complexity increases step by step. Supply resilience cannot be adequately captured by elemental sourcing alone. The substitutability of components and critical equipment, as well as mutual constraints across different technological stages, must be considered. For example, while China may be Europe's primary risk source for lithium batteries, Europe's technological capacity in certain areas may impose countervailing constraints on China. These interdependent relationships are complex, and even if they cannot be fully captured by a single indicator, they should be reflected in the analytical framework.
4. Diversity of product pathways
The article currently relies on a single product pathway, which limits its generalizability. While this may suffice for a specialist journal, Nature Communications as a cross-disciplinary journal requires research with stronger representativeness. Incorporating multiple industries and product pathways would enhance the robustness and broader applicability of the findings. Relying solely on a single pathway risks being interpreted as case-specific rather than indicative of systemic patterns.
5. Environmental and social dimensions
The framework of resilience should also integrate environmental and social constraints. Considering only geopolitical and economic aspects risks oversimplification, whereas environmental sustainability, community risks, and social acceptance are integral parts of resilience in the critical minerals context.
6. Visualization of the research framework
The authors propose the chain "elements ⟶ compounds ⟶ electronic components ⟶ battery materials ⟶ photovoltaic modules," which is a progressive technical pathway. However, the article currently describes this logic only in text, without intuitive presentation. I suggest adding an "input–output" style framework figure to illustrate the progressive relationships and the transmission of supply resilience across different stages. For example: elements as inputs are processed into compounds; compounds are then used in electronic components, battery materials, and photovoltaic modules; and resilience is transmitted or compounded across these stages. Such a figure would improve readability and highlight the analytical logic, particularly for a multidisciplinary audience.

Reviewer #3

(Remarks to the Author)
The manuscript under review presents a well-structured and carefully executed study, which merits recognition for both its clarity of presentation and the thoughtful implementation of the applied methodologies. While the analytical approaches employed are not novel in themselves, the choice of considered technologies and the particular combination of methods provide an original perspective that enriches the field of critical raw material research and thereby offers valuable insights. Especially the results section on the different technologies considered is well developed. This innovative synthesis is a noteworthy contribution and I would appreciate to see the work getting published!

Nonetheless, a significant limitation of the work lies in the neglection of closely related literature and its placement within the broader scholarly context. In particular, the extensive body of literature on raw material criticality – which in my opinion forms a crucial foundation for the assessment undertaken—has not been incorporated. Also, a discussion of how raw material supply resilience relates to raw material criticality is missing. This omission significantly reduces the robustness of the argumentation and weakens the connection of the study to ongoing academic discourse. This is especially obvious in the introduction of the work and in the discussion. But also within the work, several methodological choices/assumptions have been discussed before, however are not referenced in the manuscript. A more comprehensive engagement with existing research would substantially strengthen the manuscript and ensure that its contributions are situated appropriately within the field.

I recommend revising the manuscript with regard to related literature and therefore recommend a major revision. Please find

details below, with focus on the (in my opinion) missing literature. If you see certain point differently, I am looking forward to the discussion:

Major comments:
- P2: “certainty of supply was rarely an issue”. I agree that in the product development the certainty of material supply is/was of subordinate importance. However, the certainty of material supply is a very important topic for politics and in science since several decades. Since a publication of the NRC in 2008, this field is commonly termed “criticality assessments” https://nap.nationalacademies.org/resource/12034/critical_minerals_final.pdf . In the regard of resource criticality, also Graedel et al. 2012 should be referred to since this work is one of the foundational works of contemporary criticality assessments https://pubs.acs.org/doi/10.1021/es203534z .
- P2: Please be more precise where the current research has gaps. The last paragraph of p2. Starts with “This Article addresses this gap by introducing [..]”, however it is not get clear what research gap you mean. The paragraph before describes the problem of material scarcity especially from European perspective. Those are known facts that do not point out any research gaps. This relates to my major concern – the relevant literature is not sufficiently discussed in the introduction, which would facilitate the description of the existing research gaps.
- P3: Import Reliance – this is an frequently used indicator of resource criticality assessments, however the related scientific publications are not cited (e.g., Graedel et al., 2012, Blengini et al., 2017 (https://doi.org/10.1016/j.resourpol.2017.05.008))
- P4: “While a market share of 20% is considered concentrated in terms of competition [..]” what does this assumption base on? Several criticality assessments discussed this point.
- P4: “Supply resilience” – there is a bunch of scientific work centered around the topic of critical raw material resilience / supply resilience, such as Sprecher et al., 2015 (https://pubs.acs.org/doi/10.1021/acs.est.5b00206) which was one of the first works defining resilience in the context of critical raw materials, or Wietschel et al., 2025 (https://linkinghub.elsevier.com/retrieve/pii/S0921344925001284) who give an overview of works published with this focus and propose an integration of resilience and criticality assessments. Your proposed “supply resilience” metric only consist of IR and RIR and is quite simple compared to what other works suggested. Nevertheless, in my opinion, it could be suitable to indicate the likelihood of (or resilience against) sudden supply crises. However, in my opinion, you metric is not suitable to indicate a medium or long term risk (or resilience), since many more aspects are relevant in the medium or long term (such as distribution/concentration of reserves, the scarcity, demand development, …). I do not expect a changes to the proposed metric, but rather a better classification within the research landscape.
- P6: “RE > 80 as being strongly resilient against supply shocks, while those with an RE < 20 being weakly resilient” – please better justify these thresholds.
- P6: “Given that no single indicator exists to quantify the environmental impact of each element, [..]”. There are methodologies that quantify the EI on elemental level and there are several criticality assessments included indicators for quantifying environmental impacts (Graedel et al., 2012; Kolotzek et al., 2018 (https://linkinghub.elsevier.com/retrieve/pii/S0959652617331335). Again, it is not necessary to adopt those approaches, however, the assumption/approach has to be placed correctly within the scientific literature.
- P6: “resilience of a compound” there are works that discuss different options how to compute the criticality of “compounds”, e.g., Helbig et al., 2018 (http://linkinghub.elsevier.com/retrieve/pii/S0959652617324228)
- The chapter “discussion” completely lacks a discussion of related works. Furthermore, adding managerial/policy insights would strengthen this chapter.

Version 1:

Reviewer comments:

Reviewer #1

(Remarks to the Author)
The paper has improved considerably. Replacing the 'supply resilience' metric by a 'self-reliance' index helps a lot in conveying the main message of the paper. I'm also happy to see the authors integrated a concentration factor based on the dominant supplier fraction to better reflect the consequences of market concentrations. The newly added Figure 6 provides a clear graphical overview of how self-reliance works throughout the different technological stages assessed.

I'm not sure about the conceptualization of a critical nanomaterial as 'one that combines high technological performance with secure, local availability.' At least this does not correspond to how criticality is used in the field of critical raw materials, so to me it feels counterintuitive - when reading about a 'critical nanomaterial' I would assume this nanomaterial is associated with vulnerable supply instead of secure, local availability as defined by the authors. I do understand the authors want to move the focus away from vulnerable global supply chains to more awareness for local supply chain opportunities. However, since the concept of 'critical materials' has gained a lot of traction lately and people link this to supply risks, I don't know if this term would convey the desired message properly. Maybe strategic nanomaterial would be a better term, if aligning research, industry and policy is the goal?

Whether to address the latter point, is something for the authors to consider. I think this paper can be accepted for publication.

Reviewer #2

(Remarks to the Author)

The revised manuscript shows a clear improvement compared with the previous version, and the authors have made substantial enhancements in argumentation and structural organization. The topic is highly relevant to current discussions on the resilience of critical material supply chains and the sustainability of clean-technology pathways, and it proposes a conceptual framework that integrates material performance with supply-chain robustness. Further refinement would significantly enhance the transparency, robustness, and interpretability of the research.
1. First, the paper conceptually treats “substitution” and “recycling” as the two main strategies through which Europe mitigates critical material supply risks. However, in the construction of the self-reliance index (SE), the formula includes only recycling, while substitutability—which occupies a prominent role in the narrative—is not incorporated into the indicator system. This disconnects between the conceptual framework and the operationalization of the index warrants further explanation. The authors should clarify why substitutability is excluded from the SE index, whether it could in principle be incorporated, or whether it is intentionally treated as an independent dimension that should not enter the SE for theoretical reasons. Strengthening the consistency between the conceptual argumentation and the metric construction would improve the internal logic of the manuscript.
2. Second, the mathematical basis for the claim that the geometric mean “naturally weights” according to stoichiometry requires more sufficient justification. Because this geometric aggregation method forms the basis of the compound supply-risk indicator (SC), a more explicit explanation is needed. The current discussion relies more on intuition and lacks formal mathematical justification. The authors should further explain why the geometric mean is appropriate as a weighting method, how stoichiometric coefficients translate into differentiated weights within this framework, and link this choice to relevant methodological literature or provide exemplary derivations in an appendix. This would help readers understand under what conditions the SC indicator can be regarded as a valid measure of compound-level supply vulnerability.
3. Third, the self-reliance index (SE) fails to capture the political or institutional risks of exporting countries. The SE reflects Europe’s import dependence, supply concentration, and recycling capacity, but it does not account for the geopolitical characteristics of exporting countries—an aspect that is inconsistent with the manuscript’s own emphasis on geopolitical uncertainty, ideological differences, and strategic vulnerability. Supply risk depends not only on the scale and concentration of imports, but also on which countries they come from. Therefore, incorporating exporter instability or political stance into the SE, or at least discussing it as a supplementary factor, would make the indicator more aligned with real-world risk frameworks. Although this is not a strict requirement for acceptance, it would significantly enhance the analytical value and policy relevance of the results.
4. Fourth, the SC indicator is conceptually ambiguous when it involves ubiquitous elements such as oxygen. Since oxygen (and some other common elements) is assigned a default SE value of 100, its presence in stoichiometry inevitably increases the SC value and may obscure the true contribution of scarce or sensitive metals. Whether such elements should be included in the SC calculation remains conceptually unclear. The authors are advised to consider whether SE and SC should follow consistent logic and to conduct robustness checks by excluding ubiquitous elements or treating them neutrally, to demonstrate that SC reflects actual supply-chain risks rather than an artefact of chemical stoichiometry.
5. Fifth, the presentation of Figures 3c and 3d also raises clarity issues. The segmented vertical axes may cause misinterpretation, especially near the breakpoints where visual continuity is disrupted, potentially leading to misjudgment of relative magnitudes or the inference of unnatural trends. Providing clearer visual separation or explicitly explaining the handling of axis segmentation in the figure captions would substantially improve readability. In addition, whether the light-blue region represents the same critical threshold in all figures should be further clarified.
6. Finally, the practice of using the average value of all materials as the “critical performance threshold” requires further justification. Although this method is transparent and easy to apply, it may not reflect the true technical requirements of different material systems. Given that the authors have collected a large amount of data, exploring alternative threshold-setting approaches or more convincingly explaining why the mean has general applicability would enhance the technical relevance and practical significance of the manuscript.

Reviewer #3

(Remarks to the Author)
The authors comprehensively revised the manuscirpt and adressed my comments very well. During reviewing the revision, one additional question came up:

Page4: Fig. 1: The large difference between the IRc (EU) and IRc (Global) surprises me. Please verify if the circularity indicators EoL Recycling Input Rate (RIR) and EoL Recycling Rate (RR) have been used correctly (also see https://op.europa.eu/en/publication-detail/-/publication/871d2fd8-ee19-11e8-b690-01aa75ed71a1/language-en). For IRc (Global), the supplementary material refers to Gunn 2014 (Figure 3.4) for retrieving the global “recycling rates RR”. Gunn 2014, however, works with the EoL RR and not the EoL RIR, which can lead to a considerable difference. If the IRc (EU) is based on the indicator EoL RIR, and the IRc (Global) is based on the EoL RR, the difference between IRc (EU) and (Global) might stem from different indicator calculations rather than from different end-of-life recycling input rates.
o EoL RIR: “indicator measures, for a given raw material, how much of its input into the production system comes from the recycling of "old scrap" (or “end-of-life scrap”) i.e. scrap and waste derived from the treatment of products at their end-of-life (EOL)” (ref. Eurostat)”
o EoL RR: “end-of-life recycling rate the proportion of a metal that is discarded at the end of its life which is actually recycled.” (ref. Gunn 2014)

Figure 1a: the allocation of the element names to Import Reliance and IRc is hardly possible. A grid would probably solve this problem (e.g., light grey in the background

Version 2:

Reviewer comments:

Reviewer #1

(Remarks to the Author)
Again, the paper has improved considerably compared to the previous version. Also my final comment on the previous version of the manuscript has been addressed properly. I recommend publication of the manuscript.

Reviewer #2

(Remarks to the Author)
The author has addressed my questions and those of the other two reviewers very well. This article is very timely regarding the currently fragmented and geopolitically risky critical mineral supply chains. I agree with the publication of this article.

Reviewer #3

(Remarks to the Author)
Congratulations to the author's - all my points are suffently adressed wherefore I can recommend the manuscript for publicaiton.

Open Access This Peer Review File is licensed under a Creative Commons Attribution 4.0 International License, which permits use, sharing, adaptation, distribution and reproduction in any medium or format, as long as you give appropriate credit to the original author(s) and the source, provide a link to the Creative Commons license, and indicate if changes were made.
In cases where reviewers are anonymous, credit should be given to 'Anonymous Referee' and the source.
The images or other third party material in this Peer Review File are included in the article's Creative Commons license, unless indicated otherwise in a credit line to the material. If material is not included in the article's Creative Commons license and your intended use is not permitted by statutory regulation or exceeds the permitted use, you will need to obtain permission directly from the copyright holder.
To view a copy of this license, visit https://creativecommons.org/licenses/by/4.0/

Authors response

We thank the referees for the quick return of their reviews, and for the detailed and insightful comments on our study. Taking these comments into consideration, we have made substantial changes to the framing, purpose, and discussion of our framework which has strengthened the work considerably.

Our intention with using the phrase “supply resilience” was to identify those materials produced in Europe as they are more likely to have a resilient supply chain compared to those that are imported. However, as supply resilience is a well-defined term in the context of critical raw materials, we have reframed our metric as a “self-reliance index” as European sourcing is the meaning we are trying to capture. Such a definition covers the “geological” and “technological” availability factors that comprise part of a supply risk analysis, which allows us to situate this methodology in field of critical assessments.

We have also clarified the need for such an index. In particular, funding programs (e.g., Horizon Cluster grants) are now nudging applicants into reducing their reliance on non-European sources. As many grant applications use nanomaterials to drive device advances, a resource and material selection tool is required to provide information on which nanomaterials are sourceable in Europe or which elements constituting a nanomaterial represent vulnerabilities and should be substituted for ones that are locally available.

This reworking of the original manuscript has clarified for us the separate classification that nanomaterials require owing to their use. In analogy with designating raw materials as critical, we have introduced a category of “critical” for nanomaterials that combine a high technological performance (e.g., mobility or theoretical capacity) with the ability to synthesise them from locally available elements. We include an example on how to calculate the thresholds for performance and self-reliance, noting to the reader/policymaker that they can be set with specific applications in mind.

In addition to giving our paper a new title to reflect these changes, we have rewritten the discussion section to now contain an in-depth discussion. We show how the self-reliance index can propagate from elements to compounds to devices, and discuss the limitations of this approach that will be addressed in future work, and suggest a definition for what we believe makes a nanomaterial “critical.”

Reviewer #1 (Remarks to the Author):

The paper has a very interesting and timely topic, especially in the light of current global developments. The focus on European resilience fits current policy developments and narratives, e.g. with the recent Clean Industrial Deal and the Draghi report published last year, both aiming to increase the resilience of European industry. It also aligns well with last year’s Critical Raw Materials Act, with a lot of attention for minerals. Extending this focus to nanomaterials and compounds is a valuable contribution to the field. The paper is generally well-written.

The introduction clearly communicates the urgency and the relevance of the study. I miss a

bit of background on nanotechnology and advanced materials. For what key technologies are these materials crucial? You mention frontier research, but do not further explain what this means and entails. This could be quickly touched upon before addressing the research gap. (Frontier research is mentioned again in the section 'supply resilience: the elements', also without any further explanation.).

Our meaning with "frontier research" was performance breakthroughs that are driven by nanomaterials, such as silicon nanoparticles in battery electrodes or indium selenide in thin-film transistors. We acknowledge that this phrase was vague and have removed it from the manuscript, with the idea rephrased as "breakthroughs in advanced technologies such as semiconductors, batteries, and photovoltaics" or similar.

Figure 1b with import reliance against elemental abundance is very interesting, showing that even for very much abundant elements the import reliance is often high.

We fully agree, this is the relationship that motivated the study.

I feel the link between import reliance and supply resilience is a bit oversimplified in the paper. Obviously, supply resilience is generally higher if import reliance is lower. But how about diversification of the imports? Of course the resilience will be low if an element is 100% imported, from 1 country. But what if it's 100% imported from 100 different countries? The RE number will be the same, but the practical situation will be very different. This could be touched upon in the paper.

In line with a comment from another reviewer, we have updated the metric to include a concentration factor based on the dominant supplier fraction. This factor is included as an exponent to represent the disproportionate (nonlinear) effect a dominant supplier has on imports. We have also given supplier concentration more prominence by moving Figure S3 to Figure 2a and including lines of constant S_E to illustrate how the equation relates the self-reliance index to the variables. Interestingly, in cases where an element is close to 100% imported, it tends to come from a very small number of countries, and with one country usually having significant dominance. We have updated all figures with the new values.

The formula for calculating the supply resilience of a compound is a very interesting addition, I guess many policy-makers and practitioners will be using this to indicate the resilience of supply chains.

The first paragraph of the section 'supply resilience: electronic components' is very technical and does not fit in very well with the rest of the paper. Some context is missing – why are these different materials so important? This section feels like a recital of different materials and their supply resilience. But what is the bigger picture here? This is done much better for the section 'supply resilience: battery materials', and 'supply resilience: photovoltaics'. Consider rewriting the electronic components section to better match the other sections.

We thank the reviewer for pointing this out, this section was very heavy on jargon. It has now been substantially rewritten to give a higher-level description of what is presented.

All figures look clear and are well-designed.

The SI is very extensive, rigorous and well-documented.

Some minor comments:
- 'As the performance of enormous number of nanomaterials has now been investigated,' number should be numberS

We have rewritten the introduction and this sentence has been replaced.

- 'In many cases, no primary deposits exist within Europe but, once imported, can be processed domestically, with an example being borates imported from Turkey and processed in Germany.', sentence not correct

We have rewritten this as "However, once the primary ore is imported, it can be processed and purified domestically, with an example being borates imported from Turkey and refined into boron in Germany."

Reviewer #2 (Remarks to the Author):

This paper demonstrates methodological and data-related innovation, particularly in its attempt to apply the supply resilience framework to critical minerals and related product chains, which is of significant value. However, the current analysis remains insufficient in several key aspects, including differentiation of elemental sources, transmission mechanisms of compound and upstream risks, technological complexity, and diversity of product pathways. Expanding and refining these areas would greatly enhance the explanatory power and representativeness of the study, making it more aligned with Nature Communications' standards for systemic and cross-disciplinary research.

1. Supply resilience: The elements
The current assessment assigns equal weights to different sources of the same element, without accounting for the actual differences in risk. For instance, lithium sourced from China, Australia, or Bolivia entails very different supply risks. Treating them uniformly introduces clear bias in resilience evaluation. It is therefore necessary to refine the treatment of source-specific risks, for example by weighting according to import shares or geopolitical relationships.

In line with comments from the other reviewers, not only have we reframed our metric as a self-reliance index, we have also included the dominant supplier fraction as an exponent to non-linearly weight the index following the diversity of suppliers and created a new Figure 2a to reflect the importance of import shares. We have updated all figures accordingly and included a significant amount of new discussion surrounding our reasoning.

2. Supply resilience: Compounds, electronic components, battery materials, photovoltaics
The geometric mean method based on stoichiometry overlooks important complexities. First, many elements are co-produced, so the resilience of a compound is often determined by its

least resilient element. Second, the analysis neglects the production–trade transmission effect. For example, if Europe imports compound X from China (containing lithium, cobalt, and nickel), its resilience should not be assessed solely based on the elemental composition, but should trace back to China's upstream dependencies—such as Congolese cobalt or Bolivian lithium. In other words, compound-level resilience should be evaluated in relation to the supplier country's upstream risks, not merely Europe's direct import structure.

This is indeed the case for imported compounds, such as those found in battery electrodes, but it would be rather difficult to trace the ultimate sources of each element in a device owing to supplier diversity, the lack of publicly available data on corporate supply lines, and the transnational nature of manufacturing. However, we have not been clear in our use of the word "compound." In this work, we use compound to mean the composition of a nanomaterial that can be synthesised in Europe by combining imported and/or domestically sourced elements.

As import reliance data are available for individual elements more readily than imported compounds, this means that combining scores for individual elements using the geometric means can provide useful information on Europe's dependence on foreign sources on an elemental level. Using the geometric mean means the self-reliance index of a compound will be determined by its most vulnerable element which, in the case of an element with an index of 0, sends the index of the compound or entire device to 0.

We have clarified the reasoning behind using the geometric mean over arithmetic approaches, and also demonstrated how this allows us to propagate the self-reliance index up from elements to compounds (and now devices following this reviewer's comment below), and we now discuss this in detail with examples in the discussion section.

3. Technological complexity

Along the path "elements ⟶ compounds ⟶ electronic components ⟶ battery materials ⟶ photovoltaic modules," technological complexity increases step by step. Supply resilience cannot be adequately captured by elemental sourcing alone. The substitutability of components and critical equipment, as well as mutual constraints across different technological stages, must be considered. For example, while China may be Europe's primary risk source for lithium batteries, Europe's technological capacity in certain areas may impose countervailing constraints on China. These interdependent relationships are complex, and even if they cannot be fully captured by a single indicator, they should be reflected in the analytical framework.

This was the problem with our original framing using the term "supply resilience". Our intention was to identify as resilient those elements extractable in Europe to provide researchers with a material selection tool. However, a supply resilience analysis of course covers multiple other factors beyond geographical and technological availability.

As such, we have reframed the analysis as a self-reliance index. Owing to the permutability of nanomaterial compositions, substitutability is at the core of this framework. We show in Figures 3 to 5 that many high performing materials that depend on imports have locally sourced alternatives with similar performance. We have given this substitutability a significant

amount of discussion and have placed it at the core of the definition of a critical nanomaterial. In addition, as mentioned above, we now show how self-reliance can propagate up through technological complexity in a newly added Figure 6.

4. Diversity of product pathways
The article currently relies on a single product pathway, which limits its generalizability. While this may suffice for a specialist journal, Nature Communications as a cross-disciplinary journal requires research with stronger representativeness. Incorporating multiple industries and product pathways would enhance the robustness and broader applicability of the findings. Relying solely on a single pathway risks being interpreted as case-specific rather than indicative of systemic patterns.

This is a great point. Our study is designed to be a flexible tool and all data are provided for readers to calculate self-reliance indexes for their own bespoke materials and devices that were not covered under the electronics discussed here. We have now included a section in the Discussion outlining three other product pathways in fields beyond electronics where nanomaterials play a key role. It is our goal to explore these in future work.

5. Environmental and social dimensions
The framework of resilience should also integrate environmental and social constraints. Considering only geopolitical and economic aspects risks oversimplification, whereas environmental sustainability, community risks, and social acceptance are integral parts of resilience in the critical minerals context.

This again was a problem with using the "supply resilience" wording. Supply resilience in the criticality literature contains many components, but our goal is to provide a material selection tool that allows readers to choose materials that Europe is self-reliant in. However, we want to include an environmental discussion and, following the newly cited works in the criticality literature on environmental considerations, we use the Environmental Performance Index to rate the dominant supplier for each element as a measure of environmental sustainability. The EPI contains a wide variety of indicators, and countries with higher EPI scores are more likely to engage in positive environmental and social practices. We have included a paragraph discussing the relationship between self-reliance index and the EPI under the section "Self-reliance: The elements."

6. Visualization of the research framework
The authors propose the chain "elements ⟶ compounds ⟶ electronic components ⟶ battery materials ⟶ photovoltaic modules," which is a progressive technical pathway. However, the article currently describes this logic only in text, without intuitive presentation. I suggest adding an "input–output" style framework figure to illustrate the progressive relationships and the transmission of supply resilience across different stages. For example: elements as inputs are processed into compounds; compounds are then used in electronic components, battery materials, and photovoltaic modules; and resilience is transmitted or compounded across these stages. Such a figure would improve readability and highlight the analytical logic, particularly for a multidisciplinary audience.

This makes a great addition. We have now included a new figure, Figure 6, to show how self-

reliance can be transmitted up to the device level using the example of a perovskite solar cell. Along with the new text in the discussion section, this extension of the self-reliance index allows us to illustrate how a device can be rated and also allows the identification of specific components that represent vulnerabilities and should be targeted for substitution.

Reviewer #3 (Remarks to the Author):

The manuscript under review presents a well-structured and carefully executed study, which merits recognition for both its clarity of presentation and the thoughtful implementation of the applied methodologies. While the analytical approaches employed are not novel in themselves, the choice of considered technologies and the particular combination of methods provide an original perspective that enriches the field of critical raw material research and thereby offers valuable insights. Especially the results section on the different technologies considered is well developed. This innovative synthesis is a noteworthy contribution and I would appreciate to see the work getting published!

Nonetheless, a significant limitation of the work lies in the neglection of closely related literature and its placement within the broader scholarly context. In particular, the extensive body of literature on raw material criticality – which in my opinion forms a crucial foundation for the assessment undertaken—has not been incorporated. Also, a discussion of how raw material supply resilience relates to raw material criticality is missing. This omission significantly reduces the robustness of the argumentation and weakens the connection of the study to ongoing academic discourse. This is especially obvious in the introduction of the work and in the discussion. But also within the work, several methodological choices/assumptions have been discussed before, however are not referenced in the manuscript. A more comprehensive engagement with existing research would substantially strengthen the manuscript and ensure that its contributions are situated appropriately within the field.

I recommend revising the manuscript with regard to related literature and therefore recommend a major revision. Please find details below, with focus on the (in my opinion) missing literature. If you see certain point differently, I am looking forward to the discussion:

We completely agree with the reviewer and thank them for pointing out this oversight.

Major comments:
- P2: "certainty of supply was rarely an issue". I agree that in the product development the certainty of material supply is/was of subordinate importance. However, the certainty of material supply is a very important topic for politics and in science since several decades. Since a publication of the NRC in 2008, this field is commonly termed "criticality assessments" https://nap.nationalacademies.org/resource/12034/critical_minerals_final.pdf

With this phrase, we had in mind a more lab-based level. Very rarely do papers reporting the devices contained in this work address the availability of their material, and if they do, crustal abundance is used as the measure. As we show in Figure 1b, is not a useful indicator. While this phrase has since been removed in the rewrite, the certainty of supply issue is discussed under the motivation for the self-reliance index.

- In the regard of resource criticality, also Graedel et al. 2012 should be referred to since this work is one of the foundational works of contemporary criticality assessments https://pubs.acs.org/doi/10.1021/es203534z .

This work and others by Graedel have now been cited at least once throughout.

- P2: Please be more precise where the current research has gaps. The last paragraph of p2. Starts with "This Article addresses this gap by introducing [..]", however it is not get clear what research gap you mean. The paragraph before describes the problem of material scarcity especially from European perspective. Those are known facts that do not point out any research gaps. This relates to my major concern – the relevant literature is not sufficiently discussed in the introduction, which would facilitate the description of the existing research gaps.

Our goal is to provide a tool for material selection based on the implicit assumption that European-sourced materials have supply chains that are inherently more secure than imported materials. Funding programmes such as the clusters under Horizon Europe now contain sections such as "**Technological leadership and reduced strategic dependencies**: Ensuring Europe can develop, produce, and deploy advanced materials without overreliance on non-EU suppliers." However, there is no resource available for applications containing nanomaterials to identify how reliant they may be on imports. By rephrasing the "supply resilience" metric as the "self-reliance" index, we are clearer in our intent and can more easily show why this work is needed, as well as avoiding the confusion with the established definitions of supply resilience. We can additionally show how this index relates to, and is built from, the methodologies in the existing literature. We have rewritten the introduction to clarify the motivation.

- P3: Import Reliance – this is an frequently used indicator of resource criticality assessments, however the related scientific publications are not cited (e.g., Graedel et al., 2012, Blengini et al., 2017 (https://doi.org/10.1016/j.resourpol.2017.05.008))

These works have now been cited where IR is introduced in the "Elemental abundance and European reliance on imports" section, in addition to further citations in the new discussion section.

- P4: "While a market share of 20% is considered concentrated in terms of competition [..]" what does this assumption base on? Several criticality assessments discussed this point.

We were basing this on the highest values of industry concentration using employment metrics available on Eurostat, however, we acknowledge that this does not specifically relate to a market share in terms of competition. We have now cited Article 102 of the Treaty on the Functioning of the European Union where a relationship between market share and dominance has emerged in the jurisprudence of European courts, and have added new text to describe Figure 1c.

- P4: "Supply resilience" – there is a bunch of scientific work centered around the topic of critical raw material resilience / supply resilience, such as Sprecher et al., 2015

(https://pubs.acs.org/doi/10.1021/acs.est.5b00206) which was one of the first works defining resilience in the context of critical raw materials, or Wietschel et al., 2025 (https://linkinghub.elsevier.com/retrieve/pii/S0921344925001284) who give an overview of works published with this focus and propose an integration of resilience and criticality assessments. Your proposed "supply resilience" metric only consist of IR and RIR and is quite simple compared to what other works suggested. Nevertheless, in my opinion, it could be suitable to indicate the likelihood of (or resilience against) sudden supply crises. However, in my opinion, you metric is not suitable to indicate a medium or long term risk (or resilience), since many more aspects are relevant in the medium or long term (such as distribution/concentration of reserves, the scarcity, demand development, ...). I do not expect a changes to the proposed metric, but rather a better classification within the research landscape.

We hope we have provided this classification with the term "self-reliance index." In the context of nanomaterials, the relationship between self-reliance and resilience is via the inherent substitutability of the compounds. This means similar device performances can be achieved using local elements, once they can be readily identified. For example, WS_2 has very similar properties to MoS_2 and wolframite is widely available across Europe.

The simplicity of our index can be seen as a strength. We want to include all elements from hydrogen to bismuth so all (relevant) nanomaterials can be indexed under this framework and, for consistency, we need the same data for all elements. In aiming to assess self-reliance, data for IR, RIR, and dominant supplier fraction are available for almost all elements (with global supply standing in where not) and capture to a first approximation whether Europe is self-reliant. From Sprecher et al., resilience often refers to the flexibility of a supply chain to absorb short term price action. In a similar sense, by building self-reliance in nanomaterials to act as a hedge against the effects of a crisis, we would make supply chains more resilient, in addition to reducing environmental damage and exploitation.

However, we believe a self-reliance framework can support both short- and long-term planning, where choosing a European-sourced element means it will be less susceptible to short-term shocks (e.g., price swings), while also allowing funding programs to direct resources into longer term projects whose proposed nanomaterials can be sourced in Europe and to "pick winners" for development or pilot lines.

- P6: "RE > 80 as being strongly resilient against supply shocks, while those with an RE < 20 being weakly resilient" – please better justify these thresholds.

These were arbitrary and such grades have been omitted under the self-reliance index. We now include critical import fraction and dominant supplier fraction thresholds for defining the threshold for "self-reliance" for a critical nanomaterial. The grading in intervals of 20 in Figure 2a and Table 1 is a guide for the eye.

- P6: "Given that no single indicator exists to quantify the environmental impact of each element, [..]". There are methodologies that quantify the EI on elemental level and there are several criticality assessments included indicators for quantifying environmental impacts (Graedel et al., 2012; Kolotzek et al., 2018

(https://linkinghub.elsevier.com/retrieve/pii/S0959652617331335). Again, it is not necessary to adopt those approaches, however, the assumption/approach has to be placed correctly within the scientific literature.

The methodologies in the critical assessments suggested by the referee rely on the ecoinvent database, which requires a license to access. This database appears to be very rich in environmental impact information and is clearly vital for a comprehensive supply risk analysis. Roelich et al. and European Commission, Report of the Ad-hoc Working Group on defining critical raw materials also utilise the EPI, although in these cases it is to assess how a country's implementation of environmental regulations will affect mining operations. As such, this indicator is used as a proxy for governance.

In this work, we are using the EPI to provide additional motivation for moving to European suppliers. Figure S3 shows that elements that are sourced in Europe come from countries that score highly on the EPI and are therefore less likely to engage in environmentally damaging processes or have appreciable levels of human exploitation. We have clarified our reasoning for using the EPI in a new paragraph describing Figure S3 on p6.

- P6: "resilience of a compound" there are works that discuss different options how to compute the criticality of "compounds", e.g., Helbig et al., 2018 (http://linkinghub.elsevier.com/retrieve/pii/S0959652617324228)

The arithmetic mean and maximum approaches used in the works by Helbig are ones we initially considered. However, we set ourselves the criteria of when one component has a score of zero (i.e., is fully imported), it is a critical risk and the entire compound should score zero, which is only possible via multiplication. We take the reviewer's point that we should point out there are of course other methods for assessing compounds and have included this in a new paragraph on motivation for using the geometric mean (p7, "The strength of an index…")

- The chapter "discussion" completely lacks a discussion of related works. Furthermore, adding managerial/policy insights would strengthen this chapter.

We have completely rewritten the Discussion section to address the previous shortcomings.

Reviewer #1 (Remarks to the Author):

The paper has improved considerably. Replacing the 'supply resilience' metric by a 'self-reliance' index helps a lot in conveying the main message of the paper. I'm also happy to see the authors integrated a concentration factor based on the dominant supplier fraction to better reflect the consequences of market concentrations. The newly added Figure 6 provides a clear graphical overview of how self-reliance works throughout the different technological stages assessed.

I'm not sure about the conceptualization of a critical nanomaterial as 'one that combines high technological performance with secure, local availability.' At least this does not correspond to how criticality is used in the field of critical raw materials, so to me it feels counterintuitive - when reading about a 'critical nanomaterial' I would assume this nanomaterial is associated with vulnerable supply instead of secure, local availability as defined by the authors. I do understand the authors want to move the focus away from vulnerable global supply chains to more awareness for local supply chain opportunities. However, since the concept of 'critical materials' has gained a lot of traction lately and people link this to supply risks, I don't know if this term would convey the desired message properly. Maybe strategic nanomaterial would be a better term, if aligning research, industry and policy is the goal?

Whether to address the latter point, is something for the authors to consider. I think this paper can be accepted for publication.

The reviewer is 100% correct, “strategic” is the appropriate designation as phrasing suggestive of materials with a vulnerable supply is opposite of what we want to achieve. The defintion of ‘strategic’ for raw materials as those that are essential for essential for green, digital, defence, and space technologies also fits perfectly with those materials we wish to focus on.

In addition, we have become aware of a soon-to-be-published classification system for advanced materials, composed of 10 classes including biomaterials, ceramics, electronic and energy materials, smart and functional materials, nanomaterials, and advanced composites. We therefore want to indicate that the framework we report in this work is not limited to nanomaterials/electronic/energy materials, and is a more general advanced materials framework. We have updated the paper title to reflect this and have added a significant amount of new text to the Discussion to explain how our framework allows concepts used in raw material criticality assessments to be applied to advanced materials. We have also added an EU-style methodology for identifying strategic advanced materials to the Methods section.

Reviewer #2 (Remarks to the Author):

The revised manuscript shows a clear improvement compared with the previous version, and the authors have made substantial enhancements in argumentation and structural organization. The topic is highly relevant to current discussions on the resilience of critical material supply chains and the sustainability of clean-technology pathways, and it proposes a conceptual framework that integrates material performance with supply-chain robustness. Further refinement would significantly enhance the transparency, robustness, and interpretability of the research.

1. First, the paper conceptually treats “substitution” and “recycling” as the two main strategies through which Europe mitigates critical material supply risks. However, in the construction of the self-reliance index (SE), the formula includes only recycling, while substitutability—which occupies a prominent role in the narrative—is not incorporated into the indicator system. This disconnects between the conceptual framework and the operationalization of the index warrants further explanation. The authors should clarify why substitutability is excluded from the SE index, whether it could in principle be incorporated, or whether it is intentionally treated as an independent dimension that should not enter the SE for theoretical reasons. Strengthening the consistency between the conceptual argumentation and the metric construction would improve the internal logic of the manuscript.

We thank the reviewer for pointing out that substitutability has a distinct meaning and measure in criticality assessments, and that we have not been clear in its definition in the context of advanced materials/nanomaterials. The definition differs for an advanced material in that a heavily imported precursor can be substituted to produce a functionally similar materials (e.g., W for Mo to produce WS_2 instead of MoS_2) or an entire material can be substituted as an active layer in a device (e.g., $PtSe_2$ can be substituted with In_2O_3 as the semiconducting channel in a transistor without significant loss of mobility). Of course the assumptions here are that device architectures allow easy swapping between material families and that fabrication routes are compatible with different material compositions. We are currently unsure of how to quantify this but it will form the basis of our follow up work.

We have added a significant amount of new text to the Discussion section to properly elaborate the difference in treatment between raw and advanced materials, focusing on the value-added and substitutability metrics, and use this as the basis for our designation of a “strategic advanced material,” following the comment made by Reviewer 1 (paragraphs 4–7 in the Discussion section).

2. Second, the mathematical basis for the claim that the geometric mean “naturally weights” according to stoichiometry requires more sufficient justification. Because this geometric aggregation method forms the basis of the compound supply-risk indicator (SC), a more explicit explanation is needed. The current discussion relies more on intuition and lacks formal mathematical justification. The authors should further explain why the geometric mean is appropriate as a weighting method, how stoichiometric coefficients translate into differentiated weights within this framework, and link this choice to relevant methodological literature or provide exemplary derivations in an appendix. This would help readers understand under what conditions the SC indicator can be regarded as a valid measure of compound-level supply vulnerability.

We have included a discussion and more rigorous derivation in the new Supplementary Note 5 on why we are utilising the geometric mean, and use four cases to illustrate our point. We have also used this new discussion on compound calculations to dovetail with the reviewer’s comment below on ubiquitous elements and their treatment.

3. Third, the self-reliance index (SE) fails to capture the political or institutional risks of exporting countries. The SE reflects Europe’s import dependence, supply concentration, and recycling capacity, but it does not account for the geopolitical characteristics of exporting

countries—an aspect that is inconsistent with the manuscript's own emphasis on geopolitical uncertainty, ideological differences, and strategic vulnerability. Supply risk depends not only on the scale and concentration of imports, but also on which countries they come from. Therefore, incorporating exporter instability or political stance into the SE, or at least discussing it as a supplementary factor, would make the indicator more aligned with real-world risk frameworks. Although this is not a strict requirement for acceptance, it would significantly enhance the analytical value and policy relevance of the results.

We fully agree with the reviewer on the importance of geopolitical and institutional risks in supply chains. While indicators such as the world governance indicator are useful for critical and strategic raw materials, where some degree of import reliance is inevitable, they cannot capture unpredictable geopolitical events that can affect trade even with highly stable partners. For example, the EU has negotiated a critical minerals trade deal with Japan, a country with a high governance score but a regional conflict, such as an escalation between China and Taiwan, could directly affect EU-Japanese trade despite Japan's strong institutional stability.

We are sure such uncertainties in these indicators are known, but the primary purpose of the S_E and S_C indicators here is to identify high performance materials that Europe should be able to produce domestically, i.e. our "strategic advanced materials," whose goal is to mitigate geopolitical uncertainty by excluding non-European sourced materials from future technologies wherever possible. As governance indicators play an important role in criticality assessments, we have included new text on why we omit outward-looking geopolitical indicators in the inward-looking self-reliance index:

"When critical raw materials must be sourced externally, suppliers are preferentially selected from stable partner countries, with stability typically assessed through metrics like the world governance indicators that are incorporated into supply risk calculations. While this approach is necessary when imports are unavoidable, such indicators primarily reflect a country's internal institutional quality. They are less effective at capturing external geopolitical black-swan events that can disrupt trade irrespective of a supplier's domestic stability. For example, even a strategic partner like Japan, which scores highly on governance metrics, could see its trade with Europe severely restricted by a regional crisis, such as a conflict in the Taiwan Strait. A self-reliance index, by contrast, does not require such external indicators. Its purpose is instead to highlight elements that Europe can produce domestically and for which it can develop sovereign, or near-sovereign, supply chains."

4. Fourth, the SC indicator is conceptually ambiguous when it involves ubiquitous elements such as oxygen. Since oxygen (and some other common elements) is assigned a default SE value of 100, its presence in stoichiometry inevitably increases the SC value and may obscure the true contribution of scarce or sensitive metals. Whether such elements should be included in the SC calculation remains conceptually unclear. The authors are advised to consider whether SE and SC should follow consistent logic and to conduct robustness checks by excluding ubiquitous elements or treating them neutrally, to demonstrate that SC reflects actual supply-chain risks rather than an artefact of chemical stoichiometry.

We thank the reviewer for pointing this out. Our goal initially was to treat all elements evenly but this does end up masking the effect of a low-index element in an oxygen-rich compound,

e.g., V_2O_5. As many nanomaterials have substantial stoichiometric contributions from oxygen, we have taken oxygen as the test case for defining an element as "ubiquitous". Supplementary Note 5 now contains a plot showing the effect of including O in the compound calculations, showing significant score inflation for many compounds. We are therefore convinced that ubiquitous elements should be treated neutrally.

To the best of our knowledge, however, there is no definition for what constitutes ubiquity so we have suggested that a "ubiquitous" element in this context is one that is derived from planetary-scale reservoirs such as the atmosphere or oceans, is geographically and politically dispersed, is extractable using simple and widely implemented physical or chemical processes (e.g., those that can be taught at an undergraduate level), is routinely purifiable to high grade (≥ 4N) using non-proprietary technologies and without requiring significant energy inputs, and is not susceptible to long-term monopolisation through geological, chemical or technological advantages.

We have identified H, C, N, O, Na, Cl and the noble gases Ne, Ar, K, and Xe (He is excluded as its extraction is tied to specific gas fields) as ubiquitous and these are therefore treated neutrally in the compound calculations where present. All data has been updated to reflect this new consideration and we have added new text describing this in various places in the manuscript, with the most relevant on p5 as:

"This example raises the question of how ubiquitous elements such as oxygen should be treated as the value of S_C = 47 may be overinflated owing to oxygen constituting 2/5th of the compound. Existing criticality assessments focus on elements whose supply is constrained by geology, geography, or co-production and the concept of ubiquity has not required a rigorous definition. Advanced and nanostructured materials, however, differ fundamentally in that they intrinsically incorporate elements that are widely available and difficult to monopolise. In Supplementary Note 5, we define a ubiquitous element as one that is derived from planetary-scale reservoirs such as the air or oceans, is geographically and politically dispersed, is extractable and purifiable using simple and non-proprietary technologies, and is not susceptible to monopolisation. This definition identifies H, C, N, O, Na, Cl and the noble gases Ne, Ar, K, and Xe as ubiquitous elements. When calculating a compound self-reliance index via the geometric mean, these elements are therefore treated neutrally and do not contribute the overall S_C. In the case of Bi_2O_2Se above, this new consideration results in S_C = 29."

5. Fifth, the presentation of Figures 3c and 3d also raises clarity issues. The segmented vertical axes may cause misinterpretation, especially near the breakpoints where visual continuity is disrupted, potentially leading to misjudgment of relative magnitudes or the inference of unnatural trends. Providing clearer visual separation or explicitly explaining the handling of axis segmentation in the figure captions would substantially improve readability. In addition, whether the light-blue region represents the same critical threshold in all figures should be further clarified.

We admit some confusion to this comment as there are no breakpoints in the y axes of Figure 3c and 3d. Perhaps we are misunderstanding what the reviewer means, and we ask for further clarification on the presentation issue.

The critical threshold is different for each graph as it is calculated from the data within each graph. To make this explicit, we have now included the specific critical threshold value

inset in each graph, along with describing it in the caption. These values are also listed in Supplementary Note 19 along with the relevant technology's strategic nanomaterials.

6. Finally, the practice of using the average value of all materials as the "critical performance threshold" requires further justification. Although this method is transparent and easy to apply, it may not reflect the true technical requirements of different material systems. Given that the authors have collected a large amount of data, exploring alternative threshold-setting approaches or more convincingly explaining why the mean has general applicability would enhance the technical relevance and practical significance of the manuscript.

The reviewer is correct that the mean value may not reflect the specific technical requirements of every material system. By taking the mean of all materials used in a given technology, we can forgo establishing numerous, bespoke technical thresholds for individual material systems, which would require technical consensus for each class of materials and would be very complex. Instead, the mean provides a neutral and consistent method that can first-order identify materials that are promising for future technologies. The strategic question to answer is "Does this material perform above the observed average for the given technology?" If so, then it is worth further consideration for further development, with second-order considerations along the line of specific factors like ambient stability, toxicity, cost, etc.

To address the reviewer's point and strengthen our discussion, we have added the following justification to the text under the Discussion:

"We believe the mean is a neutral and consistent threshold for comparing diverse material systems that provides an objective, first-order indicator of relative performance. While it does not reflect the specific technical requirements of every material, it offers a practical and comparable benchmark. This approach answers a fundamental strategic question: does a material perform above the current average for its technology? If so, it merits further development as a promising candidate when second-order factors such as ambient stability or cost can be considered. Importantly, this critical performance threshold is not static. Each improvement in the state of the art raises the strategic bar, thereby rewarding materials that advance leading performance and penalising those that fail to keep pace with technological progress."

Reviewer #3 (Remarks to the Author):

The authors comprehensively revised the manuscirpt and adressed my comments very well. During reviewing the revision, one additional question came up:

Page4: Fig. 1: The large difference between the IRc (EU) and IRc (Global) surprises me. Please verify if the circularity indicators EoL Recycling Input Rate (RIR) and EoL Recycling Rate (RR) have been used correctly (also see https://op.europa.eu/en/publication-detail/-/publication/871d2fd8-ee19-11e8-b690-01aa75ed71a1/language-en). For IRc (Global), the supplementary material refers to Gunn 2014 (Figure 3.4) for retrieving the global "recycling rates RR". Gunn 2014, however, works with the EoL RR and not the EoL RIR, which can lead to a considerable difference. If the IRc (EU) is based on the indicator EoL RIR, and the IRc (Global)

is based on the EoL RR, the difference between IRc (EU) and (Global) might stem from different indicator calculations rather than from different end-of-life recycling input rates.
o EoL RIR: “indicator measures, for a given raw material, how much of its input into the production system comes from the recycling of "old scrap" (or “end-of-life scrap”) i.e. scrap and waste derived from the treatment of products at their end-of-life (EOL)” (ref. Eurostat)”
o EoL RR: “end-of-life recycling rate the proportion of a metal that is discarded at the end of its life which is actually recycled.” (ref. Gunn 2014)

Figure 1a: the allocation of the element names to Import Reliance and IRc is hardly possible. A grid would probably solve this problem (e.g., light grey in the background

We thank the reviewer for pointing out this significant discrepancy. Yes, the difference was between RR and RIR. We have searched the literature and have struggled to find global RIR values for a meaningful number of materials so have elected to omit this variable. Instead, we found an appreciable amount of data for the material collection rate and have used this to make the point that recapture is not the bottleneck, but separation and purification. A mining consortium summed this up as “Europe does not have a rare-earth problem, but a rare-earth processing problem.”

We have updated Figure 1a with these data, including guides for the eye, and added the following text:

“Figure 1a shows the import reliance for each element to meet demand, ranked from high to low (see Supplementary Note 2). Once an element enters the European material ecosystem, recapture and recycling can reduce the amount that must be sourced externally. We therefore define the critical import reliance, IR_C, as the proportion that must be imported and cannot be met with current recycling rates, such that $IR_C = IR(1-R)$, where R is the EU end-of-life recycling input rate. While some materials have high recycling input rates, such as rhenium at 50%, the average across all values is ~ 10%. The resulting IR_C for each element (where data exist) is shown by the light blue circles in Figure 1a. This low reinput rate exists despite high collection efficiency as the average collection rate, C, across elements is ~62%. This highlights a major systemic gap in that material is successfully gathered but not functionally returned to production. In a hypothetical, fully efficient circular scenario where all collected material is reinput ($R = C$), the import reliance for many fully imported elements could be drastically reduced. The potential IR_C in this scenario, calculated as $IR(1-C)$, is shown by the triangles in Figure 1a.

While the EU demonstrates high efficiency in collecting end-of-life products containing a broad mix of materials, this often results in non-functional recycling where materials are recovered but are processed into forms unsuitable for their original applications. This is due to the immense difficulty in selectively isolating specific elements from complex waste streams, as exemplified by neodymium (Nd) and samarium (Sm). These rare-earth have collection rates as high as 95% but the current reinput rate into new magnet production is virtually 0%. The disconnect between collection and reinput arises from a confluence of challenges, such as the technical complexity and cost of separating of target elements, the lack of EU-based refining capacity, and the economic non-viability of competing with primary virgin materials. Consequently, many collected elements are often downgraded into lower-value alloys or landfilled stockpiles, rather than re-entering the production cycle. This issue

has been addressed for strategic raw materials in the Critical Raw Material Act, which targets at least 25% of the EU's annual consumption for recycling by 2030."
